# Exploratory preferences explain the human fascination for imaginary worlds in fictional stories

**DOI:** 10.1038/s41598-023-35151-2

**Published:** 2023-05-28

**Authors:** Edgar Dubourg, Valentin Thouzeau, Charles de Dampierre, Andrei Mogoutov, Nicolas Baumard

**Affiliations:** Institut Jean Nicod, Département d’études cognitives, Ecole normale supérieure, Université PSL, EHESS, CNRS, Paris, France

**Keywords:** Cultural evolution, Evolutionary developmental biology, Human behaviour, Personality

## Abstract

Imaginary worlds are present and often central in many of the most culturally successful modern narrative fictions, be it in novels (e.g., *Harry Potter*), movies (e.g., *Star Wars*), video games (e.g., *The Legend of Zelda*), graphic novels (e.g., *One Piece*) and TV series (e.g., *Game of Thrones*). We propose that imaginary worlds are popular because they activate exploratory preferences that evolved to help us navigate the real world and find new fitness-relevant information. Therefore, we hypothesize that the attraction to imaginary worlds is intrinsically linked to the desire to explore novel environments and that both are influenced by the same underlying factors. Notably, the inter-individual and cross-cultural variability of the preference for imaginary worlds should follow the inter-individual and cross-cultural variability of exploratory preferences (with the personality trait Openness-to-experience, age, sex, and ecological conditions). We test these predictions with both experimental and computational methods. For experimental tests, we run a pre-registered online experiment about movie preferences (N = 230). For computational tests, we leverage two large cultural datasets, namely the Internet Movie Database (N = 9424 movies) and the Movie Personality Dataset (N = 3.5 million participants), and use machine-learning algorithms (i.e., random forest and topic modeling). In all, consistent with how the human preference for spatial exploration adaptively varies, we provide empirical evidence that imaginary worlds appeal more to more explorative people, people higher in Openness-to-experience, younger individuals, males, and individuals living in more affluent environments. We discuss the implications of these findings for our understanding of the cultural evolution of narrative fiction and, more broadly, the evolution of human exploratory preferences.

## Introduction

The cultural importance of imaginary worlds in contemporary societies cannot be overstated. *Star Wars* is the most lucrative media franchise in history. *Harry Potter* is the best-selling book series of all time. *Game of Thrones* holds the all-time audience record for a TV series. *One Piece* is the best-selling manga series in manga history. *The Avengers: Endgame* is the highest-grossing movie of all time. And *Zelda* is the best-selling video game series in the history of video games. They all have something in common: their creators developed an imaginary environment, that is, a fictional environment that does not actually exist, which is different from the real world, and that the consumers know to be partly or fully invented^1–4^. Moreover, such fictional stories with imaginary worlds appear better at building huge fan communities, as exemplified by the fact that lots of them turn into highly profitable multimedia franchises (e.g., the Wizarding World, the Marvel Cinematic Universe^1^). In other words, modern culture, from movies to clothing to theme parks to educational tools, increasingly revolves around imaginary worlds. Why is there such a massive interest in fantasy worlds? What lies behind this anthropological phenomenon?

In a theoretical paper, we proposed that imaginary worlds in fictional stories artificially trigger the human preference for exploration^5^. This preference is best described as an evolved cognitive mechanism that processes cues of new or information-rich environments as inputs and delivers an adaptive approach behavior as the output: it makes people environmentally curious and prompts directed exploration (i.e., exploration that aims at seeking and acquiring information about the environments, thus reducing uncertainty, as opposed to exploitation or random exploration^6–15^). This mechanism of environmental curiosity was selected because it enhanced fitness in ancestral environments, motivating humans to discover new habitats, new cooperative or sexual partners, resources such as food or water, and new fitness-relevant information^16, 17^. It explains, for instance, universal walking patterns integrating multiple changes of directions (i.e., Lévy walks, observed in hunter-gatherers^18^).

Because imaginary worlds are unknown fictional settings, they can be seen as new environments that consumers discover. As Tolkien put it himself, “part of the attraction of *The Lord of the Rings*”, and other fictions with imaginary worlds, relies on the “intrinsic feeling of reward” we experience when “viewing far off an unvisited island or the towers of a distant city” (letter to Colonel Worskett, 20 September 1963). This statement is very close to the one of Shigeru Miyamoto, the creator of *Zelda*, who reported that he “wanted to create a game world that conveyed the same feeling you get when you are exploring a new city for the first time” (1989). Following the same intuition, we hypothesized that humans are fascinated by imaginary worlds for the same reasons, and under the same circumstances, as they are motivated to explore new and unfamiliar environments^5^. It is equivalent to saying that imaginary worlds constitute a *superstimulus* of explorable environments^19, 20^, or that imaginary worlds are part of the *actual domain* of the cognitive mechanism that evaluates landscapes (whose proper domain is constituted of real cues of explorable environments), just like masks are part of the actual domain of the cognitive mechanism of human face recognition^21^.

Let us note that we differentiate between spatial exploration and cognitive exploration, and symmetrically between environmental curiosity and other domain-specific forms of curiosity (e.g., curiosity for how tools causally work^22^; see also^23^) because we reason that different forms of curiosity should not be sensitive to the same cues to orient attention and behavior. Relying on some recent molecular evidence, Hills^24^ has argued that spatial exploration and cognitive exploration are linked at the phylogenetic level: “What was once foraging in a physical space for tangible resources became, over evolutionary time, foraging in cognitive space for information related to those resources” . Here, we focus on environmental curiosity, that is, on the specific curiosity for environments that prompt spatial exploration while recognizing that exploratory preferences may exist as a more general cluster^25, 26^. For instance, there is experimental evidence that a preference for spatial exploration in a foraging task is associated with a preference for cognitive exploration in a problem-solving task^27^. In any circumstance, there seems to be a cognitive mechanism that specifically processes landscapes^17, 28, 29^.

In our theoretical paper, we reviewed the experimental evidence for the universal association between, on the one hand, novelty and learning opportunity and, on the other hand, exploratory choices (in the absence of extrinsic reward). For instance, participants in unfamiliar lab settings choose more exploratory options^30^. Consistently, when asked to choose new environments to explore, people use cues of perceptual or epistemic novelty to make their choice^31, 32^. In the brain, this is supported by the findings that novelty cues and learning opportunities lead to dopamine enhancement in midbrain areas^33–40^. There is also strong experimental evidence that humans universally favor more mysterious and more explorable environments when asked to report their preferences for landscapes^28, 41–43^.

In this paper, we further test the exploration hypothesis. In Study 1, we first explore the importance of environmental curiosity in structuring the landscape of contemporary fiction. Fictional stories can trigger a range of human motivations, from romantic love (e.g., in romance novels) to threat detection (e.g., in horror films) to causal reasoning (e.g., in detective novels). However, not all motivations are equally important to fiction: typically, eating food or discovering new smells are important to humans, but marginal in fiction, for reasons beyond the scope of this paper. Consequently, while fictional stories about food or perfumes do exist, they are relatively uncommon and not generally considered as belonging to a distinct genre. On the contrary, if exploration is one of the most important psychological factors in contemporary fiction, it should structure the landscape of contemporary fiction. We should observe that, when organized by semantic proximity, fictional stories with a strong emphasis on environmental curiosity, particularly those featuring imaginary worlds, should cluster together, and should cluster apart from other large, well-identified genres, such as romance or horror fiction. In Study 1, we also test that fictional characters in stories with imaginary worlds navigate their environments more than in other stories. This prediction relies on the idea that the psychology of fictional characters should be crafted to be consistent with their fictional environments: their evaluation of resourceful or informative landscapes should motivate *them* to explore more.

In studies 2 and 3, we develop a set of empirical predictions derived from our theory. In our theoretical paper^5^, we predicted that if the exploration theory is valid, then the attraction to imaginary worlds would be intrinsically linked to the desire to explore novel environments and that both would be influenced by the same underlying factors. In other words, we predict that each source of variability that explains inter-individual differences in the sensitivity of environmental curiosity should also explain inter-individual differences in the consumption of fictional stories with imaginary worlds. Before testing this mapping between cognitive variability and cultural variability, we further explain the origins and the mechanisms underlying inter-individual variability in environmental curiosity.

When and why should organisms decide to explore or not to explore? This question is best known as the evolutionary *exploration–exploitation trade-off* (^26, 44^; see^45, 46^ for reviews), which is dealt with differently in different species according to their life history strategies (e.g.,^47–51^). Crucially, this trade-off is also dealt with differently between individuals from the same species, including *Homo sapiens*. This is best seen in the inter-individual differences in spatial abilities^52^. We contend that sources of adaptive variability explain the inter-individual differences in people’s motivation to explore. First, we review the literature in evolutionary psychology and associated empirical evidence showing that the sensitivity of human environmental curiosity varies according to adaptive sources of variability. We select four of them that seem to explain a significant part of the variance while acknowledging that other factors could be added to increase the explained variance.

The importance of exploratory preferences varies at the inter-individual level according to some fixed personality traits, which are thought of as evolutionary strategies of specialization to some ecological or social niches^53–55^: people are genetically hardwired to be more or less curious. This is captured by the existence of a genetically inherited personality trait often called Openness-to-experience^56–58^. It constitutes one of the five dimensions within the Big Five, the model of human personality^57, 59^. The five dimensions that compose it (i.e., Openness, Conscientiousness, Extraversion, Agreeableness, and Neuroticism) have been designed to capture the universal variability of human personalities and behaviors: humans differ in the personality “scores” associated with each of these dimensions. The Big Five is considered the most widely accepted model of human personality today^60–64^.

The Openness trait is correlated with novelty-seeking behavior^38, 56, 65^, a preference for creativity^66–71^, spatial cognitive capacities^72–75^, a preference for using maps^76^, a preference to explore a system^77, 78^, and innovative deviations from observed demonstrations in learning tasks^79^. In other words, people higher in Openness-to-experience are overall more curious and explorative^80^. In the cultural domain, the Openness trait is correlated with the liking of adventure movies, fantasy movies, and science fiction movies^81^, the enjoyment of abstract art^82^, the preference for jazz, blues, classical, rock, alternative, and folk music^83–85^. It also correlates with some cultural practices reported by people, such as going to the theatre, art galleries, or museums^86^, or seeking novel food^87^. We, therefore, predict that *people higher in Openness should be, on average, fonder of fictional stories set in imaginary worlds.*

The sensitivity of exploratory preferences varies according to the developmental stage of the individual. Evolutionary developmental psychology explains why: younger individuals have more to learn from the world, so it is more adaptive for them to explore their environments and try to reduce knowledge gaps^88–90^. A complementary explanation posits that the evolutionary costs associated with exploration (e.g., resource shortage risk) are outweighed by parental caregiving investments^91, 92^. This can be seen as an adaptive feedback loop or as an adaptive developmental division of labor^93–95^.

There is already much experimental evidence that children are indeed more curious and eager to explore than adults (see^96^, for a review). Children are more explorative than adults in foraging tasks^97–99^, in bandit tasks^100^, in explanation-seeking tasks^101, 102^, in search tasks^103^, in decision-making tasks^104, 105^, in problem-solving tasks^106^, in causal-learning tasks^107, 108^, and in change-detection and visual search tasks^109^. Importantly, such behavioral data from experimental research and computational modeling show that children are not merely prone to random sampling behavior: they show clear patterns of *directed* exploration^110^. Another study found that intellectual curiosity is negatively correlated with age, after controlling for education level, sex, and culture^111^. In a foraging task, adolescents explored more and more optimally than adults did^112^, consistent with other findings suggesting that adolescents too are more motivated to explore novel, although risky, scenarios than adults^112–117^. In accordance with such findings, Openness has been shown to decline with age across countries^118–122^. We, therefore, predict that *younger people should be, on average, fonder of fictional stories set in imaginary worlds.*

The sensitivity of exploratory preferences varies according to an individual’s biological sex^123^. Selection pressures for exploratory preferences and abilities have been stronger for males in a lot of terrestrial vertebrates, and more specifically in a lot of mammalian species, because of different mating patterns for access to mates (caused by differences in reproductive variance between the sexes): in this view, spatial exploration is thought of as a male reproductive strategy^124–126^. For instance, in humans, there is evidence that, in the Tsimane (i.e., a forager-horticulturalists people in Bolivia), males travel more than females, and even more so during periods of intensive mate search^127^, but not earlier in ontogeny^75^.

Another evolutionary rationale posits that, in humans specifically, exploratory preferences and abilities contributed differentially to the reproductive success of males and females because of the sexual division in foraging activities: males would have specialized in solving spatial problems associated with hunting (which requires a propensity to explore unfamiliar environments) while females would have specialized in solving spatial problems associated with plant gathering (which requires a propensity to learn and remember object locations^128, 129^). Both rationales can explain why, in humans, males develop higher spatial abilities specifically related to exploration than females (see^130, 131^ for meta-analyses, see^132^ for a review) and navigate in wider ranges than females (^133, 134^, but see^135^).

Lastly, another complementary hypothesis proposes that males and females evolved different cognitive preferences and skills related to inventorying and classifying features when exploring the physical world, partly because of a male specialization in tool-use: ‘systemizing’, the drive to explore and understand systems, would have had a greater impact on the reproductive success of males than that of females^77, 136^. There is evidence that males score higher in systemizing^78, 137, 138^, that fetal testosterone levels positively correlate with a highly restricted range of interests, which is a marker of both high-systemizing and high-functioning autism^139^, and that there are more males with either higher systemizing-quotient or autistic traits who are interested in non-social domains of knowledge, such as engineering, mathematics, or science^78, 140–142^.

Overall, three different (and non-mutually exclusive) evolutionary hypotheses propose that some adaptive challenges human males specifically faced during their evolution (i.e., searching for mates, hunting, or using tools) led males to be more systematically curious about their spatial (and non-social) environments while leading females to be more systematically curious about their social environments. This is consistent with the findings that (1) in modern societies (here the United States, with experimental evidence from 320,000 participants) males score higher in the personality trait Openness-to-experience^143^, and (2) in hunter-gatherer societies (here, the Hadza of Tanzania, with evidence from GPS data), males explore more land, follow more sinuous paths, walk further per day^144^, and also perform better in three tests of spatial ability^145^. We, therefore, predict that *males should be, on average, fonder of fictional stories set in imaginary worlds.*

Finally, exploratory preferences are hypothesized to vary according to the local ecology of an individual. Exploration is most valuable and adaptive in more affluent, safer, and therefore more predictable environments^146–148^. Why? In unsafe and poor ecologies, exploration is very risky, notably because if exploration does not pay off, one is left with nothing. Relatedly, the opportunity costs of exploration are higher in scarcity because one is better off exploiting one’s environment to provide for more pressing needs. Conversely, in more affluent, safer, and predictable ecologies, such risks are lower: notably, when surrounded by more resources, individuals can afford to lose some of them in the short term^149^. Therefore, exploration is best defined as a ‘venture behavior’, that is, a preference for a high variance of rewards over short-term gains (as opposed to ‘hazardous behavior’^150^). Since organisms evolved in changing environments^151^, selection pressure would have favored exploratory preferences that are highly flexible to the local ecology, with time horizon as the crucial mediator^111, 149, 152^.

More generally, phenotypic plasticity enables an organism to adapt to new situations and environments by changing its behavior, rather than having to wait for genetic adaptations to occur. This flexibility in behavior can be an advantage in unpredictable or changing environments, allowing organisms to survive and reproduce more effectively. The behavioral effect of the local ecological cues on exploratory preferences, curiosity, and spatial search strategies^153^ is observed in a wide range of species, such as in orangutan^154–156^, honeybees^157^, parrots^158^, and chickadees^159^. It is parsimoniously hypothesized that it applies to humans^146, 160^.

At the individual level, there is empirical evidence that people living in richer families score higher in Openness-to-experience^161, 162^ and that people with higher income at one stage of their life are less likely to decrease in Openness-to-experience later on^163^. In a foraging task, people with more adverse childhood experiences remain in patches longer and, thus, explore less^164^. At the level of societies, recent empirical studies show that, across the world, people living in more affluent countries exhibit higher levels of openness to change and new experiences^165–167^. Finally, a recent study shows that *between-countries* differences in levels of causal learning and pretend play in children (i.e., the United States vs. Peru) are similar to those *within-countries* due to different socio-economic statuses (i.e., mixed-SES United States vs. low-SES United-States^168^). We, therefore, predict that *people living in more affluent local ecologies should, on average, be fonder of fictional stories set in imaginary worlds*.

**Figure 1:**
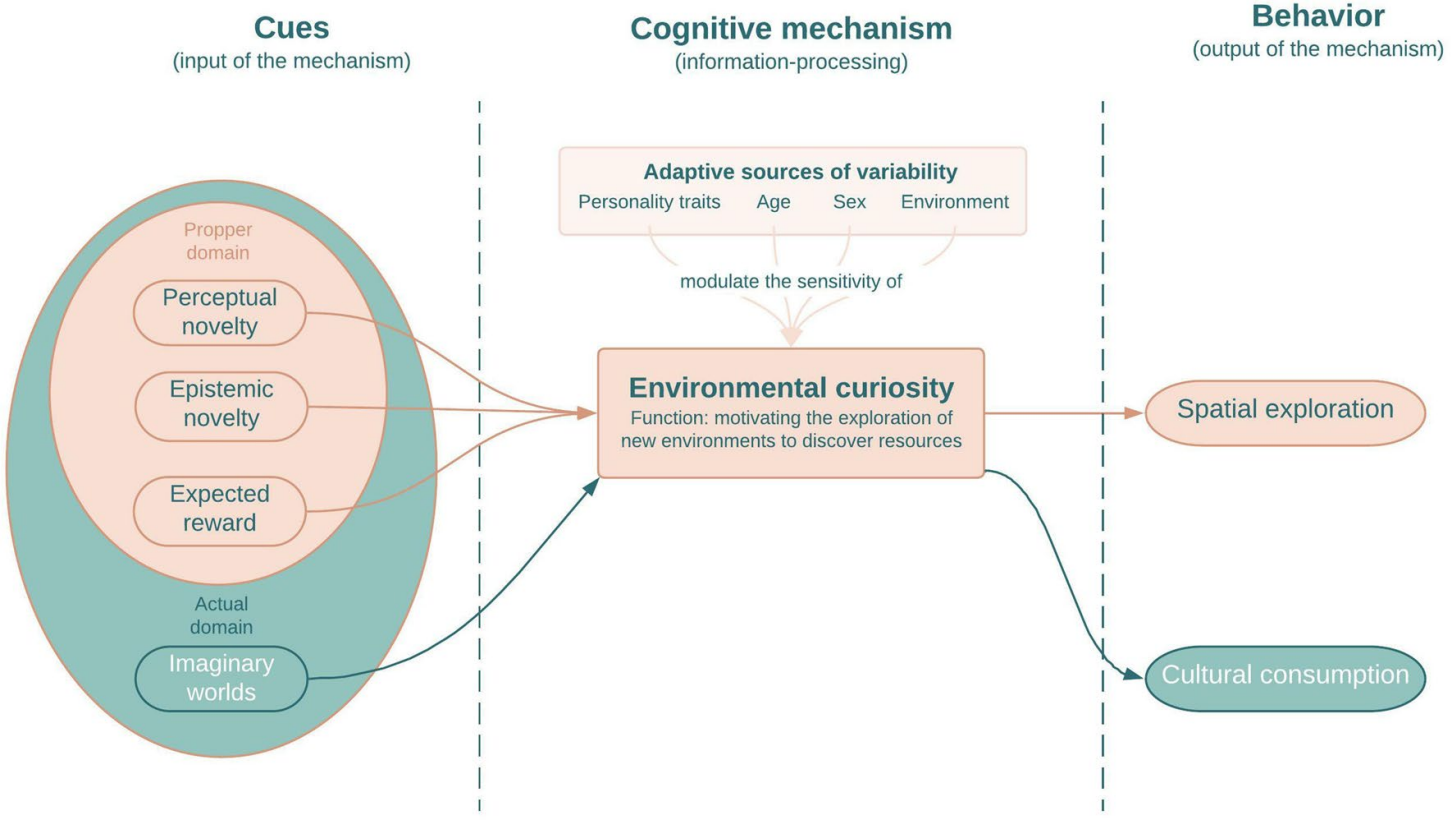
Proposed model of the computational architecture of environmental curiosity (in orange), with the actual domain of the cognitive mechanism (in green).

We reviewed evolutionary rationales and empirical evidence showing how the human motivation to explore (i.e., environmental curiosity) adaptively varies according to people’s personality traits, age, sex, and ecological conditions (Fig. 1).

Our hypothesis, therefore, leads to fine-grained predictions based on the adaptive variability of environmental curiosity^5, 20^. If imaginary worlds do exploit this cognitive mechanism, the sources of its adaptive flexibility that we reviewed should account for the variability in the human fascination for imaginary worlds, across time and populations.

## Results

### Study 1: Unsupervised clustering of movies

**Figure 2:**
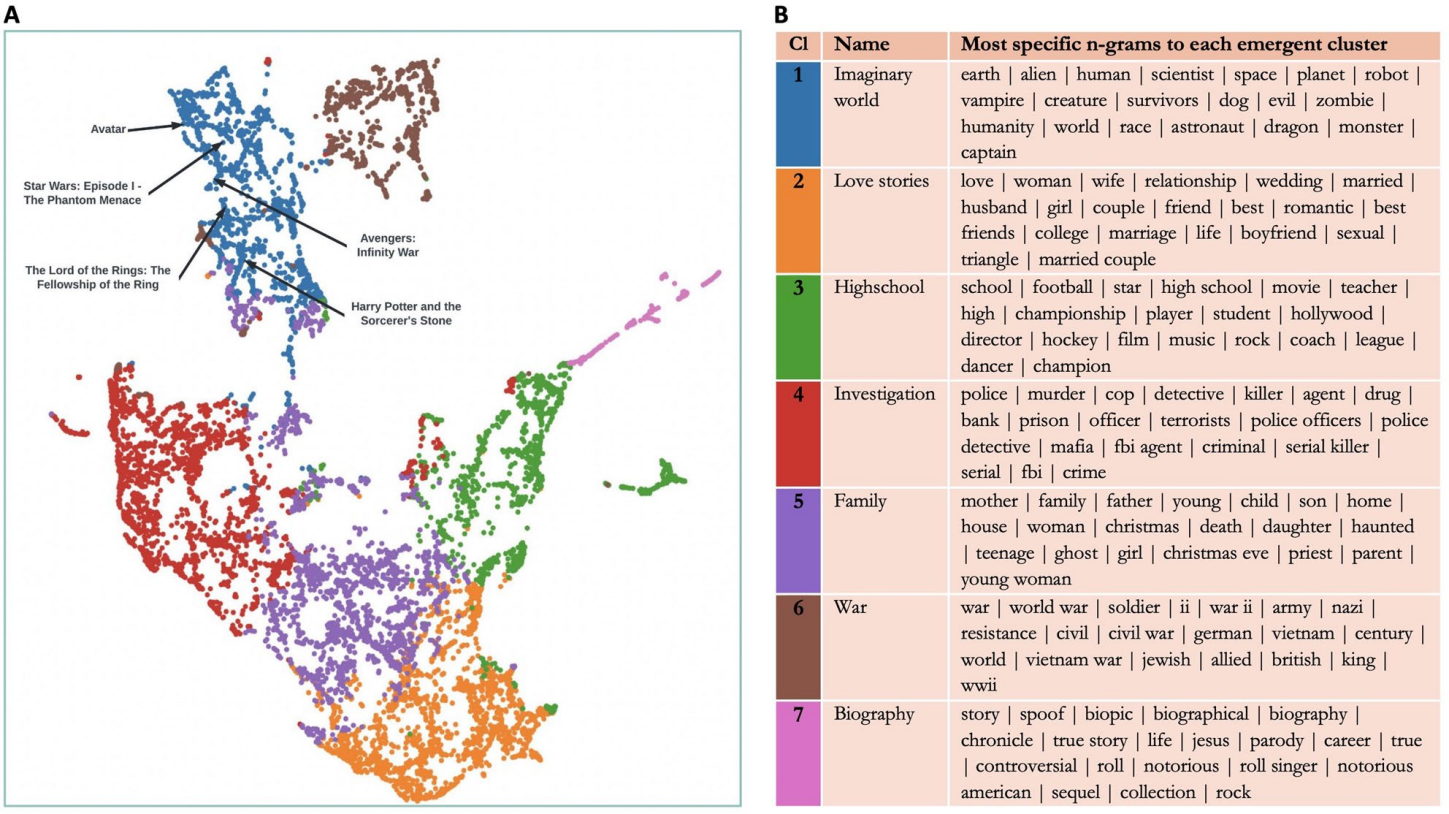
Unsupervised clustering of 9424 fictional movies. (**A**) Projection of movies in a semantic latent space, with 7 clusters, and the tagging of some movies from the imaginary-world cluster (cluster 1). (**B**) The 20 most specific n-grams for each cluster, and the names that we attributed to them based on the lists.

Before testing predictions about the sources of variability of the preference for imaginary worlds, we straightforwardly investigate whether fictional stories with imaginary worlds are related to exploration. We test that (1) stories with imaginary worlds constitute a well-identified cluster in the global set of movies produced and that (2) this emerging cluster is related to environmental curiosity through exploration-related content. We use independently two machine-learning algorithms. The random-forest algorithm, based on manually annotated movies, and trained on plot keywords, is designed to detect imaginary worlds in a sample of 9424 movies. This algorithm is successful in identifying movies set in imaginary worlds with an out-of-bag error rate of 9.35%. In parallel, we combine natural language processing techniques (i.e., Sbert Transformer) and topic modeling methods to project those 9424 movies into a semantic latent space, embedding the summary plots: the closer movie summaries are in their meaning and content, the closer movies will be into this space. Seven clusters naturally emerged, and we extracted the most specific n-grams to describe their content (see Fig. 2).

**Figure 3:**
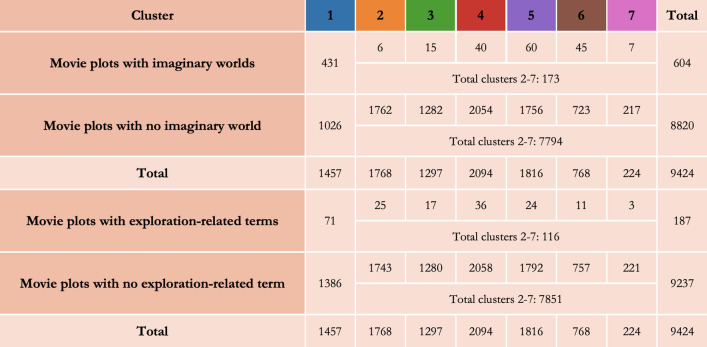
Contingency table of movies with and with no imaginary worlds, and with and with no exploration-related content, across cluster 1 and all other clusters.

Combining both algorithmic methods, we show that at least one cluster which has emerged embeds more specifically movies with imaginary worlds, as identified by the random-forest algorithm. First, we find a significant relationship between being associated with a specific cluster and being detected as a movie with an imaginary world (X^2^ (6, N = 9424) = 576.754, p < 0.001). We, therefore, reject the null hypothesis that asserts that the two variables are independent of each other: movies with imaginary worlds are not randomly distributed across all clusters (Fig. 3). We then perform the same analysis to show that one specific cluster (cluster 1, hereafter ‘imaginary world cluster’) specifically embeds movies with imaginary worlds (X^2^ (1, N = 9424) = 1542.759, p < 0.001). In fact, 71% of the movies with imaginary worlds detected by our algorithm belong to this specific cluster. Even if this cluster includes ‘only’ 30% of movies with imaginary worlds, this compares to only 2%, on average, in the other clusters. Let’s note that our algorithm is conservative: it does miss a lot of imaginary worlds (i.e., false negatives), but it is unlikely to wrongly label a movie that is not set in an imaginary world as having one (i.e., false positives; see “Supplementary Materials”). In all, movies with imaginary worlds are similar enough in their content for an unsupervised algorithm to cluster them together in one cluster, based only on plot summaries. This is also qualitatively observable in the n-grams that are most specific to this cluster, blending words related to multiple genres such as fantasy (e.g., ‘dragon’), science fiction (e.g., ‘alien’), dystopia (e.g., ‘survivor’), and more broadly related the supernatural (e.g., ‘vampire’).

Finally, we show that this imaginary-world cluster specifically embeds movies with exploration-related content, and significantly more so than any other cluster. Each movie summary is ascribed a binary variable of exploration-relatedness, based on the exact match between at least one word from an algorithmically generated list of exploration-related words and words from the movie summaries (see “Methods”). There is a significant relationship between being associated with a cluster and being associated with exploration-related content (X^2^(6, N = 9424) = 75.035, p < 0.001). We, therefore, reject the null hypothesis that asserts that the two variables are independent of each other: movies with exploration-related content are not randomly distributed among all clusters (Fig. 3). We perform again the same analysis and show that the imaginary-world cluster specifically embeds movies with exploration-related terms in their summary plots (X^2^(1, N = 9424) = 73.946, p < 0.001).

Consistent with our general hypothesis, these results suggest that fictions with imaginary worlds resemble each other, at least in part because they are related to exploration. Note that this study also comes as an external validity test for the random-forest algorithm: the latter is successful in identifying movies with imaginary worlds that, in addition, resemble each other in terms of their content. We will use its tagging of movies with imaginary worlds in the next study.

### Study 2: Demographic and psychological characteristics of individuals who ‘like’ movies with imaginary worlds on Facebook

**Figure 4:**
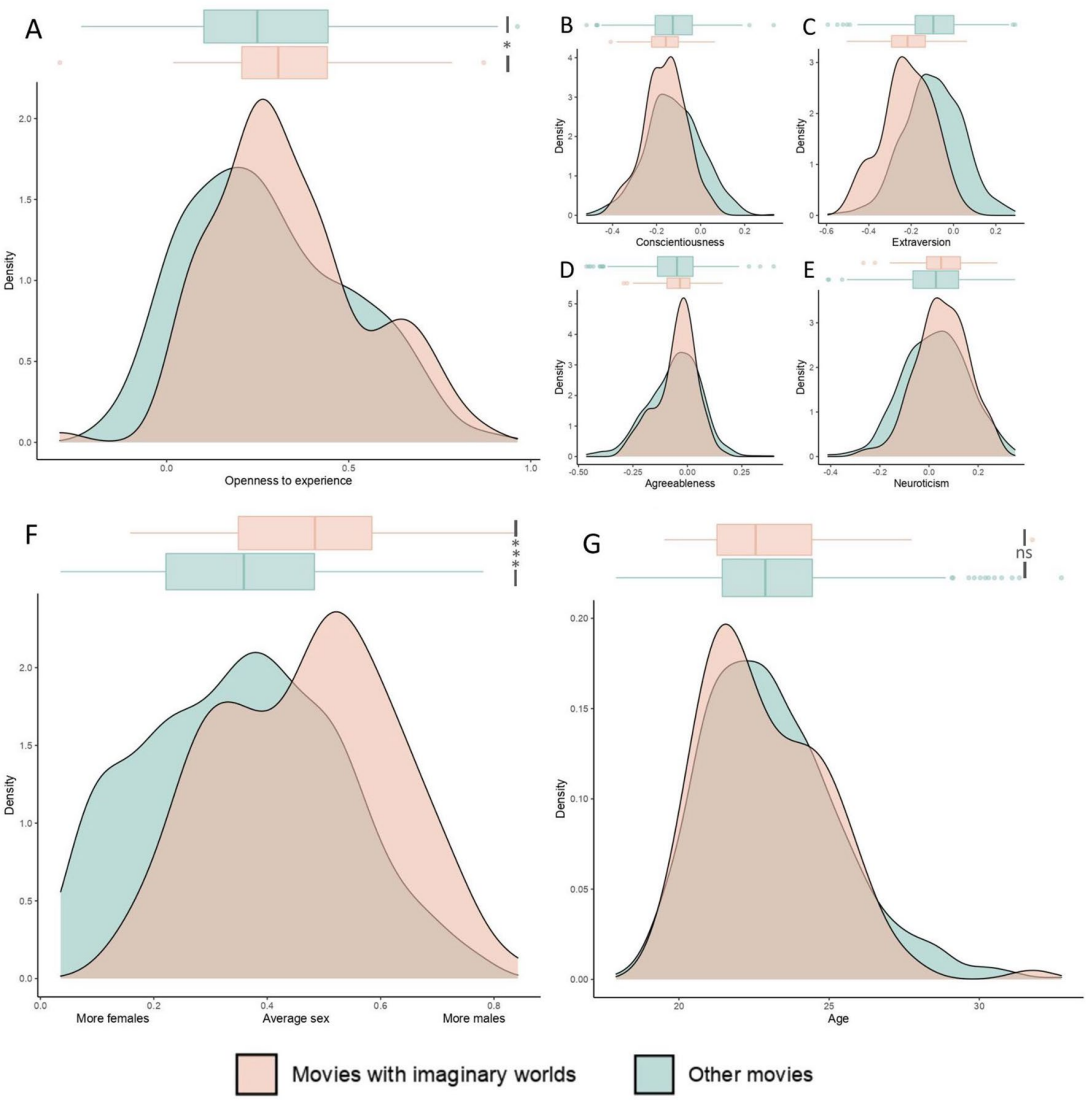
Distribution of the average scores of the Big Five personality traits (**A**–**E**), of the average sexes (**F**), and of the average ages (**G**) of movies with and with no imaginary worlds. *p < 0.05; **p < 0.01; ***p < 0.001.

**Figure 5:**
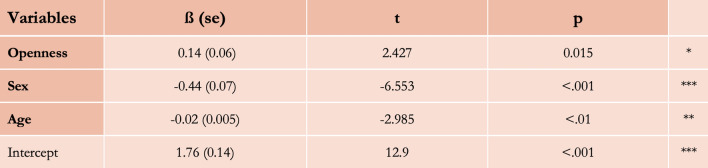
Model output of the Linear Probability Model explaining the presence of an imaginary world in a movie with 3 variables: the aggregated level of Openness-to-experience, sex, and age of the people who liked such movies on Facebook. *p < 0.05; **p < 0.01; ***p < 0.001.

We now turn to specific predictions about the variability of the fascination for imaginary worlds in stories, that we derived from the adaptive sources of variability of human environmental curiosity (see “Introduction”). We predicted that people higher in Openness-to-experience, younger people, males, and people living in affluent local environments would be more likely to enjoy fictional stories set in imaginary worlds. We used the Movie Personality Dataset (MPD) which aggregates averaged personality (i.e., Big Five) and demographic traits (i.e., sex, age) from the Facebook myPersonality Database (N = 3.5 million^81^). We couple this dataset with the outcome of the random-forest algorithm which efficiently identifies movies as being set in an imaginary world or not (see “Study 1”). First, we find that, as predicted, movies with imaginary worlds on Facebook are liked by an audience that is, on average, higher in Openness-to-experience than movies with no imaginary worlds (ß = 0.12, p < 0.01, CI [0.02, 0.22], Cohen’s *d* = 0.24; Fig. 4). In other words, approximately 60% of movies with imaginary worlds have higher aggregated scores of Openness-to-experience than the mean of Openness-to-experience of movies with no imaginary world. Although we had no specific prediction derived from our hypothesis, we report the correlations between the four other traits of the Big Five and the liking of imaginary worlds (Agreeableness: ß = 0.08, p = 0.451, CI [− 0.12, 0.27]; Conscientiousness: ß = − 0.29, p < 0.01, CI [− 0.47, − 0.10]; Extraversion, ß = − 0.68, p < 0.001, CI [− 0.84, − 0.54]; Neuroticism, ß = 0.19, p < 0.05, CI [0.01, 0.37]). Second, we found that movies with imaginary worlds on Facebook are liked by an audience that is, on average, more composed of males than movies with no imaginary worlds (ß = 0.44, p < 0.001, CI [0.31, 0.57], Cohen’s *d* = 0.68; Fig. 4). It means that there is a 68.5% chance that a movie with an imaginary world picked at random will have a higher percentage of males liking it on Facebook than a movie with no imaginary world picked at random. We found no significant association between the age of consumers and the presence of imaginary worlds in movies (ß = − 0.005, p = 0.33, CI [− 0.015, 0.0051], Fig. 4). Finally, with the full model, with all 3 variables of interest as explanatory variables, and the liking of movies with imaginary worlds as the outcome variable, we found significant coefficients with the predicted directions (Fig. 5).

With computational methods, we provide observational evidence that people who like movies with imaginary worlds on Facebook are overall higher in Openness-to-experience. These results are in line with existing empirical evidence showing an association between Openness-to-experience and the consumption of or preference for specific genres often associated with imaginary worlds such as science fiction and fantasy^81, 169–173^. Also consistent with our predictions, we provide evidence that people who report liking movies with imaginary worlds are more likely to be males. We did not find any significant association between age and the liking of movies with imaginary worlds. This can be explained by the very restricted range of Facebook users at the time the participants were interviewed (in 2009–2010): aggregated ages associated with each movie range from 17.9 to 32.7 years old. We would need a much larger range to assess the impact of age on the consumption of fictional stories with imaginary worlds. Let’s note that evolutionary developmental psychology does not make any strong predictions about the change in the sensibility of environmental curiosity within this specific life stage. Rather, it makes predictions about the difference in the sensitivity of environmental curiosity between this life stage, earlier ones, and older ones^174^. Further research should investigate the differences in cultural preferences between children and other life stages. Finally, let us note that, with this study, the results about the personality traits and biological sex of such audiences are generalizable only within the specific age range of this dataset.

### Study 3: Demographic and psychological characteristics of individuals who self-report liking stories set in imaginary worlds

**Figure 6:**
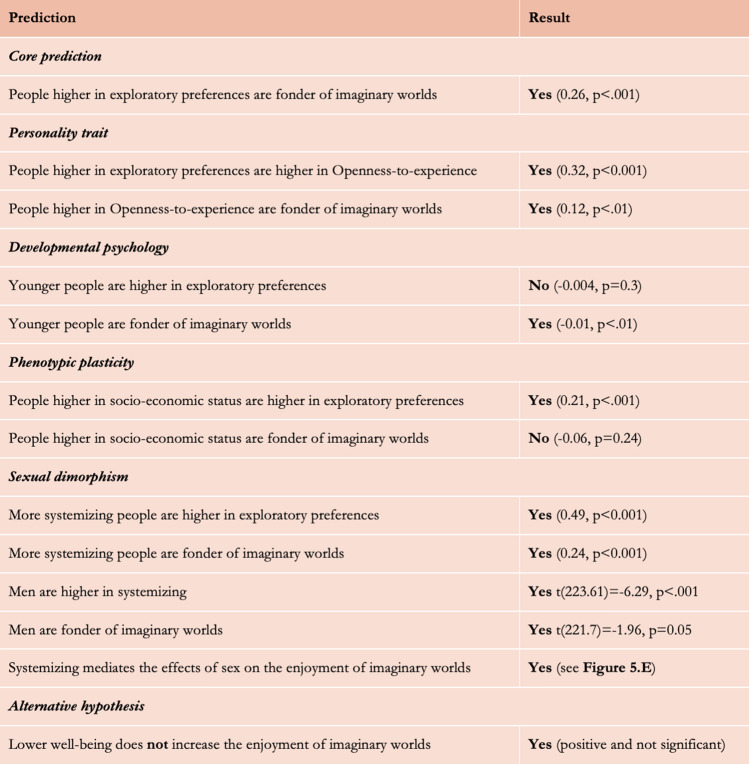
Summary of the predictions and results of the experimental study with the self-reporting paradigm, as pre-registered. We removed from the pre-registration 2 mediation tests that we could not perform (see Pre-registration).

We now turn to experimental tests of the same predictions (all pre-registered). We asked participants to report their preferences for fictional stories with imaginary worlds using a questionnaire and asked them to respond to a range of psychometric questionnaires (see “Methods”). We predicted that people higher in Openness-to-experience, younger people, males, and people living in affluent local environments would be more likely to enjoy stories set in imaginary worlds. We take advantage of experimental paradigms to further test two other hypotheses. We test the presumably complementary ‘systemizing hypothesis’, which suggests that people enjoy imaginary worlds because they like to understand the ways newly presented imaginary worlds are structured and operate (i.e., because they are ‘higher systemizers’). We also test the alternative and widely spread ‘escapist hypothesis’ which posits that people enjoy imaginary worlds because they want to escape the difficulties of the real world: we look at whether people who report having more difficulties in life also report enjoying more fictional stories with imaginary worlds. We predicted that it would not be the case. We report the results in Fig. 6.

**Figure 7:**
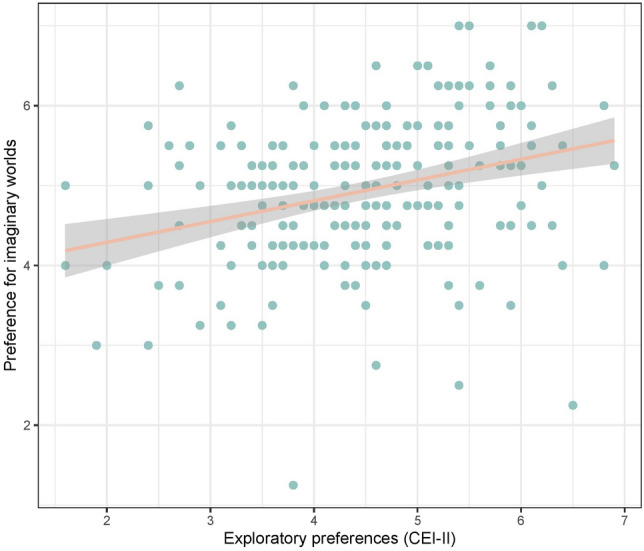
The scores of reported preferences for imaginary worlds as a function of CEI-II scores.

**Figure 8:**
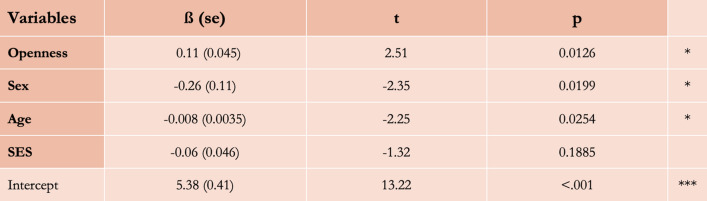
Model output of a Linear Model with the reported preference for imaginary worlds as the dependent variable and the level of Openness-to-experience, sex, age, and SES of the participants as the independent variables. *p < 0.05; **p < 0.01; ***p < 0.001.

First, we tested our core prediction about the association between the preference for imaginary worlds and environmental curiosity: people who score higher in the Curiosity and Exploration Inventory scale report enjoying more imaginary worlds in fictional stories (Fig. 7). Then, we tested predictions related to the adaptive variability of environmental curiosity and found that participants who report liking more imaginary worlds in stories are overall higher in Openness-to-experience, younger, and more likely to be males (Fig. 8). Although participants with higher socio-economic status scored significantly higher on the Curiosity and Exploration Inventory scale, they did not report enjoying more imaginary worlds. It is consistent with the hypothesis that phenotypic plasticity does impact exploratory preferences (which increase as the local ecology gets more affluent and predictable), but it suggests that it may not translate in the cultural domain. It might also be that this prediction has been affected by the reduction of the sample size due to participation exclusion.

**Figure 9:**
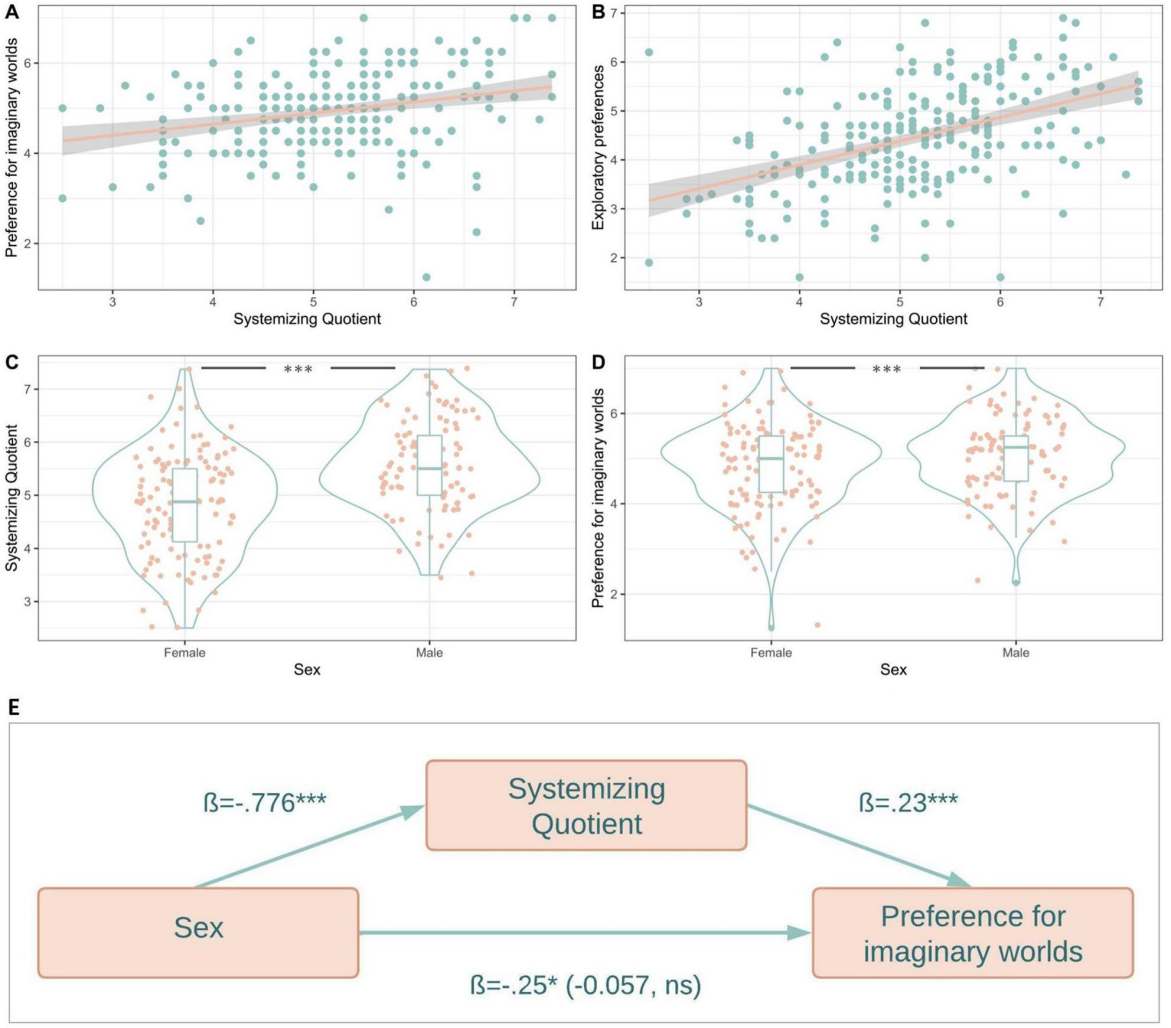
(**A**) Correlation between the preference for imaginary worlds and the Systemizing Quotient. (**B**) Correlation between exploratory preferences (CEI-II) and the Systemizing Quotient. (**C**) The association between sex and the scores of systemizing. (**D**) The association between sex and the reported preference for imaginary worlds. (**E**) Levels of systemizing mediate the effect of sex on the preference for imaginary worlds with an indirect effect of − 0.17 (p < 0.001), leaving a non-significant direct effect (in parentheses on the center path, next to the total effect). The proportion of effects mediated is 70%.

Let’s finally turn to the two other hypotheses that we tested. First, it does not seem that stories with imaginary worlds are enjoyed because they allow consumers to ‘escape’ the difficulties of the real world. We tested this prediction by looking at the correlation between a self-reported measure of well-being and the preference for imaginary worlds. We reasoned that, if this hypothesis were true, the more unhappy people are, the more they should like imaginary worlds. As predicted, this association turned out to be non-significant. Of course, more empirical tests should be run, but this first result suggests that the ‘escapist hypothesis’ is either false or incomplete. Finally, we tested the effect of levels of systemizing on the preference for imaginary worlds. This supplementary hypothesis was confirmed: the higher people are in systemizing, the higher they score on the Curiosity and Exploration Inventory scale and the more they report enjoying imaginary worlds. With a mediation analysis, we found that the systemizing quotient mediated 70% of the effect of sex on the preference for imaginary worlds: while this is not a causal paradigm, this result is consistent with the hypothesis that males enjoy more imaginary worlds in large part because they’re higher in systemizing (Fig. 9).

This study replicates previous findings of sex differences in systemizing, with similar magnitude^77, 78^. We further demonstrate that this difference translates in the cultural domain, impacting the reported preference for imaginary worlds in fiction. Besides, we show that the psychological trait of systemizing is highly correlated to exploratory preferences, supporting the hypothesis that systemizing is subsumed under the drive to explore that we coined environmental curiosity^175^. Under our theoretical account, systemizing is the labeling of an extreme form of information processing that this mechanism of curiosity about the physical world can take, which is indeed modulated by sex differences because of ancestral selection pressures. Crucially, this account may explain why fans of imaginary worlds like to explore imaginary worlds in depth rather than exploring new worlds again and again^176^, and why fans of imaginary worlds end up remembering and storing huge amounts of information related to the imaginary worlds they like, for instance in Wikipedia-like online ‘Fandoms’ (e.g., the Star Wars online fandom aggregates more than 175,000 pages^1^).

## Discussion

In all, we provide empirical evidence supporting the hypothesis that exploratory preferences explain why humans are fascinated by imaginary worlds in fictional stories. We reviewed the evolutionary psychological literature on environmental curiosity and exploratory preferences in humans. Then, we showed that fictions with imaginary worlds cluster together because of the semantic proximity of their summary plots, suggesting that they resemble each other in terms of their content. We showed that movies from this cluster are specifically associated with exploration-related content. As predicted by the exploration hypothesis^5^, we then provided evidence that the adaptive variability of the sensitivity of environmental curiosity reflects, and therefore likely explains, the variability of the preference for imaginary worlds in fiction.

Observational analyses of large cultural datasets showed that people who ‘like’ movies with imaginary worlds on Facebook are overall higher in Openness-to-experience, younger, and more likely to be males (when controlling for the two other variables). This dataset can be biased as it aggregates personality scores of people who decided to create a Facebook account and ‘like’ movies on Facebook. Therefore, we replicated such findings with experimental methods: we provided consistent evidence that participants who report enjoying imaginary worlds in movies, novels, and video games are overall higher on a scale of exploratory preferences, higher in Openness-to-experience, younger, higher in systemizing, and more likely to be males. We did not find an association between the socioeconomic status of the participants and their reported preference for imaginary worlds, although participants higher in socio-economic status scored significantly higher on the scale of exploratory preferences. The core prediction that, in synchrony, the socioeconomic level of people should impact the preference for fictional stories set in imaginary worlds, through a mediating effect of environmental curiosity, should be further tested with other datasets or experimental tests. Future research should also further study the causal impact of ecological conditions on the production of speculative fictional stories in diachrony, at the macro-level of societies.

Cognitive scientists have long argued that universal cognitive adaptations can explain the evolution, stabilization, and distribution of cultural traits^21, 177–179^. Here we demonstrate that the way such cognitive adaptations vary (between individuals, across ontogeny, and with changes in the local ecologies) can explain the *variable* appeal of such cultural traits to human cognition. More specifically in studying fictional stories, some researchers have focused on universal appeal for some content features, making some stories more popular than others (^180–183^, e.g., for romance^184–188^, e.g., for horror^189^). We contend that evolutionary psychology now provides predictions and powerful ways to interpret findings about the *differences* and *changes* in human cultural preferences (e.g., for romance^158, 159^, e.g., for horror^160^). Further research in fiction study could investigate the variability of many other preferences (and associated consummatory behavior) with such an evolutionary framework.

Behind the field of entertainment, the success of imaginary worlds in modern societies reveals important changes in individual preferences and personality traits. Why would people come to enjoy stories with imaginary worlds now, and not before? Because we have provided empirical evidence that the appeal for imaginary worlds relies on exploratory preferences, the *increasing success* of fiction with imaginary worlds may reflect *changes* in human exploratory preferences. We proposed that humans universally become more curious and explorative as they live in more affluent ecologies, notably because the evolutionary costs of curiosity decrease in such environments. This hypothesis did not lead to significant results when comparing people’s preferences for imaginary worlds at different socio-economic levels. However, it could mean that people process other cues than sheer income to assess how well-off they are (e.g., cues at the country level, such as unemployment insurance). If our hypothesis is true, economic growths of the last decades or even of the last centuries, in most human societies, likely fueled a bigger and bigger audience for stories set in imaginary worlds, and producers of fiction could therefore invest more and more in the creation and refinement of such worlds^190^.

It is worth noting that this hypothesis fits qualitative observations about the cultural evolution of imaginary worlds at the country level. Modern stories with imaginary worlds first became popular in the United Kingdom^4^, which was at the time the leading country in terms of GDP per capita^191^, and then mostly developed in the Euro-American sphere. By contrast, for most of the nineteenth and twentieth centuries, the popularity of imaginary worlds was rather limited in less economically developed countries. For instance, while Jules Verne was first translated into Chinese in the early 20th and inspired Chinese writers to write science-fiction and fantasy stories during the late Qing dynasty and early Republican era, stories set in imaginary worlds remained marginal in Chinese literature during the twentieth century^192, 193^. In East Asia, imaginary worlds started to become mainstream first in Japan in the 1950s^194, 195^ which had started its industrialization in the late nineteenth century, then in Hong Kong and Taiwan^196^, which had started to develop economically in the 1970’. During the same time, imaginary worlds were much less popular in mainland China^192, 197^ and they became mainstream in mainland China at the turn of the new millennium, that is, 20 years after the take-off of the Chinese economy^196–199^.

While future empirical research should thoroughly test this hypothesis, there are already several indications in favor of this idea. For instance, recent studies on the evolution of personality traits have shown an increase in Openness-to-experience in high-income countries both in Western^200^ and Eastern societies^201^. However, these studies are obviously limited, both in terms of sample size, population diversity, and measurement. If we are right, the rise of imaginary worlds in all parts of the world would suggest that Openness-to-experience is rising in modern societies and that it has been rising for at least 150 years. That is, we now could use the evolution of the relative production of stories with imaginary worlds as a proxy for changes in human exploratory preferences. Our results can therefore contribute to the understanding of behavioral and cultural changes over the long run^146, 166, 202^.

## Methods

### Data

#### Extraction of existing data from the Internet or previous studies (Studies 1 & 2)

We use the Internet Movie Database (IMDb) to obtain metadata about 9424 movies, such as their genres, summary plots, and their keywords. In the Movie Personality Dataset (MPD), for each of the 846 movies, we have (1) the movie metadata, (2) the average personality traits, average age, and average sex of people who like it on Facebook, and (3) the presence (or not) of an imaginary world in it (see “Algorithmic methods”, below). Nave et al.^81^ built an important dataset that makes it possible to map the associations between movie characteristics and the characteristics common to people who like such movies. Note that because socio-demographic scores are aggregated, the sex variable associated with each movie becomes a continuous variable between 0 and 1 (as a percentage of males who liked it, where 1 would mean that all people who liked this movie on Facebook self-reported themselves as men).

#### Collection of original data through experimental designs (Study 3)

The design and predictions for this study were pre-registered (https://osf.io/8yj3v). All methods were carried out in accordance with relevant regulations and approved by the Conseil d’évaluation éthique pour les recherches en santé, CERES n°201,659. We recruited 350 participants from the online research participation platform Prolific (180 males, 165 females, 4 others, *M*_*age*_ = 46, *SD*_*age*_ = 19.5). Participants confirmed their informed consent. We removed participants failing the attention check and participants failing to respond to the follow-up study, leaving a total sample of 230 participants (101 males, 127 females, 2 others, *M*_*age*_ = 48, *SD*_*age*_ = 16.3, *Range*_*age*_: 19–82; we still run the analyses that were possible without the follow-up study with the entire sample size, after removal of participants who failed the attention check; see “Supplementary Materials”). Our pre-registered sample size was higher (319) for 95% power (with α = 0.2 and p < 0.05), but with 230 participants the statistical power level is above 80%. All methods were carried out by relevant guidelines and regulations and informed consent was obtained from all subjects participants.

In the first part of the experimental study, 3 paradigms aimed at capturing an individual score of preference for fictional stories with imaginary worlds. Since the latter paradigm is the only one that provided consistent results with the large-scale observational study, we only provided detailed results for this one (see “Supplementary Information” for the results of the other paradigms). While we do not deny that such failures to find results consistent with our predictions across all pre-registered paradigms weakens the significance of our findings, it is not surprising to us that the self-reporting method (efficiently used in similar research; e.g.,^203, 204^) has overall more predictive power than newly designed paradigms. Besides, when asked to choose between two movies or rate a movie summary, people likely use lots of cues to make their choice, such that the fact that it takes place in an imaginary world might not be decisive. Conversely, the last paradigm, where participants are straightforwardly asked whether they like movies, novels, or video games set in imaginary worlds, targets more precisely the content feature we want to study.

We believe that such paradigms have limitations that can explain such results. Our theory predicts that some content features go along better together, because they tap into psychological preferences that share common cognitive and neural bases, and therefore are present in the same people. This would mean that some of the randomly created movie plots would be ‘objectively’ better at appealing to a certain audience because by chance they would bring together locations and plots that are psychologically ‘consistent’. Some others are not. This creates a bias in the randomly created plots. Regarding the second paradigm, it is obvious that people take many elements into account when deciding which of two films they would prefer to watch. The presence of imaginary worlds might not make any difference for some people and might even be hard to detect with the cues we present them with. We now think that all content features of movies should be controlled for in such paradigms if we want to be precise about what drives people to consume and enjoy some stories.

Let’s note, however, that the three computed scores of preferences for imaginary worlds all significantly and positively correlate with each other. It suggests that the (self-reported) specific preference for imaginary worlds drives the actual choice of consumption of movies, while not driving it enough to provide significant results with our sample size (see “Supplementary Materials”). Further research should keep on trying to find more ecologically valid experimental paradigms to complement findings from self-reporting. We believe that the associations between horror movies and morbid curiosity^204, 205^ and between movies with imaginary worlds and environmental curiosity could serve as tests that new experimental paradigms are successful in capturing the preferences of consumers, before expanding the methodology.

For the self-reporting paradigm, we first created an 8-item scale. A factor analysis (KMO sampling adequacy = 0.62; see “Supplementary Materials”) indicated that two clusters of items emerged from the responses (X^2^(13) = 60.19, p < 0.001). We removed the items that didn’t load onto factor 2, which was more specific to the preference for imaginary worlds (see “Supplementary Materials”). The 4-item scale showed near-acceptable reliability (α = 0.66). Here are the 4 items, that participants had to rate on a 7-Likert scale from ‘I fully disagree’ to ‘I fully agree’: (1) ‘I like movies, novels and video games with more information about the world than about the characters’, (2) ‘I like movies, novels and video games in which the fictional characters explore their environment’, (3) ‘I like movies, novels and video games with novel and surprising technologies’, and (4) ‘I like movies, novels and video games which make me feel I am traveling in a foreign world’. The final individual score of preference for imaginary worlds is the mean of all the ratings.

Then, the participants were also asked to respond to (1) the Big Five questionnaire BFI-10^206^, to measure the score of Openness-to-experience, (2) the Curiosity and Exploration Inventory-II^207^ to measure exploratory preferences, (3) the short Warwick-Edinburgh Mental Well-being Scale^208^ to assess well-being scores, (4) the 8-item version of the Systemizing Quotient^209^ to measure scores of systemizing, (5) the childhood and current Socio-Economic Status (as designed in^210^) as a proxy for the affluence of the local ecology, (6) their reported gender, and (7) their age.

### Algorithmic methods

#### Random-forest algorithm (study 1 & 2)

The first step is to detect the presence of imaginary worlds in movies. We start by manually coding 385 movies randomly selected in the IMDb dataset, as being set in an imaginary world or not. We base this decision on one main criterion: whether or not the IMDb movie summary mentions a location that does not exist in the real world. Then, we extend this categorization to 9424 movies with a classification algorithm based on a random-forest method^211^ and trained on plot keywords (i.e., user-generated keywords associated with movies which describe “any notable object, concept, style or action that takes place during a title”). This algorithm is successful in identifying movies set in imaginary worlds with an out-of-bag error rate of 9.35%. Among the 328 movies annotated as not being set in an imaginary world, the random-forest algorithm miscategorized only 5 of them, and among the 57 movies that we manually annotated as being set in an imaginary world, it accurately finds 26 of them: the algorithm, therefore, underestimates the number of movies with imaginary worlds. To further validate the external validity of this predictive algorithm, we showed that movies identified as being set in an imaginary world by the algorithm were more likely to be classified in the science fiction and fantasy IMDb genres, two genres in which producers of fiction commonly classify fictions with imaginary worlds (see “Supplementary Materials” for the results).

#### Topic modeling method (study 1)

Independently from this first step, we use Natural Language Processing methods and Topic Modeling to project those 9424 movies into a semantic latent space. More specifically, we use SBert Transformer^211–214^, which has been trained on millions of common language corpora and can map words, sentences, and paragraphs to a multidimensional dense vector space (i.e., word embedding;^215, 216^, and which achieves state-of-the-art performance on machine learning-tasks related to text understanding^217^). Such techniques allow us to define the semantic closeness of words, sentences, or paragraphs in an unsupervised fashion, by making the algorithm look at the contexts in which words are used in common language corpora. The underlying assumption is that words used in similar ways, at such a very large scale, have similar meanings. Here, we project movies into a semantic space using their movie description: the closer movie summaries are semantically, the closer movies will be into this space. Then, we use the K-Means algorithm to cluster this space into 7 clusters (with the elbow method^218^ determining the number of clusters that maximizes the explained variation). For every cluster, we compute the most 20 specific n-grams using the chi-squared statistics test, thus providing the words that most specifically describe the clusters. For these computations, we use the Python ‘bunkatech’ package (https://github.com/charlesdedampierre/BunkaTopics).

#### Creation of an extended list of exploration-related terms

First, we manually create a list of 5 core terms directly related to exploration (i.e., ‘exploration’, ‘explorer’, ‘explorers’, ‘explores’, ‘exploring’). Then, we extend this list using again the algorithm Sbert Transformer, this time applied to the movie summaries. More specifically, we find the 20 words that are closest to each of the core terms in the dataset of the movie summaries itself (with no consideration of whether the movie is set in an imaginary world or not, or is part of the imaginary-world cluster or not), and then remove duplicates. We end up with 37 terms in this extended list of exploration-related words.

### Statistical models

#### Chi-2 tests of independence (study 1)

We combine both algorithmic methods (see “Algorithmic methods”): we use the chi-squared test of independence to check that at least one cluster which has emerged from the Topic Modeling method embeds more specifically movies with imaginary worlds, as identified by the random-forest algorithm. In other words, we look at the correspondence between two computationally designed features of 9424 movies: belonging to an emergent cluster and being detected as a movie with an imaginary world. We use the same test to check that the imaginary-world cluster embeds more specifically such movies with exploration-related terms. In other words, we look at the correspondence between two computationally designed features of movies: belonging to the imaginary-world cluster and the binary variable of exploration-relatedness.

#### Linear probability models (study 2)

To test the correlations between the appeal for movies with imaginary worlds and the average scores of Openness-to-experience, age, and sex, we use Linear Probability Models, with such scores as explanatory variables, and the binary variable of the presence or absence of an imaginary world as the outcome variable. Then, we use one Linear Probability Model with all the scores as explanatory variables and the binary variable of the presence or absence of an imaginary world as the outcome variable (see “Supplementary Materials”, Appendix B, for model assumptions check).

#### Linear models and t-tests (study 3)

To test predictions with the data from the experiment, we use (1) linear models with the score of preference for imaginary worlds as the dependent variables, and, in turn, the score of the Curiosity and Exploration Inventory-II, the score of Openness-to-experience, the age, the socio-economic status, the Systemizing-Quotient, and the Well-Being score, (2) linear models with the score of Curiosity and Exploration Inventory-II as the dependent variables, and in turn, the score of Openness-to-experience, the age, the socio-economic status, and the Systemizing Quotient, (3) t-tests with, in turn, the score of preference for imaginary worlds and the Systemizing Quotient as the dependent variables, and the sex as a binary variable as the dependent variable. We also perform a mediation analysis with the R ‘Mediation’ package. Finally, we perform a non-preregistered linear model with the score of preference for imaginary worlds as the dependent variable and scores of Openness-to-experience, sex, age, and socio-economic status as the independent variables.

## Supplementary Information

Supplementary Information.

## Data Availability

Pre-registration, data, and R scripts are available on OSF: https://osf.io/zu2gq/?view_only=.

## References

[1] Besson A (2015). Constellations: des mondes fictionnels dans l’imaginaire contemporain.

[2] Ryan ML, Thon JN (2014). Storyworlds Across Media: Toward a Media-Conscious Narratology.

[3] Suvin D (1979). Metamorphoses of Science Fiction: On the Poetics and History of a Literary Genre.

[4] Wolf MJP (2013). Building Imaginary Worlds: The Theory and History of Subcreation.

[5] Dubourg E, Baumard N (2022). Why Imaginary World? The psychological foundations and cultural evolution of fictions with imaginary worlds. Behav. Brain Sci..

[6] Berlyne DE (1954). A theory of human curiosity. Br. J. Psychol. General Sect..

[7] Dubey R, Griffiths TL (2020). Reconciling novelty and complexity through a rational analysis of curiosity. Psychol. Rev..

[8] FitzGibbon L, Lau JKL, Murayama K (2020). The seductive lure of curiosity: Information as a motivationally salient reward. Curr. Opin. Behav. Sci..

[9] Gershman SJ (2018). Deconstructing the human algorithms for exploration. Cognition.

[10] Gottlieb J, Oudeyer PY, Lopes M, Baranes A (2013). Information-seeking, curiosity, and attention: Computational and neural mechanisms. Trends Cogn. Sci..

[11] Kidd C, Hayden BY (2015). The psychology and neuroscience of curiosity. Neuron.

[12] Oudeyer, P.Y., Gottlieb, J. & Lopes, M. Intrinsic motivation, curiosity, and learning. in *Progress in Brain Research [Internet]*. Elsevier; 2016 [cited 2020 Oct 14], 257–284. https://linkinghub.elsevier.com/retrieve/pii/S0079612316300589 10.1016/bs.pbr.2016.05.005 27926442

[13] Schulz E, Gershman SJ (2019). The algorithmic architecture of exploration in the human brain. Curr. Opin. Neurobiol..

[14] Wilson RC, Geana A, White JM, Ludvig EA, Cohen JD (2014). Humans use directed and random exploration to solve the explore–exploit dilemma. J. Exp. Psychol. Gen..

[15] Wilson, R.C., Wang, S., Sadeghiyeh, H., Cohen, J.D. Deep exploration as a unifying account of explore-exploit behavior [Internet]. PsyArXiv; 2020 Feb [cited 2022 May 10]. https://osf.io/uj85c

[16] Kaplan, S. Environmental preference in a knowledge-seeking, knowledge-using organism. in *The Adapted Mind: Evolutionary Psychology and the Generation of Culture*, 581–598. (Oxford University Press, 1992).

[17] Orians, G.H., Heerwagen, J.H. Evolved responses to landscapes. in *The Adapted Mind: Evolutionary Psychology and the Generation of Culture*, 555–579. (Oxford University Press, 1992).

[18] Raichlen DA, Wood BM, Gordon AD, Mabulla AZP, Marlowe FW, Pontzer H (2014). Evidence of Lévy walk foraging patterns in human hunter–gatherers. Proc. Natl. Acad. Sci. USA.

[19] Barrett D (2010). Supernormal Stimuli: How Primal Urges Overran Their Evolutionary Purpose.

[20] Dubourg E, Baumard N (2022). Why and how did narrative fictions evolve? Fictions as entertainment technologies. Front. Psychol..

[21] Sperber D, Hirschfeld LA (2004). The cognitive foundations of cultural stability and diversity. Trends Cogn. Sci..

[22] Osiurak F, Reynaud E (2019). The elephant in the room: What matters cognitively in cumulative technological culture. Behav. Brain Sci..

[23] Kashdan TB, Stiksma MC, Disabato DJ, McKnight PE, Bekier J, Kaji J (2018). The five-dimensional curiosity scale: Capturing the bandwidth of curiosity and identifying four unique subgroups of curious people. J. Res. Pers..

[24] Hills TT (2006). Animal foraging and the evolution of goal-directed cognition. Cogn. Sci..

[25] Hills TT, Todd PM, Goldstone RL (2010). The central executive as a search process: Priming exploration and exploitation across domains. J. Exp. Psychol. Gen..

[26] Hills TT, Todd PM, Lazer D, Redish AD, Couzin ID (2015). Exploration versus exploitation in space, mind, and society. Trends Cogn. Sci..

[27] Hills, T.T., Stroup, W. Cognitive exploration and search behavior in the development of endogenous representations. in *San Diego* (2004).

[28] Kaplan S, Nasar JL (1988). Perception and landscape: Conceptions and misconceptions. Environmental Aesthetics [Internet].

[29] Falk JH, Balling JD (2010). Evolutionary influence on human landscape preference. Environ. Behav..

[30] Petzke TM, Schomaker J (2022). A bias toward the unknown: individual and environmental factors influencing exploratory behavior. Ann. NY Acad. Sci..

[31] Poli F, Meyer M, Mars RB, Hunnius S (2022). Contributions of expected learning progress and perceptual novelty to curiosity-driven exploration. Cognition.

[32] Stojic H, Analytis PP, Schulz E, Speekenbrink M (2020). It’s new, but is it good? How generalization and uncertainty guide the exploration of novel options. J. Exp. Psychol. Gen..

[33] Bunzeck N, Düzel E (2006). Absolute coding of stimulus novelty in the human substantia Nigra/VTA. Neuron.

[34] Frank MJ, Doll BB, Oas-Terpstra J, Moreno F (2009). Prefrontal and striatal dopaminergic genes predict individual differences in exploration and exploitation. Nat. Neurosci..

[35] Kakade S, Dayan P (2002). Dopamine: Generalization and bonuses. Neural Netw..

[36] Knutson B, Cooper JC (2006). The lure of the unknown. Neuron.

[37] Koster, R., Seow, T.X., Dolan, R.J., Düzel, E. Stimulus novelty energizes actions in the absence of explicit reward. in (Verguts, T., ed) *PLoS ONE*. **11**(7), e0159120 (2016). 10.1371/journal.pone.0159120 PMC4944950 27415631

[38] Krebs RM, Schott BH, Schütze H, Düzel E (2009). The novelty exploration bonus and its attentional modulation. Neuropsychologia.

[39] Krueger PM, Wilson RC, Cohen JD (2017). Strategies for exploration in the domain of losses. Judgment Decis. Making..

[40] Sutton, R.S. Integrated architectures for learning, planning, and reacting based on approximating dynamic programming. in *Machine Learning Proceedings 1990 [Internet]*. Elsevier; 1990 [cited 2020 May 25], 216–224. https://linkinghub.elsevier.com/retrieve/pii/B9781558601413500304

[41] Balling JD, Falk JH (1982). Development of visual preference for natural environments. Environ. Behav..

[42] Herzog TR, Bryce AG (2007). Mystery and preference in within-forest settings. Environ. Behav..

[43] Kaplan R, Kaplan S, Brown T (1989). Environmental preference: A comparison of four domains of predictors. Environ. Behav..

[44] Cohen JD, McClure SM, Yu AJ (2007). Should I stay or should I go? How the human brain manages the trade-off between exploitation and exploration. Philos. Trans. R Soc. B..

[45] Wilson RC, Bonawitz E, Costa VD, Ebitz B (2020). Balancing exploration and exploitation with information and randomization. Curr Opin Behav Sci..

[46] Mehlhorn K, Newell BR, Todd PM, Lee MD, Morgan K, Braithwaite VA (2015). Unpacking the exploration–exploitation tradeoff: A synthesis of human and animal literatures. Decision.

[47] Healy K, Ezard THG, Jones OR, Salguero-Gómez R, Buckley YM (2019). Animal life history is shaped by the pace of life and the distribution of age-specific mortality and reproduction. Nat. Ecol. Evol..

[48] Healy S, Dekort S, Clayton N (2005). The hippocampus, spatial memory and food hoarding: a puzzle revisited. Trends Ecol. Evol..

[49] Rosati AG, Hare B (2012). Chimpanzees and bonobos exhibit divergent spatial memory development: Spatial memory development in chimpanzees and bonobos. Dev. Sci..

[50] Stevens JR, Rosati AG, Ross KR, Hauser MD (2005). Will travel for food: Spatial discounting in two new world monkeys. Curr. Biol..

[51] Verdolin JL (2006). Meta-analysis of foraging and predation risk trade-offs in terrestrial systems. Behav. Ecol. Sociobiol..

[52] Wolbers T, Hegarty M (2010). What determines our navigational abilities?. Trends Cogn. Sci..

[53] Buss DM (2009). How can evolutionary psychology successfully explain personality and individual differences?. Perspect. Psychol. Sci..

[54] de Vries RE, Tybur JM, Pollet TV, van Vugt M (2016). Evolution, situational affordances, and the HEXACO model of personality. Evol. Hum. Behav..

[55] Nettle D (2006). The evolution of personality variation in humans and other animals. Am. Psychol..

[56] DeYoung CG (2011). Sources of cognitive exploration: Genetic variation in the prefrontal dopamine system predicts Openness/Intellect. J. Res. Personal..

[57] McCrae RR, John OP (1992). An introduction to the five-factor model and its applications. J. Personal..

[58] Penke L, Jokela M (2016). The evolutionary genetics of personality revisited. Curr. Opin. Psychol..

[59] Dubois J, Eberhardt F, Paul LK, Adolphs R (2020). Personality beyond taxonomy. Nat. Hum. Behav..

[60] Nettle D, Penke L (2010). Personality: Bridging the literatures from human psychology and behavioural ecology. Philos. Trans. R Soc. B..

[61] Wright, A.J., Jackson, J.J. Are people consistently consistent in their personality? A longitudinal, person-centered test [Internet]. PsyArXiv; 2022. psyarxiv.com/8vt3j

[62] Durkee PK, Lukaszewski AW, von Rueden CR, Gurven MD, Buss DM, Tucker-Drob EM (2022). Niche diversity predicts personality structure across 115 nations. Psychol. Sci..

[63] Schmitt DP, Allik J, McCrae RR, Benet-Martínez V (2007). The geographic distribution of big five personality traits: Patterns and profiles of human self-description across 56 nations. J. Cross Cult. Psychol..

[64] Bainbridge TF, Ludeke SG, Smillie LD (2022). Evaluating the Big Five as an organizing framework for commonly used psychological trait scales. J. Pers. Soc. Psychol..

[65] Costa VD, Tran VL, Turchi J, Averbeck BB (2014). Dopamine modulates novelty seeking behavior during decision making. Behav. Neurosci..

[66] George JM, Zhou J (2001). When openness to experience and conscientiousness are related to creative behavior: An interactional approach. J. Appl. Psychol..

[67] Gocłowska MA, Ritter SM, Elliot AJ, Baas M (2019). Novelty seeking is linked to openness and extraversion, and can lead to greater creative performance. J. Pers..

[68] Li W, Li X, Huang L, Kong X, Yang W, Wei D (2015). Brain structure links trait creativity to openness to experience. Social Cognit. Affect. Neurosci..

[69] McCrae RR (1993). Openness to experience as a basic dimension of personality. Imagin. Cogn. Pers..

[70] Chen, Q., Christensen, A.P., Kenett, Y.N., Ren, Z., Condon, D.M., Bilder, R.M., *et al*. Mapping the creative personality: A psychometric network analysis of highly creative artists and scientists. *Creativity Res. J*. 1–16 (2023).

[71] Koutstaal W, Kedrick K, Gonzalez-Brito J (2022). Capturing, clarifying, and consolidating the curiosity-creativity connection. Sci. Rep..

[72] Carbone E, Meneghetti C, Borella E (2020). The role of personality in route learning in young and older adults. Personal. Individ. Differ..

[73] Cashdan E, Gaulin SJC (2016). Why go there? Evolution of mobility and spatial cognition in women and men: An introduction to the special issue. Hum. Nat..

[74] Condon DM, Wilt J, Cohen CA, Revelle W, Hegarty M, Uttal DH (2015). Sense of direction: General factor saturation and associations with the Big-Five traits. Personal. Individ. Differ..

[75] Davis HE, Cashdan E (2019). Spatial cognition, navigation, and mobility among children in a forager-horticulturalist population, the Tsimané of Bolivia. Cogn. Dev..

[76] Meneghetti C, Grimaldi F, Nucci M, Pazzaglia F (2020). Positive and negative wayfinding inclinations, choice of navigation aids, and how they relate to personality traits. J. Individ. Differ..

[77] Baron-Cohen S (2003). The Essential Difference: The Truth About the Male and Female Brain.

[78] Nettle D (2007). Empathizing and systemizing: What are they, and what do they contribute to our understanding of psychological sex differences?. Br. J. Psychol..

[79] Rawlings BS, Flynn EG, Kendal RL (2022). Personality predicts innovation and social learning in children: Implications for cultural evolution. Develop. Sci. [Internet]..

[80] Nettle D (2007). Personality: What Makes You the Way You Are.

[81] Nave, G., Rentfrow, J., Bhatia, S. We are what we watch: Movie plots predict the personalities of those who “like” them [Internet]. PsyArXiv; 2020 Nov [cited 2020 Nov 26]. https://osf.io/wsdu8

[82] Rawlings D (2003). Personality correlates of liking for ‘unpleasant’ paintings and photographs. Personal. Individ. Differ..

[83] Dollinger SJ (1993). Research note: Personality and music preference: extraversion and excitement seeking or openness to experience?. Psychol. Music.

[84] Rawlings D, Barrantes I Vidal N, Furnham A (2000). Personality and aesthetic preference in Spain and England: Two studies relating sensation seeking and openness to experience to liking for paintings and music. Eur. J. Pers..

[85] Vella EJ, Mills G (2017). Personality, uses of music, and music preference: The influence of openness to experience and extraversion. Psychol. Music.

[86] Schwaba T, Luhmann M, Denissen JJA, Chung JM, Bleidorn W (2018). Openness to experience and culture-openness transactions across the lifespan. J. Pers. Soc. Psychol..

[87] Rajagopal L, Hamouz FL (2009). Use of food attitudes and behaviors in determination of the personality characteristic of openness: A pilot study. Int. J. Intercult. Relat..

[88] Blanco NJ, Sloutsky VM (2020). Attentional mechanisms drive systematic exploration in young children. Cognition.

[89] Blanco NJ, Sloutsky VM (2021). Systematic exploration and uncertainty dominate young children’s choices. Dev. Sci. [Internet]..

[90] Loewenstein G (1994). The psychology of curiosity: A review and reinterpretation. Psychol. Bull..

[91] Del Giudice M (2014). Middle childhood: An evolutionary-developmental synthesis. Child Dev. Perspect..

[92] Gangestad SW, Kaplan H, Buss DM, Giudice MD (2015). Life history theory and evolutionary psychology. The Handbook of Evolutionary Psychology [Internet].

[93] Buchsbaum D, Bridgers S, Skolnick Weisberg D, Gopnik A (2012). The power of possibility: Causal learning, counterfactual reasoning, and pretend play. Philos. Trans. R Soc. B..

[94] Gopnik A (1803). Childhood as a solution to explore–exploit tensions. Philos. Trans. R Soc. B..

[95] Kaplan H, Hill K, Lancaster J, Hurtado AM (2000). A theory of human life history evolution: Diet, intelligence, and longevity. Evol Anthropol Issues News Rev..

[96] Gualtieri S, Finn AS (2022). The sweet spot: When children’s developing abilities, brains, and knowledge make them better learners than adults. Perspect. Psychol. Sci..

[97] Mata R, Wilke A, Czienskowski U (2009). Cognitive aging and adaptive foraging behavior. J. Gerontol. B Psychol. Sci. Soc. Sci..

[98] Mata R, Wilke A, Czienskowski U (2013). Foraging across the life span: Is there a reduction in exploration with aging?. Front. Neurosci. [Internet]..

[99] Sumner, E. *et al.* The exploration advantage. PsyArXiv (2019).

[100] Sumner, E., Steyvers, M. & Sarnecka, B.W. It’s not the treasure, it’s the hunt: Children are more explorative on an explore/exploit task than adults. In *Proceedings of the 41st annual conference of the cognitive science society* (Eds. A.K. Goel, C.M. Seifert, C. Freksa), pp. 2891–2897, Cognitive Science Society (2019).

[101] Liquin EG, Lombrozo T (2020). A functional approach to explanation-seeking curiosity. Cogn. Psychol..

[102] Liquin EG, Lombrozo T (2020). Explanation-seeking curiosity in childhood. Curr. Opin. Behav. Sci..

[103] Chin J, Anderson E, Chin CL, Fu WT (2015). Age differences in information search: An exploration-exploitation tradeoff model. Proc. Hum. Factors Ergon. Soc. Annu. Meet..

[104] Blanco, N.J. & Sloutsky, V. Systematic exploration and uncertainty dominate young children’s choices [internet]. PsyArXiv; 2019 Aug [cited 2021 Jun 9]. https://osf.io/72sfx 10.1111/desc.13026 PMC7867663 32767496

[105] Blanco NJ, Sloutsky VM (2019). Adaptive flexibility in category learning? Young children exhibit smaller costs of selective attention than adults. Dev. Psychol..

[106] Defeyter MA, German TP (2003). Acquiring an understanding of design: Evidence from children’s insight problem solving. Cognition.

[107] Gopnik A, O’Grady S, Lucas CG, Griffiths TL, Wente A, Bridgers S (2017). Changes in cognitive flexibility and hypothesis search across human life history from childhood to adolescence to adulthood. Proc. Natl. Acad. Sci. USA.

[108] Lucas CG, Bridgers S, Griffiths TL, Gopnik A (2014). When children are better (or at least more open-minded) learners than adults: Developmental differences in learning the forms of causal relationships. Cognition.

[109] Plebanek DJ, Sloutsky VM (2017). Costs of selective attention: When children notice what adults miss. Psychol. Sci..

[110] Schulz E, Wu CM, Ruggeri A, Meder B (2019). Searching for rewards like a child means less generalization and more directed exploration. Psychol. Sci..

[111] Chu L, Tsai JL, Fung HH (2021). Association between age and intellectual curiosity: The mediating roles of future time perspective and importance of curiosity. Eur. J. Ageing..

[112] Lloyd A, McKay R, Sebastian CL, Balsters JH (2021). Are adolescents more optimal decision-makers in novel environments? Examining the benefits of heightened exploration in a patch foraging paradigm. Dev. Sci..

[113] Steinberg L, Icenogle G, Shulman EP, Breiner K, Chein J, Bacchini D (2018). Around the world, adolescence is a time of heightened sensation seeking and immature self-regulation. Dev. Sci..

[114] Do KT, Sharp PB, Telzer EH (2020). Modernizing conceptions of valuation and cognitive-control deployment in adolescent risk taking. Curr. Dir. Psychol. Sci..

[115] Casey BJ, Jones RM, Hare TA (2008). The adolescent brain. Ann. N. Y. Acad. Sci..

[116] Duell N, Steinberg L (2019). Positive risk taking in adolescence. Child Dev. Perspect..

[117] Murty VP, Calabro F, Luna B (2016). The role of experience in adolescent cognitive development: Integration of executive, memory, and mesolimbic systems. Neurosci Biobehav Rev..

[118] Costa, P.T., McCrae, J.R.R., Martin, T.A., Oryol, V.E., Senin, I.G., Rukavishnikov, A.A., *et al*. Personality development from adolescence through adulthood: Further cross-cultural comparisons of age differences. in *Temperament and Personality Development Across the Life Span*. (Psychology Press, 2000).

[119] Donnellan MB, Lucas RE (2008). Age differences in the big five across the life span: Evidence from two national samples. Psychol. Aging..

[120] Labouvie-Vief G, Diehl M, Tarnowski A, Shen J (2000). Age differences in adult personality: Findings from the United States and China. J. Gerontol. Series B..

[121] Bleidorn W, Hopwood CJ, Back MD, Denissen JJA, Hennecke M, Hill PL (2021). Personality trait stability and change. Personal Sci..

[122] Bleidorn W, Hopwood CJ, Back MD, Denissen JJA, Hennecke M, Jokela M (2020). Longitudinal experience-wide association studies—A framework for studying personality change. Eur. J. Pers..

[123] Geary D (1998). Sexual selection, the division of labor, and the evolution of sex differences. Behav. Brain Sci..

[124] Ecuyer-Dab I, Robert M (2004). Have sex differences in spatial ability evolved from male competition for mating and female concern for survival?. Cognition.

[125] Gaulin SJC, Fitzgerald RW (1989). Sexual selection for spatial-learning ability. Anim. Behav..

[126] Greenwood PJ (1980). Mating systems, philopatry and dispersal in birds and mammals. Anim. Behav..

[127] Miner EJ, Gurven M, Kaplan H, Gaulin SJC (2014). Sex difference in travel is concentrated in adolescence and tracks reproductive interests. Proc. R Soc. B..

[128] Silverman I, Choi J, Mackewn A, Fisher M, Moro J, Olshansky E (2000). Evolved mechanisms underlying wayfinding: further studies on the hunter-gatherer theory of spatial sex differences. Evolut. Hum. Behav..

[129] Silverman, I., Eals, M. Sex differences in spatial abilities: Evolutionary theory and data. in *The Adapted Mind: Evolutionary Psychology and the Generation of Culture*, 533–549. (Oxford University Press, 1992).

[130] Linn MC, Petersen AC (1985). Emergence and characterization of sex differences in spatial ability: A meta-analysis. Child Dev..

[131] Voyer D, Voyer S, Bryden MP (1995). Magnitude of sex differences in spatial abilities: A meta-analysis and consideration of critical variables. Psychol. Bull..

[132] Lauer JE, Yhang E, Lourenco SF (2019). The development of gender differences in spatial reasoning: A meta-analytic review. Psychol. Bull..

[133] Ecuyer-Dab I, Robert M (2004). Spatial ability and home-range size: Examining the relationship in western men and women (*Homo sapiens*). J. Comp. Psychol..

[134] Matthews MH (1987). Gender, home range and environmental cognition. Trans. Inst. Br. Geogr..

[135] Clint EK, Sober E, Garland T, Rhodes JS (2012). Male superiority in spatial navigation: Adaptation or side effect?. Q. Rev. Biol..

[136] Charlton, B., Rosenkranz, P. Evolution of Empathizing and Systemizing: Empathizing as an aspect of social intelligence, systemizing as an evolutionarily later consequence of economic specialization. The Winnower [Internet]. 2016 Apr 29 [cited 2022 May 7]. https://thewinnower.com/papers/4249-evolution-of-empathizing-and-systemizing-empathizing-as-an-aspect-of-social-intelligence-systemizing-as-an-evolutionarily-later-consequence-of-economic-specialization

[137] Baron-Cohen S (2006). The hyper-systemizing, assortative mating theory of autism. Prog. Neuropsychopharmacol. Biol. Psychiatry.

[138] Greenberg DM, Warrier V, Allison C, Baron-Cohen S (2018). Testing the Empathizing-Systemizing theory of sex differences and the Extreme Male Brain theory of autism in half a million people. Proc. Natl. Acad. Sci. USA.

[139] Knickmeyer R, Baron-Cohen S, Raggatt P, Taylor K (2005). Foetal testosterone, social relationships, and restricted interests in children. J. Child. Psychol. Psychiat..

[140] Baron-Cohen S, Bolton P, Wheelwright S, Scahill V, Short L, Mead G (1998). Autism occurs more often in families of physicists, engineers, and mathematicians. Autism.

[141] Baron-Cohen S, Wheelwright S, Skinner R, Martin J, Clubley E (2001). The Autism-Spectrum Quotient (AQ): Evidence from asperger syndrome/high-functioning autism, males and females, scientists and mathematicians. J. Autism Dev. Disord..

[142] Byrd-Craven J, Massey AR, Calvi JL, Geary D (2015). Is systemizing a feature of the extreme male brain from an evolutionary perspective?. Personal. Individ. Differ..

[143] Kajonius PJ, Johnson J (2018). Sex differences in 30 facets of the five factor model of personality in the large public (N = 320,128). Personal. Individ. Differ..

[144] Wood, B.M., Harris, J.A., Raichlen, D.A., Pontzer, H., Sayre, K., Sancilio, A, *et al*. Gendered movement ecology and landscape use in Hadza hunter-gatherers. Nat Hum Behav [Internet]. 2021 Jan 4 [cited 2021 Feb 3]. http://www.nature.com/articles/s41562-020-01002-7 10.1038/s41562-020-01002-7 PMC8060163 33398143

[145] Cashdan E, Marlowe FW, Crittenden A, Porter C, Wood BM (2012). Sex differences in spatial cognition among Hadza foragers. Evol. Hum. Behav..

[146] Baumard N (2019). Psychological origins of the Industrial Revolution. Behav. Brain Sci..

[147] English S, Fawcett TW, Higginson AD, Trimmer PC, Uller T (2016). Adaptive use of information during growth can explain long-term effects of early life experiences. Am. Nat..

[148] Humphreys KL, Lee SS, Telzer EH, Gabard-Durnam LJ, Goff B, Flannery J (2015). Exploration-exploitation strategy is dependent on early experience: Exploration-Exploitation. Dev. Psychobiol..

[149] Mell H, Baumard N, André JB (2021). Time is money. Waiting costs explain why selection favors steeper time discounting in deprived environments. Evolut. Hum. Behav..

[150] Boon-Falleur M, Baumard N, André JB (2021). Risk-seeking or impatient? Disentangling variance and time in hazardous behaviors. Evol. Hum. Behav..

[151] Singh, M., Glowacki, L. Human social organization during the Late Pleistocene: Beyond the nomadic-egalitarian model [Internet]. EcoEvoRxiv; 2021 Mar [cited 2021 Mar 19]. https://osf.io/vusye

[152] Sadeghiyeh H, Wang S, Alberhasky MR, Kyllo HM, Shenhav A, Wilson RC (2020). Temporal discounting correlates with directed exploration but not with random exploration. Sci. Rep..

[153] Reyna-Hurtado, R., Teichroeb, J.A., Bonnell, T.R., Hernández-Sarabia, R.U., Vickers, S.M., Serio-Silva, J.C., *et al*. Primates adjust movement strategies due to changing food availability. in (Stephens, D. ed.) *Behavioral Ecology*. **29**(2), 368–76 (2018).

[154] Damerius LA, Graber SM, Willems EP, van Schaik CP (2017). Curiosity boosts orang-utan problem-solving ability. Anim. Behav..

[155] Forss S, Schuppli C, Haiden D, Zweifel N, Schaik C (2015). Contrasting responses to novelty by wild and captive orangutans. Am. J. Primatol..

[156] van Schaik CP, Burkart J, Damerius L, Forss SIF, Koops K, van Noordwijk MA (2016). The reluctant innovator: Orangutans and the phylogeny of creativity. Philos. Trans. R Soc. B..

[157] Katz K, Naug D (2015). Energetic state regulates the exploration–exploitation trade-off in honeybees. BEHECO..

[158] Mettke-Hofmann C, Winkler H, Leisler B (2002). The significance of ecological factors for exploration and neophobia in parrots. Ethology.

[159] Rojas-Ferrer, I., Thompson, M.J., Morand‐Ferron, J. Is exploration a metric for information gathering? Attraction to novelty and plasticity in black‐capped chickadees. in (Wright, J., ed) *Ethology*. **126**(4), 383–392 (2020).

[160] de Courson, B., Baumard, N.. Quantifying the Scientific Revolution [Internet]. SocArXiv; 2019 Dec [cited 2020 Oct 24]. https://osf.io/9ex8q

[161] Menardo E, Balboni G, Cubelli R (2017). Environmental factors and teenagers’ personalities: The role of personal and familial Socio-Cultural Level. Behav. Brain Res..

[162] Zhang D, Zhou Z, Gu C, Lei Y, Fan C (2018). Family socio-economic status and parent-child relationships are associated with the social creativity of elementary school children: The mediating role of personality traits. J. Child. Fam. Stud..

[163] Oh VY, Ismail I, Tong EM (2022). Income moderates changes in big-five personality traits across eighteen years. Eur. J. Pers..

[164] Lloyd A, McKay RT, Furl N (2022). Individuals with adverse childhood experiences explore less and underweight reward feedback. Proc. Natl. Acad. Sci. USA.

[165] Inglehart R (2020). Modernization and Postmodernization: Cultural, Economic, and Political Change in 43 Societies [Internet].

[166] Inglehart, R.F., Ponarin, E., Inglehart, R.C. Cultural change, slow and fast: The distinctive trajectory of norms governing gender equality and sexual orientation. *Social Forces*. sf;sox008v1 (2017).

[167] Korotayev, A., Zinkina, J., Slinko, E., Meshcherina, K. Human Values and Modernization: A Global Analysis. jogs [Internet]. 2019 [cited 2022 May 4];1(10). https://www.sociostudies.org/journal/articles/2189446/

[168] Wente A, Gopnik A, Fernández Flecha M, Garcia T, Buchsbaum D (1866). Causal learning, counterfactual reasoning and pretend play: A cross-cultural comparison of Peruvian, mixed- and low-socioeconomic status US children. Philos. Trans. R Soc. B..

[169] Annalyn N, Bos MW, Sigal L, Li B (2020). Predicting personality from book preferences with user-generated content labels. IEEE Trans. Affective Comput..

[170] Cantador, I., Fernández-Tobías, I. & Bellogín, A. Relating personality types with user preferences in multiple entertainment domains. In *CEUR Workshop Proceedings* 997. (2013).

[171] C. Olivia. Assessing the impact of gender and personality on film preferences. myPersonality project. 2010;Cambridge.

[172] Fong K, Mullin JB, Mar RA (2013). What you read matters: The role of fiction genre in predicting interpersonal sensitivity. Psychol. Aesthet. Creat. Arts.

[173] Kraaykamp G, van Eijck K (2005). Personality, media preferences, and cultural participation. Personality Individ. Differ..

[174] Del Giudice M, Angeleri R, Manera V (2009). The juvenile transition: A developmental switch point in human life history. Dev. Rev..

[175] Dubourg E, Baumard N (2022). Imaginary worlds through the evolutionary lens: Ultimate functions, proximate mechanisms, cultural distribution. Behav. Brain Sci..

[176] Browning, H., Veit, W. Autism and the preference for imaginary worlds. Behavioral and Brain Sciences. 2022; Commentary to Dubourg&Baumard, “Why Imaginary Worlds?” 10.1017/S0140525X21002211 36396420

[177] Boyer P (1998). Cognitive tracks of cultural inheritance: How evolved intuitive ontology governs cultural transmission. Am. Anthropol..

[178] Claidière N, Sperber D (2007). The role of attraction in cultural evolution. J. Cogn. Cult..

[179] Sperber D (1996). Explaining Culture: A Naturalistic Approach.

[180] Bloom P (2010). How Pleasure Works: The New Science of Why We Like What We Like.

[181] Saad G (2012). Nothing in popular culture makes sense except in the light of evolution. Rev. Gen. Psychol..

[182] Gottschall J (2012). The Storytelling Animal: How Stories Make Us Human.

[183] Nettle D (2005). The wheel of fire and the mating game: Explaining the origins of tragedy and comedy. J. Cult. Evol. Psychol..

[184] Alberti J (2013). “I Love You, Man”: Bromances, the construction of masculinity, and the continuing evolution of the romantic comedy. Quart. Rev. Film Video..

[185] Cox A, Fisher M (2009). The Texas billionaire’s pregnant bride: An evolutionary interpretation of romance fiction titles. J. Social Evolut. Cultural Psychol..

[186] Salmon C, Symons D (2004). Slash fiction and human mating psychology. J. Sex Res..

[187] Vanderbeke, D. On love and marriage in popular genres. in (Vanderbeke, D., Cooke, B., eds). Evolution and Popular Narrative [Internet]. Brill|Rodopi; 2019 [cited 2020 Feb 18]. https://brill.com/view/book/edcoll/9789004391161/BP000005.xml

[188] Martins, J.D., Baumard, N. Loving, fast and slow: A quantitative history of passion and tenderness in early Modern Europe. Submitted. (2022).

[189] Clasen M (2012). Monsters evolve: A biocultural approach to horror stories. Rev. Gen. Psychol..

[190] Lightner AD, Heckelsmiller C, Hagen EH (2022). Middle-earth wasn’t buit in a day: how do we explain the costs of creating a world? Commentary to ‘why imaginary worlds? The psychological foundations and cultural evolution of fictions with imaginary worlds’. Behav. Brain Sci..

[191] Manning, P. The Maddison Project: Historical GDP Estimates Worldwide. jwhi [Internet]. 2017 Sep 26 [cited 2021 Feb 1]. http://jwsr.pitt.edu/ojs/jwhi/article/view/821

[192] Jiang Q (2013). Translation and the development of science fiction in twentieth-century China. Sci.-Fiction Studies..

[193] Lu X (2000). A Brief History of Chinese Fiction.

[194] Bolton C, Csicsery-Ronay I, Tatsumi T (2007). Robot Ghosts and Wired Dreams: Japanese Science Fiction from Origins to Anime.

[195] Takayuki, T. Generations and controversies: An overview of Japanese science fiction, 1957–1997. *Sci. Fiction Stud*. **27** (2000).

[196] Rehling P (2012). Harry Potter, wuxia and the transcultural flow of fantasy texts in Taiwan. Inter-Asia Cultural Studies..

[197] Song, H. Chinese science fiction: A response to modernization. *Sci. Fiction Studies*. **40** (2013).

[198] Ni Z (2020). Xiuzhen (immortality cultivation) fantasy: Science, religion, and the novels of magic/superstition in contemporary China. Religions..

[199] Xu, S. Écritures de la fantasy dans la littérature sur Internet en Chine [Internet]. Translating Wor(l)ds. Università Ca’ Foscari Venezia, Italia; 2017 [cited 2021 Feb 11]. http://edizionicafoscari.unive.it/libri/978-88-6969-209-3/ecritures-de-la-fantasy-dans-la-litterature-sur-in/

[200] Stolarski M, Zajenkowski M, Meisenberg G (2013). National intelligence and personality: Their relationships and impact on national economic success. Intelligence.

[201] Peng L, Luo S (2021). Impact of social economic development on personality traits among Chinese college students: A cross-temporal meta-analysis, 2001–2016. Personality Individ. Differ..

[202] Baumard, N., Huillery, E., Zabro, L. The cultural evolution of love in history. *Nat. Hum. Behav*. (2022). 10.1038/s41562-022-01292-z 35256800

[203] Scrivner, C., Andersen, M.M., Schjødtuffe, C.M. The Psychological Benefits of Scary Play in Three Types of Horror Fans [Internet]. PsyArXiv; 2021 Jul [cited 2021 Jul 25]. https://osf.io/sdxe6

[204] Scrivner C, Johnson JA, Kjeldgaard-Christiansen J, Clasen M (2021). Pandemic practice: Horror fans and morbidly curious individuals are more psychologically resilient during the COVID-19 pandemic. Personality Individ. Differ..

[205] Scrivner, C. The psychology of morbid curiosity: Development and initial validation of the morbid curiosity scale. **52** (2021).

[206] Rammstedt B, John OP (2007). Measuring personality in one minute or less: A 10-item short version of the Big Five Inventory in English and German. J. Res. Pers..

[207] Kashdan TB, Gallagher MW, Silvia PJ, Winterstein BP, Breen WE, Terhar D (2009). The curiosity and exploration inventory-II: Development, factor structure, and psychometrics. J. Res. Pers..

[208] Stewart-Brown S, Tennant A, Tennant R, Platt S, Parkinson J, Weich S (2009). Internal construct validity of the Warwick-Edinburgh Mental Well-being Scale (WEMWBS): A Rasch analysis using data from the Scottish Health Education Population Survey. Health Qual. Life Outcomes..

[209] Veale JF, Williams MN (2017). The psychometric properties of a brief version of the systemizing quotient. Eur. J. Psychol. Assess..

[210] Griskevicius V, Tybur JM, Delton AW, Robertson TE (2011). The influence of mortality and socioeconomic status on risk and delayed rewards: A life history theory approach. J. Pers. Soc. Psychol..

[211] Breiman L (2001). Random forests. Mach. Learn..

[212] Devlin, J., Chang, M.W., Lee, K., Toutanova, K. BERT: Pre-training of deep bidirectional transformers for language understanding. 2018 [cited 2022 May 8]. https://arxiv.org/abs/1810.04805

[213] Liu, Y., Ott, M., Goyal, N., Du, J., Joshi, M., Chen, D., *et al*. RoBERTa: A robustly optimized BERT pretraining approach. arXiv:190711692 [cs] [Internet]. 2019 Jul 26 [cited 2022 May 8]. http://arxiv.org/abs/1907.11692

[214] Vaswani, A., Shazeer, N., Parmar, N., Uszkoreit, J, Jones, L., Gomez, A.N., *et al*. Attention is all you need. 2017 [cited 2022 May 8]. https://arxiv.org/abs/1706.03762

[215] Mikolov, T., Chen, K., Corrado, G., Dean, J. Efficient estimation of word representations in vector space. 2013 [cited 2022 May 8]. https://arxiv.org/abs/1301.3781

[216] Pennington, J., Socher, R., Manning, C. Glove: Global Vectors for Word Representation. in *Proceedings of the 2014 Conference on Empirical Methods in Natural Language Processing (EMNLP) [Internet]*. Doha, Qatar: Association for Computational Linguistics; 2014 [cited 2022 May 8]. p. 1532–43. http://aclweb.org/anthology/D14-1162

[217] Reimers, N., Gurevych, I. Making monolingual sentence embeddings multilingual using knowledge distillation. in *Proceedings of the 2020 Conference on Empirical Methods in Natural Language Processing (EMNLP) [Internet]*. Online: Association for Computational Linguistics; 2020 [cited 2022 May 8]. p. 4512–25. https://www.aclweb.org/anthology/2020.emnlp-main.365

[218] Thorndike RL (1953). Who belongs in the family?. Psychometrika.

