# Supplementary material for: Exploratory preferences explain the human fascination for imaginary worlds in fictional stories
Source: Sci Rep. 2023 May 28;13:8657. doi: 10.1038/s41598-023-35151-2 (PMC10225465; doi:10.1038/s41598-023-35151-2)
Supplement: Supplementary file 1 — Supplementary Information. [file 41598_2023_35151_MOESM1_ESM.docx]

**Supplementary Materials**

**Exploratory preferences explain the human**

**fascination for imaginary worlds in fiction**

Edgar Dubourg^1 a^, Valentin Thouzeau^1^, Charles de Dampierre^1^,

Andrei Mogoutov^1^, Nicolas Baumard^1^

^1^ Institut Jean Nicod (Paris), ENS, EHESS, CNRS, PSL

^a^ Corresponding author: [edgar.dubourg@gmail.com](mailto:edgar.dubourg@gmail.com)

**Table of content**

#

[**1. Random Forest Algorithm**](#_wcttw2hv1vq3) **2**

[1.1. Algorithm](#_dzcdhghcz840) 2

[1.2. Outcome](#_l6qrrjlw11qg) 2

[1.3. External validity test](#_bs01752dhtk1) 3

[**2. Topic Modeling**](#_3bs5t75g945g) **4**

[2.1. Algorithm](#_bwd1gw9gldz) 4

[2.2. Clustering](#_sxhprfou9omg) 4

[**3. Experiments**](#_ar09q5x9mpah) **5**

[3.1. Self-reporting paradigm](#_ckvzg0j95qla) 5

[3.2. Real Setting Preference: A control variable (non-pre-registered)](#_k5vas7bazwao) 7

[3.3. ‘Randomized plots’ paradigm](#_9xp78lqt4cg3) 7

[3.4. ‘Real movies’ paradigm](#_g6car98adsyh) 8

[3.5. Results](#_m1qpom165sfs) 9

[**Appendix A: Movies manually annotated**](#_52pvmz6ecnds) **12**

[**Appendix B: Checks of Model Assumptions for Experimental Study**](#_bhluv3ftnv3p) **17**

[Distribution of variables](#_e4lzqds52trh) 17

[Model assumptions](#_8ppvp4232v8y) 22

**Link to pre-registration and data**

<https://osf.io/8yj3v?revisionId=62309ee208800e00bf356ddf>

#

# 1. Random Forest Algorithm

## 1.1. Algorithm

First, two authors (E.D. and V.T.) manually annotated movies as being set in an imaginary world or not, based on one main criterion (i.e., a location doesn't exist in the real world; see Appendix A). Then, on these 385 films, we applied a random forest method (Breiman, 2001) in order to train a classification algorithm.

A random forest consists of a set of decision trees. The construction of an individual tree begins by randomly selecting a subset √(n) plot keywords, with n = 100 the total number of keyword plots retained according to their correlation, in absolute value, with the manually coded variable. Each tree is then built step by step. It selects the first keyword that best separates the dataset into two subsets and determines whether the presence or absence of this plot keyword is more often associated with an imaginary world or not. For each branch, it then selects the plot keyword that further subdivides the movies in that branch into two subsets and repeats this process until no plot keyword classifies the movies more precisely than the branch already does. The tree resulting from this algorithm thus decides, for each film evaluated, whether it is set in an imaginary world or not. The random sampling of 2000 different subsets of √(n) plot keywords allows to build 2000 decision trees. The consensus of the 2000 votes from the trees is used to determine the final category of the films. We verified graphically that 2000 trees were sufficient for the error rate to converge.

## 1.2. Outcome

|  |  | Estimated | |  |
| --- | --- | --- | --- | --- |
|  |  | Non-IW | IW | Class error |
| Manually annotated | Non-IW | 323 | 5 | 0.0152439 |
|  | IW | 31 | 26 | 0.5438596 |

**Table 2.** Confusion matrix of the random forest based on the 385 manually annotated movies, with 2000 trees and 10 variables tried at each split.

| iw | n |
| --- | --- |
| 0 | 8854 |
| 1 | 1457 |

**Table 3.** Numbers of movies with and with an imaginary world according to the algorithm.

## 1.3. External validity test

Finally, as an external validity test, we computed a Linear Probability Model with the binary variable Imaginary World as the independent variable, and the genres of the films as the explanatory variables. Films with imaginary worlds are generally found in the science fiction and fantasy genres, and to a lesser extent in the adventure genre. As predicted, the science fiction and fantasy genres significantly increased the probability that the film was set in an imaginary world (ß>0, p<.001). The family, action, animation, and adventure genres were also associated with films with imaginary worlds (ß>0, p<.001). It is worth noting that multiple genres are associated with each film, hence the correlation between these genres and the presence of imaginary worlds (e.g., Tomb Raider, from 2018 is categorized in the action, adventure, and fantasy genres). Interestingly, the romance, biography, comedy, crime, and drama genres were negatively correlated with our variable (ß<0, p<.001). The other genres showed no significant association with imaginary worlds. Overall, these results are consistent with our expectations and suggest that our algorithm is robust.

|  | Estimate | p |
| --- | --- | --- |
| genre_action | 0.245 | *** |
| genre_adventure | 0.40 | *** |
| genre_animation | 0.22 | *** |
| genre_biography | -0.14 | * |
| genre_comedy | -0.10 | *** |
| genre_crime | -0.03 | *** |
| genre_drama | -0.17 | *** |
| genre_family | 0.13 | *** |
| genre_fantasy | 0.15 | *** |
| genre_history | -0.06 |  |
| genre_horror | -0.004 |  |
| genre_music | -0.11 | . |
| genre_musical | 0.03 |  |
| genre_mystery | -0.048 |  |
| genre_romance | -0.13 | *** |
| genre_sci.fi | 0.48 | *** |
| genre_sport | -0.1 | . |
| genre_thriller | -0.01 |  |
| genre_war | -0.10 | . |
| genre_western | -0.005 |  |

**Table 4.** Output of a linear probability model with the presence of an imaginary world as predicted by the random-forest algorithm as the outcome variable (i.e., binary variable as 0 if it is not set in an imaginary world and 1 if it is), and the genres as the independent variables (i.e., binary variables for each genre with 0 if it is not classified in this genre and 1 if it is). 0 ‘***’ 0.001 ‘**’ 0.01 ‘*’ 0.05 ‘.’ 0.1 ‘ ’ 1.

# 2. Topic Modeling

## 2.1. Algorithm

In the last few years, Natural Language Processing (NLP) has been particularly developed thanks to both enhancement in algorithms and computation power. Deep learning architectures applied to texts - Transformers (Vaswani et al., 2017) - and subsequent algorithms such as BERT (Devlin et al., 2018) have achieved excellent results on different classic NLP tasks such as text prediction, topic modeling or classification. In particular, the architecture of Sbert (Siamese BERT-Networks) (Reimers, 2019) based on Roberta's architecture (Liu, 2019) allows to easily transform a document into a vector, while leaving the choice of the model as a parameter to be easily domain specific. It achieves state-of-the-art performance on machine learning-tasks related to text understanding (Reimers and Gurevych, 2020).

## 2.2. Clustering

Using unsupervised machine learning techniques, we embed the description of the IMDb movie database (n=9424) and project it in Sbert’s latent space. We then used classical techniques of Topic Modeling to create clusters and topic representation. We used KMeans with 7 clusters using the elbow methods. In order to deal with Topic Representation, we extracted the terms using the Textacy python package. We chose to keep bigrams to avoid noises and access straightforward terms. For every cluster, we computed the most specific bigrams using chi2 statistics in order to describe the topic.


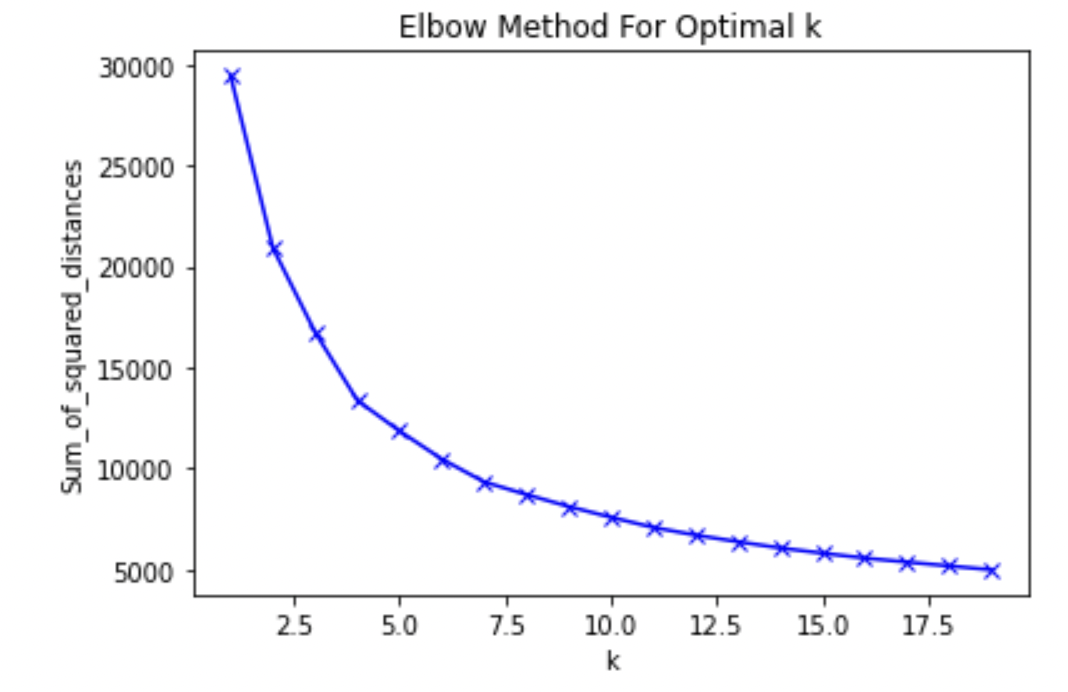


**Figure 1.** Elbow method to define the optimal number of clusters to choose. The right number is the “elbow ” of the curve.

In order to show that there is a relationship between imaginary worlds’s movies and semantics related to exploration, we define a list of core-terms about exploration ('exploration 'explorer', 'explorers', 'explores', 'exploring') and extended it using the latent space of Sbert applied on the description of the movies. In other words, we find the terms that are closest to exploration in the dataset of the movie’s plots. The cosine distance between two terms in that space is a proxy to terms similarity. In other words, the algorithms of bert have learned to associate terms based on a pattern of co-occurrence and neighboring use. If some bias exists due to the dataset they have been trained on, they show good results when they are verified by individuals (Mikolov et al., 2013; Pennington et al., 2014).


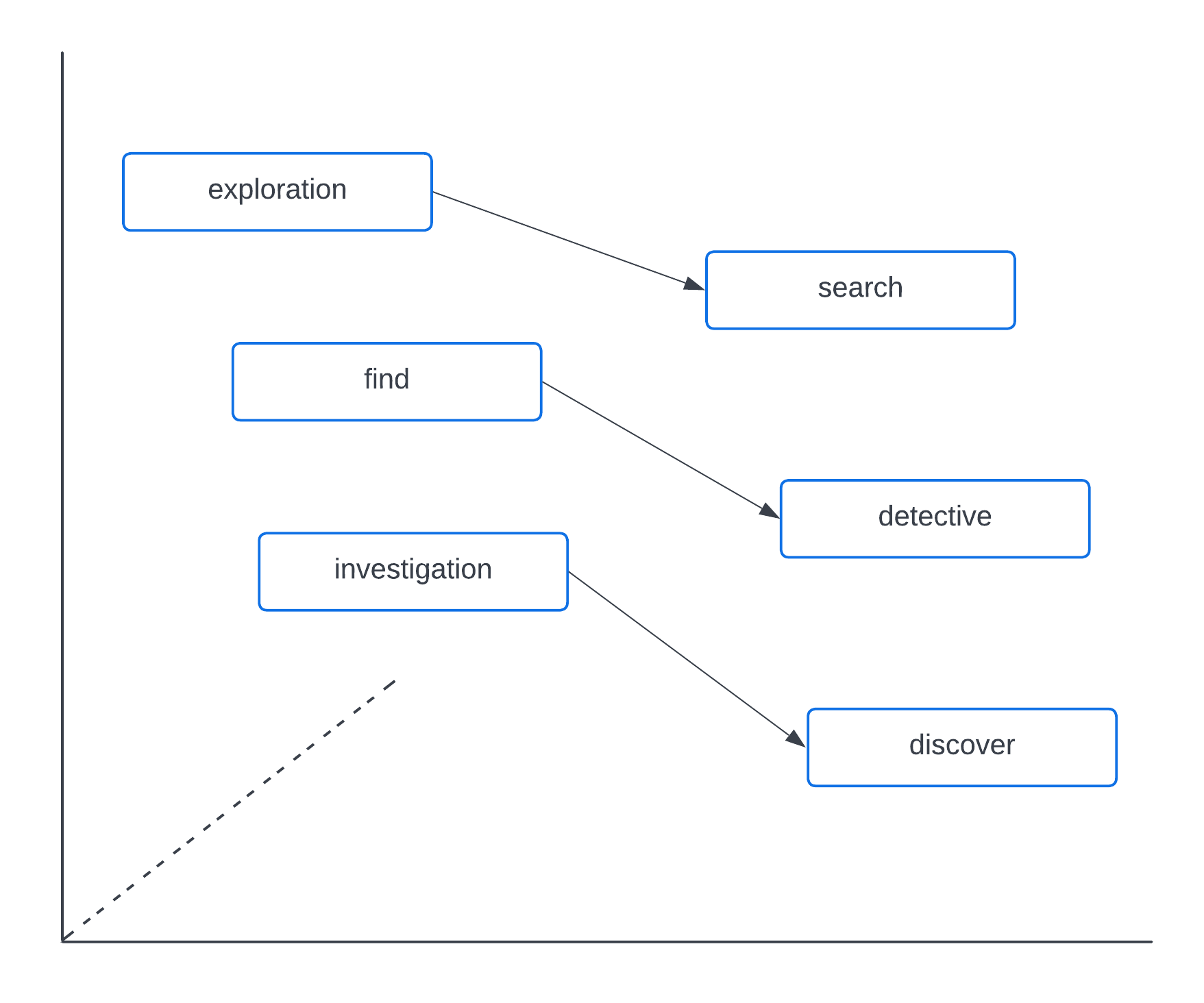


**Figure 2.** Representation of cosine distance between exploration-related terms in Sbert’s latent space.

# 3. Experiments

## 3.1. Self-reporting paradigm

Participants were presented with the following instructions. Each question was followed with a 7-Likert Scale from ‘Strongly disagree’ to ‘Strongly agree’.

They were asked:

*Rate the statements below for how accurately they reflect what you like. Please be as honest as possible.*

*I like movies, novels, and video games…*

*1. … with more information about the world than about the characters`*

*2. … in which the fictional characters explore their environment*

*3. … with novel and surprising technologies*

*4. … which make me feel I am traveling in a foreign world*

*5. … with familiar places*

*6. … which take place in places that exist in the real world*

*7. … which make me feel like I am home*

*8. … with more information about the characters than about the world*

First, we performed a factor analysis to revise the scale and improve the psychometric properties of the individual-difference measure. We used a Principal Axis Factoring method with a Varimax rotation method and a cut-off at .3 (scree plot indicated that a 2-factor analysis was the best solution). KMO sampling adequacy is 0.62 and Bartlett’s K-squared is 25.16 (df = 7, p-value = 0.0007107).

| Factor | 1 | 2 |
| --- | --- | --- |
| exist | 0.64 |  |
| familiar | 0.88 |  |
| home | 0.56 |  |
| explore |  | 0.63 |
| technology |  | 0.75 |
| foreign |  | 0.58 |
| character |  |  |
| world |  | 0.36 |
|  | | |
| SS loadings | 1.56 | 1.46 |
| Proportion Var | 0.19 | 0.18 |
| Cumulative Var | 0.19 | 0.38 |

**Table 6.** Factor loading of the 8 items capturing the self-reported preference for imaginary worlds.

Test of the hypothesis that 2 factors are sufficient. The chi square statistic is 60.19 on 13 degrees of freedom.


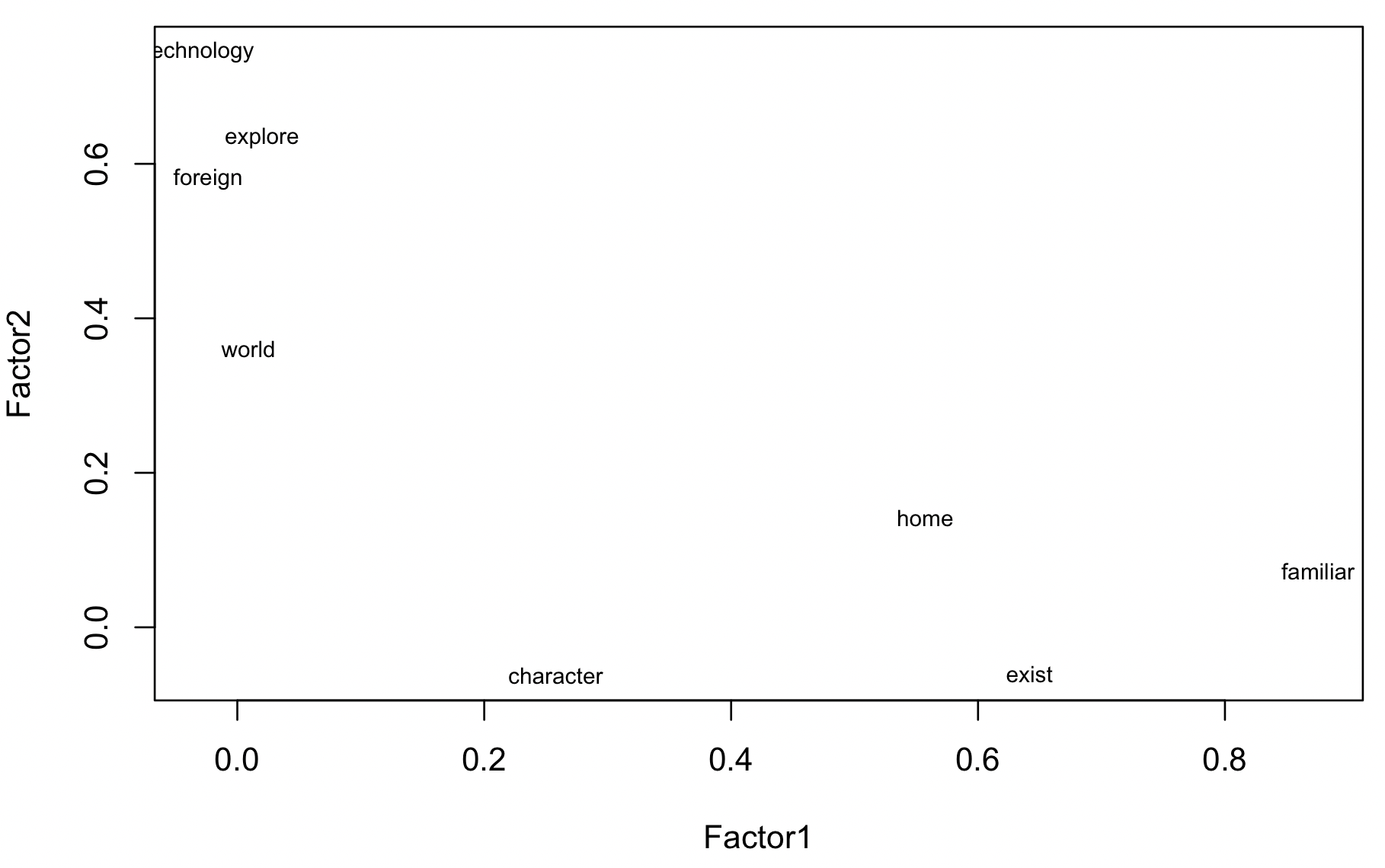


**Figure 3.** Visual representation of the factor analysis.

The 4 items of factor 2, with items directly related to a preference for imaginary worlds, constitute the Imaginary-World Preference score 1 (IWP1). The statistical tests with this score are the ones reported in the paper.

## 3.2. Real Setting Preference: A control variable (non-pre-registered)

Interestingly, the 3 items that load onto Factor 1 are the ones that we had planned to reverse (the items related to fictions with familiar or real places, as opposed to imaginary worlds). It suggests that liking imaginary worlds is not the opposite of liking existing familiar places. We can use this scale, without reversing it, as a score of preference for real settings in fictions and test the same predictions. We will call this score the RSP (Real Setting Preference). We present the results of statistical tests below.

## 3.3. ‘Randomized plots’ paradigm

We created 10 locations (5 imaginary, 5 real) and 10 short movie plots, inspired by real movies.

Locations:

Imaginary worlds:

- In another galaxy,…

- In an alternate version of earth,…

- In a Mars colony,…

- Far in the future,…

- In a parallel world,…

Not imaginary worlds:

- In New York City,…

- In rural Europe,…

- In a small town,…

- In Tokyo,…

- In South America,…

Plots:

- …a scientist seeks revenge on the surgeons responsible for her child’s death.

- …four strangers find themselves in a maze and must cooperate to survive.

- …a group of researchers discover a way to enhance humans’ physical strength.

- …a couple wants to repair their marriage by radically changing their life.

- …a teen musician has an affair with his father’s girlfriend.

- …a woman confronts her parents to know the truth about her family’s past.

- …a teenager wakes up with distorted memories about his life.

- …an assassin is given a second chance.

- …a hunter helps an agent to investigate the disappearance of twenty children.

- …a mysterious sickness starts spreading and only a failed politician realizes it.

Each participant was presented with the 10 plots, with randomly picked locations (5 imaginary and 5 real). They were asked:

*From 1 to 10, how much do you want to watch this movie?*

*1: not at all*

*2*

*3*

*4*

*5*

*6*

*7*

*8*

*9*

*10: very much*

The ‘imaginary world preference’ score IWP2 is computed by adding the mean of all ratings for imaginary-location plots and subtracting the mean of all ratings for real-location plots for a given participant.

## 3.4. ‘Real movies’ paradigm

First, we selected only recent movies (after 2000). Then, we randomly selected 50 movies with imaginary worlds and 50 films with no imaginary world (see Dubourg et al., 2021). We manually checked each film, to be sure that the 50 films with imaginary worlds had an explicit imaginary world mentioned in the plot summary, and that the other 50 did not. When it was not the case according to both coders (E.D. and V.T.) for a given movie, we randomly selected another movie to replace it. We ended up with 2 lists of 50 movies each: 50 movies with imaginary worlds and 50 movies with no imaginary world.

Participants were presented with pairs of 2 movies, chosen randomly. For each film, there were the following information: the film poster and the short summary (from IMDb).

First, participants were asked if they had seen one or two of these movies (suggesting they could be biased toward one or the other based on information imbalance).

If they answered ‘yes’, they were asked which one they had seen.

If they answered ‘no’, they were asked:

*Which film would you prefer to watch? Drag the cursor to the left side or the right side to represent how strong your preference is for the movie you would rather choose. You can leave the cursor at the center if you don't prefer one film or the other.*

And they were able to drag a cursor toward one film or the other (from -10 for the extreme preference for the film with no imaginary world, to 10 for the extreme preference for the film with an imaginary world).

They did this for 10 pairs.

The ‘imaginary world preference’ score IWP3 was computed by averaging all the ratings for a given participant.

## 3.5. Results

First, all three scores are positively and significantly correlated with each other, suggesting that we do capture a consistent preference for imaginary worlds.


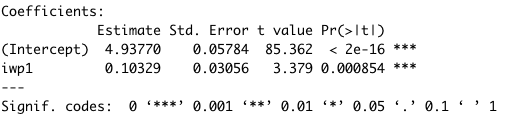


**Figure 4.** Model output of a linear model between IWP1 (self-reported paradigm) and IWP2 (randomized-plot paradigm).


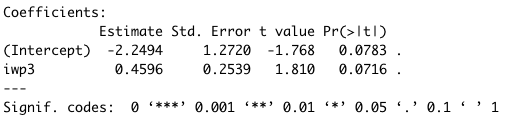


**Figure 5.** Model output of a linear model between IWP1 (self-reported paradigm) and IWP3 (real-movies paradigm).


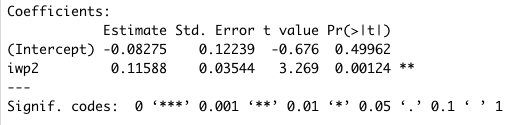


**Figure 6.** Model output of a linear model between IWP2 (randomized-plot paradigm) and IWP3 (real-movies paradigm).

Now, we report the results of our pre-registered predictions with the scores of preference IWP2 and IWP3. The results with IWP1 are reported in the paper (see Appendix B for model assumptions checks). We also report the results of the same tests with the RSP (reported measure of preference for real setting). We didn’t pre-register this, but we expect that it will *not* correlate with our explanatory variables as predicted for the measures of preference for imaginary worlds (we expect the results to be either non-significant or reversed).

| **No.** | **Prediction** | **Test** | **IWP2 (randomized-plot paradigm)** | **IWP3**  **(real-movies paradigm)** | **RSP (Real Setting Preference) from the self-reporting paradigm (not pre-registered)** |
| --- | --- | --- | --- | --- | --- |
| *Core prediction* | | | | | |
| **P1** | More exploratory people prefer movies with imaginary worlds | Linear model (iw ~cei) | **No**  (ß=-0.12, p= 0.302) | **No (reverse)**  (ß=-0.44, p= 0.04) | **Not significant**  ß=0.10, p=0.0657 |
| *Developmental psychology* | | | | | |
| **R1** | Younger people are more exploratory | Linear model (cei ~ age) | **No**  (ß-0.004, p=0.3) | |  |
| **P2** | Younger people prefer more imaginary worlds | Linear model (iw ~ age) | **No**  (ß=-0.003, p=0.662) | **No**  (ß=0.156, p=0.156) | **Positive** (reverse from the prediction)  (ß=0.006, p<.01) |
| **P3** | Exploratory preferences mediate effects of age on the preference for imaginary worlds | Bootstrapping procedure using R mediation package | **Not tested** | **Not tested** |  |
| *Phenotypic plasticity* | | | | |  |
| **R2** | People higher in socio-economic status are more exploratory | Linear model (cei ~ ses) | **Yes**  (ß=0.21, p<0.001) | | |
| **P4** | People higher in socio-economic status prefer more imaginary worlds | Linear model (iw ~ ses) | **No (reverse)**  (ß=-0.25, p<0.05) | **No**  (ß=-0.34, p=0.059) | **Not significant**  (ß=0.07, p=0.143) |
| **P5** | Exploratory preferences mediate effects of SES on the preference for imaginary worlds | Bootstrapping procedure using R mediation package | **Not tested** | **Not tested** |  |
| *Systemizing* | | | | | |
| **RQ2** | More systemizing people are more exploratory | Linear model (cei ~ sq) | **Yes**  (ß=0.485, p<0.001) | | |
| **P6** | More systemizing people prefer more imaginary worlds | Linear model (iw ~sq) | **No**  (ß=-0.06, p=0.66) | **No**  (ß=-0.27, p=0.240) |  |
| *Sexual dimorphism* | | | | | |
| **R3** | Men are higher in systemizing | Linear model (sq ~ sex) | **Yes**  (-0.76, p<0.001) | | |
| **P7** | Men prefer more imaginary worlds | Linear model (iw ~ sex) | **No**  (-0.04, p=0.882) | **No**  (-0.51, p=0.258) | **More female** (reverse from the prediction)  (ß=0.20, p<.1 |
| **P8** | Systemizing mediates the effects of sex on the preference for imaginary worlds | Bootstrapping procedure using R mediation package | **Not tested** | **Not tested** |  |
| *Alternative hypothesis* | | | | | |
| **P9** | Lower well-being does not increase the preference for imaginary worlds | Linear model (iw ~ wb) | **Yes** (not significant) | **Yes** (not significant) | **Significant** (reverse from prediction)  (ß= 0.25, p<.01) |
| *Proxy and design validation* | | | | | |
| **R4** | More exploratory people are higher in Openness to experience | Linear model (cei ~ ope) | **Yes**  (0.3, p<0.001) | |  |
| **P10** | People higher in Openness to experience prefer movies with imaginary worlds | Linear model (iw ~ ope) | **No**  (ß=0.05, p=0.644) | **No**  (ß=-0.25, p=0.171) | **Not significant**  (ß=0.04, p=0.367) |

**Table 7**. Summary of the predictions, statistical tests, and results of the experimental study with the self-reporting paradigm, as pre-registered (P=Prediction, R=Replication, RQ=Research Question). We removed from the pre-registration 2 mediation tests that we could not perform (see Pre-registration).

Contrary to what we report in the paper with IWP1 (the score of preference for imaginary worlds from the self-reporting paradigm), we find little support for our hypothesis with IWP2 and IWP3. Because the paradigm that worked is the most used in similar research, the most straightforward, and the most consistent with the results from the observational study with large-scale datasets, we reason that the other two paradigms, while capturing something related to imaginary worlds (all three scores correlated with each other), are not precise enough to test our predictions. More specifically, in the ‘randomized-plot’ and ‘real-movies’ paradigms, participants could base their choices on other features than the presence of an imaginary world. This means that we should have a much bigger sample to capture a very small effect (the effect of the presence of an imaginary world independently of all other features from the fictions).

As a check of the self-reporting paradigm, we performed all the analyses with 3 items that we had planned to reverse, and that, with no reversal, seemed to capture the preference for real settings. We expected that this score should not correlate as the score of preference for imaginary worlds. Indeed, people higher in the Curiosity and Exploration Inventory scale don’t enjoy more fictions with real settings (whereas they enjoy more fictions with imaginary worlds; see article). People who enjoy more fictions with real settings are more likely to be females (while people who enjoy more fictions with imaginary worlds are more likely to be males; see article). The socio-economic status of participants doesn’t correlate with the score of preference for fictions with real settings. People higher in well-being enjoy more fictions with real settings (whereas, as predicted, we found no correlation between scores of well-being and scores of preference for fictions with imaginary worlds; see article). And lastly, people higher in Openness didn’t enjoy more fictions with real settings (as they did for fictions with imaginary words; see article).

## 3.5. Results before exclusion

Because we added more measurements in the course of the experiment, and because not all participants from the first run responded to the next ones, we lost some participants. We add here the results of the tests that we could do after the very first run, before excluding the participants that did not participate to the next ones:

- Correlation between IWP2 and IWP3: positive and significative (ß=.14, p<.001)
- Correlations between IWP2-3 and CEI: not significative
- Correlation CEI and Age: negative and near-signiciative (ß=-0.005, p=0.08)
- Correlation IWP2 and Age: not significative
- Correlation between IWP3 and Age: negative and signiciative (ß=-0.03, p<.01)
- Correlation between CEI and SES: positive and significative (ß= 0.11, p<.01)
- Correlations between IWP2-3 and SES: not significative
- Correlation between SQ and CEI: positive and significative (ß= 0.43, p<.001)
- Correlations between SQ and IWP2-3: not significative
- Correlation between Sex and SQ: negative and significative (ß=-0.7, p<.001)
- Correlations between IWP2-3 and WB: not significative
- Correlation between Openness and CEI: positive and significative (ß=.29, p<.001)
- Correlations between IWP2-3 and Openness: not significative

# Appendix A: Movies manually annotated

| **imdb** | **coder** | **imaginary world** | **imdb** | **coder** | **imaginary world** |
| --- | --- | --- | --- | --- | --- |
| tt4562518 | ed | 0 | tt0120772 | vt | 0 |
| tt4972062 | ed | 0 | tt1183923 | ed | 0 |
| tt0090021 | ed | 1 | tt0404390 | ed | 0 |
| tt0104438 | ed | 0 | tt1828995 | ed | 0 |
| tt0018037 | ed | 0 | tt5742374 | ed | 0 |
| tt0455967 | ed | 0 | tt2273657 | ed | 0 |
| tt0298296 | ed | 0 | tt1986843 | ed | 0 |
| tt4652650 | ed | 0 | tt1082868 | ed | 0 |
| tt0040308 | ed | 0 | tt0081777 | ed | 0 |
| tt0093389 | ed | 0 | tt0804452 | ed | 0 |
| tt1760967 | ed | 0 | tt0105391 | vt | 0 |
| tt2005151 | ed | 0 | tt0088272 | vt | 0 |
| tt0166813 | ed | 0 | tt0137338 | vt | 0 |
| tt0326977 | ed | 0 | tt0439662 | vt | 0 |
| tt1663143 | ed | 0 | tt1578275 | vt | 0 |
| tt0326208 | ed | 0 | tt0078754 | vt | 0 |
| tt1937264 | ed | 0 | tt4270516 | vt | 0 |
| tt0033922 | ed | 0 | tt0056412 | vt | 0 |
| tt0151137 | ed | 0 | tt0112288 | vt | 0 |
| tt5571734 | ed | 0 | tt1540011 | vt | 1 |
| tt0985694 | ed | 0 | tt0104437 | vt | 0 |
| tt0106307 | ed | 0 | tt0099385 | ed | 0 |
| tt0106881 | ed | 0 | tt7618184 | ed | 0 |
| tt2229842 | ed | 0 | tt0166175 | ed | 0 |
| tt1809398 | ed | 0 | tt1362058 | ed | 0 |
| tt0316654 | ed | 0 | tt4438848 | ed | 0 |
| tt0087469 | ed | 1 | tt0109842 | ed | 0 |
| tt0066999 | ed | 0 | tt0365265 | ed | 0 |
| tt2396589 | ed | 0 | tt0365376 | ed | 0 |
| tt0486551 | ed | 0 | tt6173990 | ed | 0 |
| tt0105151 | ed | 0 | tt0155388 | vt | 0 |
| tt0489049 | ed | 0 | tt0311429 | vt | 1 |
| tt0489235 | ed | 1 | tt0074486 | vt | 1 |
| tt0089118 | ed | 0 | tt0039417 | vt | 0 |
| tt0124879 | ed | 0 | tt2364975 | vt | 0 |
| tt0338348 | ed | 1 | tt0097202 | vt | 0 |
| tt0055830 | ed | 1 | tt0092610 | vt | 1 |
| tt0435680 | ed | 0 | tt0107977 | vt | 1 |
| tt1413495 | ed | 0 | tt0094898 | vt | 1 |
| tt0113097 | ed | 0 | tt0113189 | vt | 0 |
| tt4273292 | ed | 0 | tt2302755 | vt | 0 |
| tt2296777 | ed | 0 | tt1708135 | ed | 0 |
| tt5117670 | ed | 0 | tt1314228 | ed | 0 |
| tt2406566 | ed | 0 | tt2178470 | ed | 0 |
| tt0093058 | ed | 0 | tt0098273 | ed | 0 |
| tt0078492 | ed | 0 | tt0120832 | ed | 0 |
| tt0103247 | ed | 0 | tt0120868 | ed | 0 |
| tt0105459 | ed | 1 | tt0053793 | ed | 0 |
| tt0493949 | ed | 0 | tt0273435 | ed | 0 |
| tt0187738 | ed | 1 | tt0116630 | ed | 0 |
| tt4530422 | ed | 0 | tt1640484 | vt | 0 |
| tt0110366 | ed | 0 | tt0117958 | vt | 0 |
| tt1854236 | ed | 0 | tt2258858 | vt | 0 |
| tt1714209 | ed | 0 | tt1395054 | vt | 0 |
| tt1674773 | ed | 0 | tt0092086 | vt | 0 |
| tt1264904 | ed | 0 | tt0120824 | vt | 0 |
| tt0069280 | ed | 1 | tt0277371 | vt | 0 |
| tt6303866 | ed | 0 | tt0084549 | vt | 0 |
| tt0102015 | ed | 0 | tt1605630 | vt | 1 |
| tt0247745 | ed | 0 | tt1117385 | vt | 0 |
| tt1401152 | ed | 0 | tt2390237 | ed | 0 |
| tt1784499 | ed | 0 | tt0061395 | ed | 0 |
| tt0399854 | ed | 0 | tt0783238 | ed | 0 |
| tt0054047 | ed | 0 | tt1216492 | ed | 0 |
| tt0372183 | ed | 0 | tt0128442 | ed | 0 |
| tt1862079 | ed | 0 | tt2481480 | ed | 0 |
| tt0368658 | ed | 0 | tt0057058 | ed | 0 |
| tt0297721 | ed | 1 | tt5440700 | ed | 0 |
| tt2125666 | ed | 0 | tt0450336 | ed | 0 |
| tt0116231 | ed | 0 | tt0112346 | ed | 0 |
| tt2917388 | ed | 0 | tt1417592 | vt | 0 |
| tt1587310 | ed | 1 | tt1411697 | vt | 0 |
| tt2070649 | ed | 0 | tt0245803 | vt | 0 |
| tt0815245 | ed | 0 | tt0311289 | vt | 1 |
| tt0893382 | ed | 0 | tt0251114 | vt | 0 |
| tt1911600 | ed | 0 | tt7014006 | vt | 0 |
| tt1285241 | ed | 0 | tt0113492 | vt | 1 |
| tt4776998 | ed | 0 | tt0472582 | vt | 0 |
| tt0286306 | ed | 0 | tt0082736 | vt | 0 |
| tt0016847 | ed | 0 | tt0175526 | vt | 1 |
| tt1731701 | ed | 0 | tt1179933 | ed | 0 |
| tt0340855 | ed | 0 | tt0042192 | ed | 0 |
| tt1682181 | ed | 0 | tt0377109 | ed | 0 |
| tt0481369 | ed | 0 | tt0277296 | ed | 1 |
| tt0120689 | ed | 0 | tt0199725 | ed | 0 |
| tt2752200 | ed | 0 | tt2965466 | ed | 0 |
| tt0122515 | ed | 0 | tt0118604 | ed | 0 |
| tt0046487 | ed | 0 | tt0120772 | ed | 0 |
| tt0088847 | ed | 0 | tt0071315 | ed | 0 |
| tt1333667 | ed | 0 | tt0386342 | ed | 0 |
| tt0016332 | ed | 0 | tt1980209 | vt | 0 |
| tt5638642 | ed | 0 | tt0445935 | vt | 0 |
| tt3707106 | ed | 0 | tt0022286 | vt | 0 |
| tt0420087 | ed | 0 | tt0822832 | vt | 0 |
| tt4262980 | ed | 0 | tt0362590 | vt | 0 |
| tt3280262 | ed | 0 | tt0092603 | vt | 0 |
| tt0062622 | ed | 1 | tt6628102 | vt | 0 |
| tt0095882 | ed | 0 | tt0493405 | vt | 0 |
| tt1226753 | ed | 0 | tt0044008 | vt | 0 |
| tt0133412 | vt | 0 | tt0084726 | vt | 1 |
| tt0970866 | vt | 0 | tt0065421 | ed | 0 |
| tt0076451 | vt | 0 | tt0452681 | ed | 1 |
| tt0059797 | vt | 0 | tt0425079 | ed | 0 |
| tt0069191 | vt | 0 | tt0809504 | ed | 0 |
| tt3205376 | vt | 0 | tt2388715 | ed | 0 |
| tt3235888 | vt | 0 | tt0055572 | ed | 0 |
| tt2309021 | vt | 0 | tt0780548 | ed | 0 |
| tt0289992 | vt | 0 | tt4443658 | ed | 0 |
| tt5164184 | vt | 0 | tt2631186 | ed | 0 |
| tt0960790 | vt | 0 | tt0435705 | ed | 0 |
| tt0167261 | vt | 1 | tt0107497 | vt | 0 |
| tt0314498 | vt | 0 | tt2459156 | vt | 0 |
| tt0351817 | vt | 0 | tt0310775 | vt | 0 |
| tt0045883 | vt | 0 | tt0496436 | vt | 0 |
| tt0259393 | vt | 0 | tt1937149 | vt | 0 |
| tt0265651 | vt | 0 | tt0107315 | vt | 0 |
| tt0110527 | vt | 0 | tt0067116 | vt | 0 |
| tt0319020 | vt | 0 | tt2178941 | vt | 0 |
| tt0454824 | vt | 0 | tt0089961 | vt | 1 |
| tt3347976 | vt | 0 | tt5098128 | vt | 0 |
| tt0051207 | vt | 0 | tt0312843 | vt | 0 |
| tt0410297 | vt | 0 | tt0104647 | vt | 0 |
| tt0386117 | vt | 1 | tt0043278 | vt | 0 |
| tt1583420 | vt | 0 | tt0097981 | vt | 0 |
| tt0937237 | vt | 0 | tt0247196 | vt | 0 |
| tt0052311 | vt | 0 | tt1895587 | vt | 0 |
| tt4547056 | vt | 1 | tt0119273 | vt | 1 |
| tt1403865 | vt | 0 | tt0989757 | vt | 0 |
| tt0450259 | vt | 0 | tt0092007 | vt | 1 |
| tt0140888 | vt | 0 | tt1477076 | vt | 0 |
| tt0099674 | vt | 0 | tt0060959 | ed | 0 |
| tt0018773 | vt | 0 | tt0889583 | ed | 0 |
| tt0107387 | vt | 0 | tt1092082 | ed | 0 |
| tt1814836 | vt | 0 | tt6304046 | ed | 0 |
| tt0219699 | vt | 0 | tt1549572 | ed | 1 |
| tt0119280 | vt | 0 | tt0054167 | ed | 0 |
| tt0172396 | vt | 0 | tt1153706 | ed | 0 |
| tt1020543 | vt | 1 | tt3721936 | ed | 0 |
| tt0048473 | vt | 0 | tt0089670 | ed | 0 |
| tt0079588 | vt | 1 | tt0085127 | ed | 0 |
| tt1448755 | vt | 0 | tt0133240 | vt | 1 |
| tt0489282 | vt | 0 | tt0104605 | vt | 0 |
| tt2396224 | vt | 0 | tt0112553 | vt | 0 |
| tt0112585 | vt | 0 | tt1216475 | vt | 1 |
| tt0163978 | vt | 1 | tt0308208 | vt | 0 |
| tt0807758 | vt | 1 | tt0405393 | vt | 0 |
| tt0077711 | vt | 0 | tt0101329 | vt | 0 |
| tt0097138 | vt | 1 | tt0493464 | vt | 0 |
| tt0399146 | vt | 1 | tt0892791 | vt | 1 |
| tt0448134 | vt | 1 | tt0217505 | vt | 0 |
| tt0072951 | vt | 1 | tt0450188 | vt | 0 |
| tt0114857 | vt | 1 | tt0109117 | vt | 0 |
| tt1396484 | vt | 0 | tt0116136 | vt | 1 |
| tt0071746 | vt | 0 | tt2097298 | vt | 0 |
| tt6439558 | vt | 0 | tt0356910 | vt | 0 |
| tt1578882 | vt | 0 | tt2167202 | vt | 0 |
| tt0048491 | vt | 0 | tt1075110 | vt | 1 |
| tt3717490 | vt | 1 | tt0282687 | vt | 0 |
| tt0322259 | vt | 0 | tt0024216 | vt | 1 |
| tt0110877 | vt | 0 | tt1560747 | vt | 0 |
| tt0101745 | vt | 0 | tt0095253 | vt | 0 |
| tt4901306 | vt | 0 | tt2479478 | vt | 0 |
| tt0926380 | vt | 0 | tt0765447 | vt | 0 |
| tt0106266 | vt | 0 | tt0384642 | vt | 0 |
| tt1853728 | vt | 0 | tt1270842 | vt | 0 |
| tt1379177 | vt | 0 | tt0094025 | vt | 0 |
| tt1615160 | vt | 0 | tt0320244 | vt | 0 |
| tt0104779 | vt | 0 | tt0844671 | vt | 0 |
| tt0409904 | vt | 1 | tt0120834 | vt | 0 |
| tt1616195 | vt | 0 | tt0278295 | vt | 0 |
| tt6048930 | vt | 0 | tt0219965 | vt | 0 |
| tt1322312 | vt | 0 | tt1226236 | vt | 0 |
| tt0065462 | vt | 1 | tt0120794 | vt | 0 |
| tt0817538 | vt | 0 | tt0075005 | vt | 0 |
| tt1183732 | vt | 1 | tt0211465 | vt | 0 |
| tt0341495 | vt | 0 | tt1954206 | vt | 0 |
| tt1924394 | vt | 0 | tt1990181 | vt | 0 |
| tt0401729 | vt | 1 | tt0103956 | vt | 0 |
| tt0327162 | vt | 0 | tt2283748 | vt | 0 |
| tt3040964 | vt | 1 | tt0118845 | vt | 0 |
| tt0118665 | vt | 0 | tt4154756 | ed | 1 |
| tt1262416 | vt | 1 | tt1504320 | ed | 0 |
| tt3381008 | vt | 0 | tt0046889 | ed | 0 |
| tt0044741 | vt | 0 | tt1725795 | ed | 0 |
| tt0103241 | vt | 0 | tt4385888 | ed | 0 |
| tt1187064 | vt | 0 | tt0473360 | ed | 0 |
| tt3140100 | vt | 0 | tt0061735 | ed | 0 |
| tt0088680 | vt | 0 | tt0118804 | ed | 0 |
| tt0244244 | vt | 0 | tt1959332 | ed | 0 |
| tt0120777 | vt | 0 | tt0350258 | ed | 0 |
| tt0028010 | vt | 0 | tt0783598 | ed | 0 |
| tt0086859 | vt | 0 | tt0286261 | ed | 0 |
| tt0113243 | vt | 0 | tt3495026 | ed | 0 |
| tt2882328 | vt | 0 | tt0386064 | ed | 0 |
| tt0452624 | vt | 0 | tt0098141 | ed | 0 |
| tt0978764 | vt | 1 | tt0113101 | ed | 0 |
| tt0101420 | vt | 1 | tt1630036 | ed | 0 |
| tt1302067 | vt | 0 | tt0449994 | ed | 0 |
| tt4799064 | vt | 0 | tt1230414 | ed | 0 |
| tt3172532 | vt | 0 | tt0790736 | vt | 1 |

#

# Appendix B: Checks of Model Assumptions for Experimental Study

## Distribution of variables

**
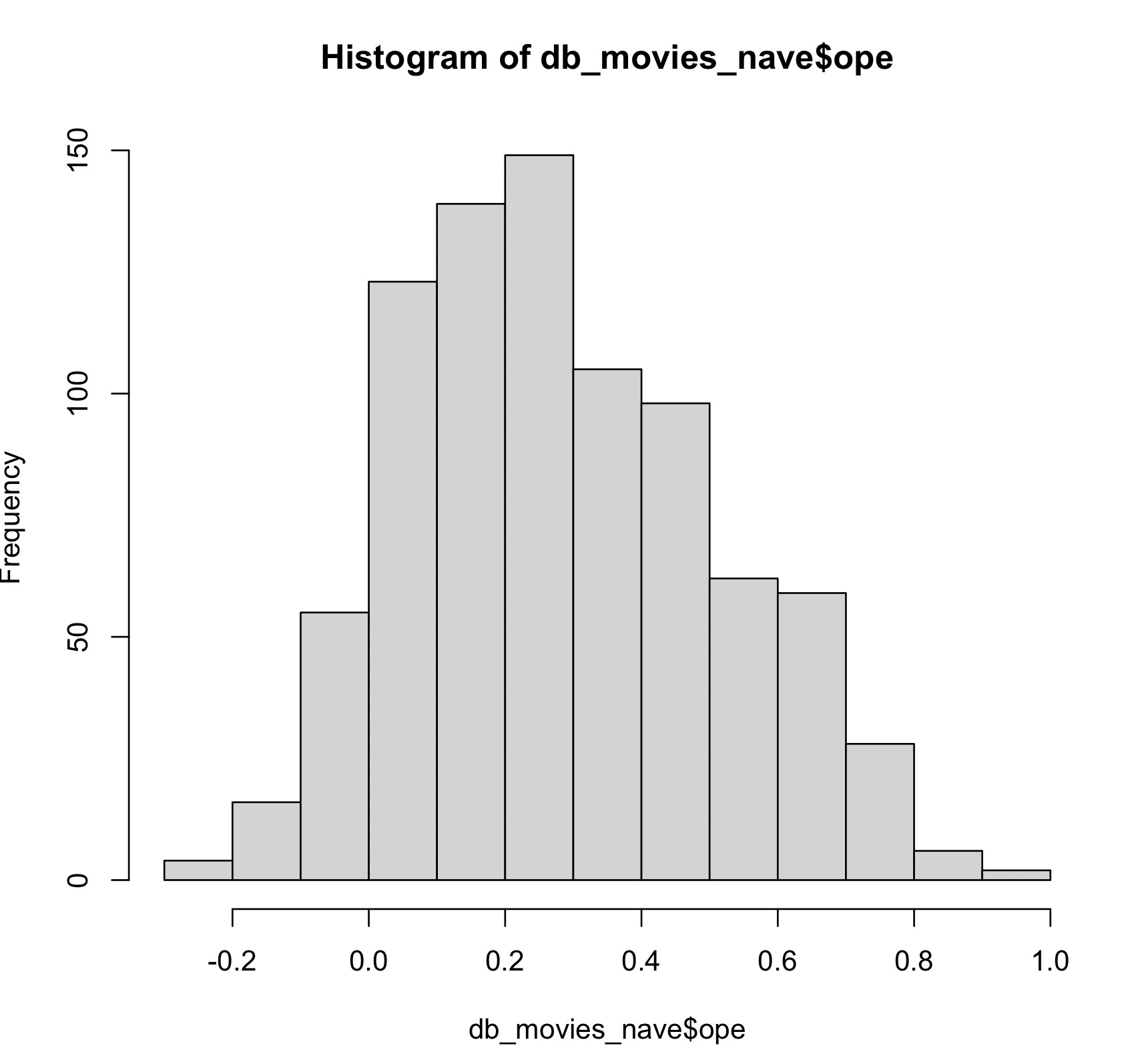
**

**
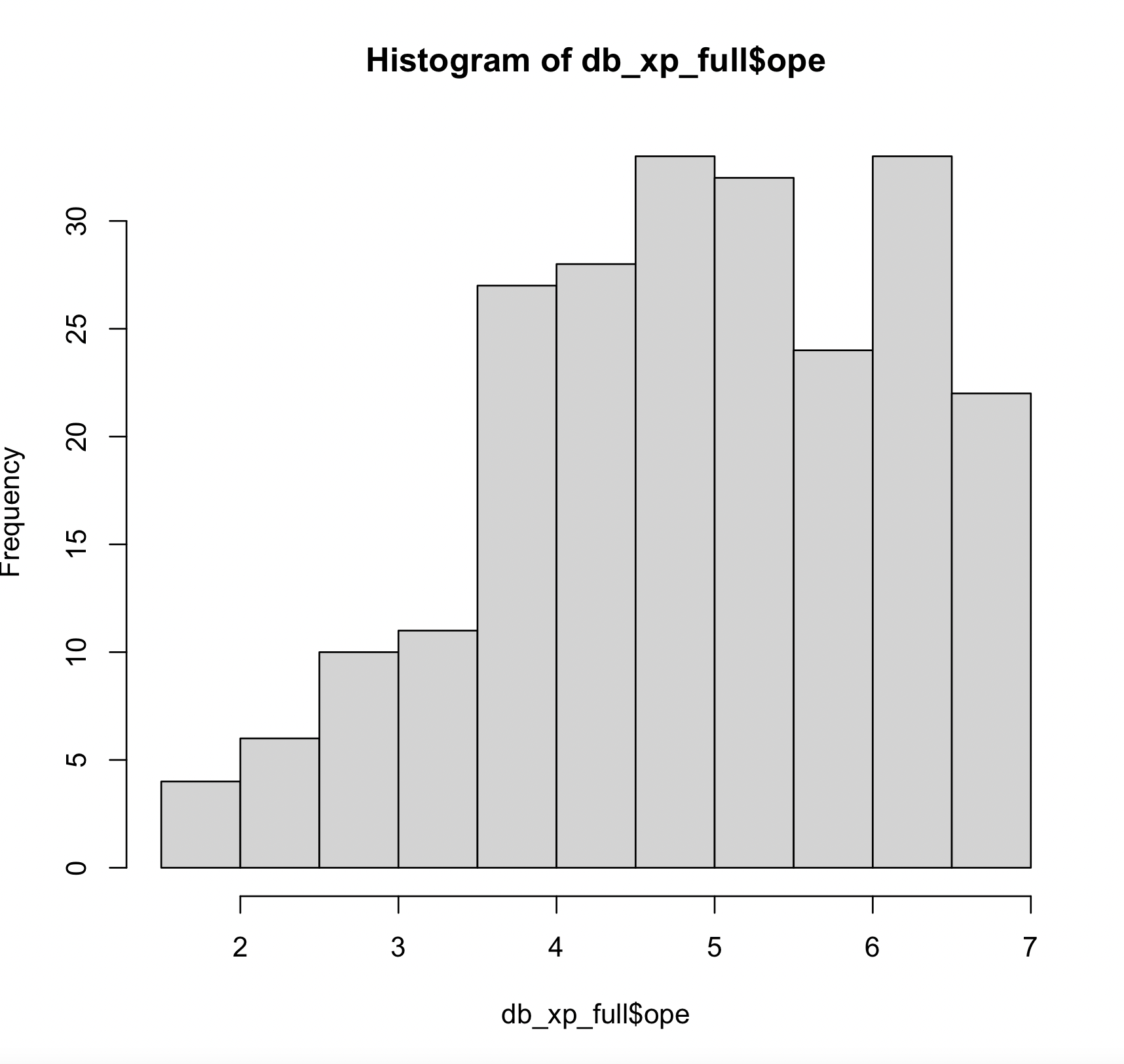

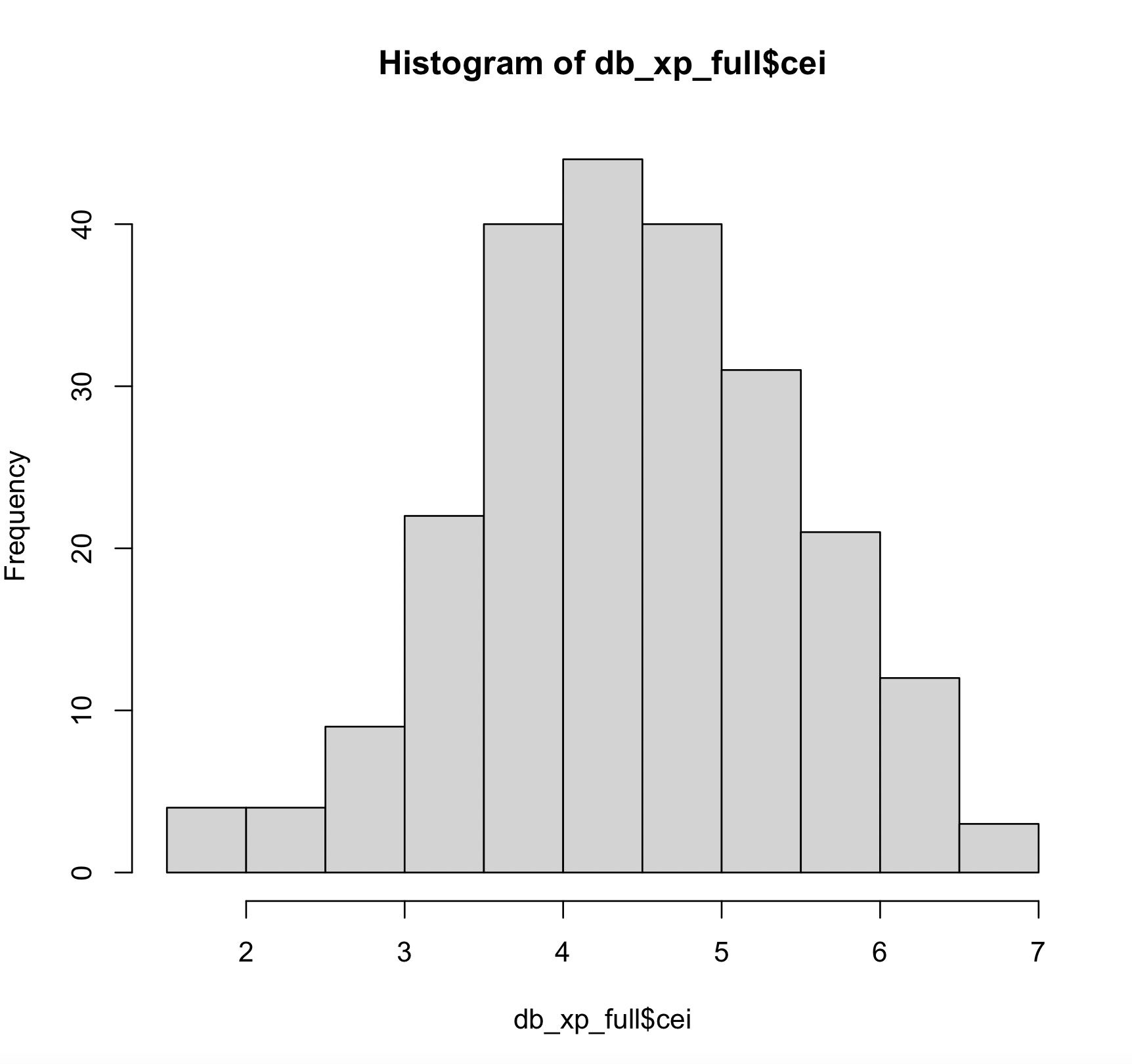

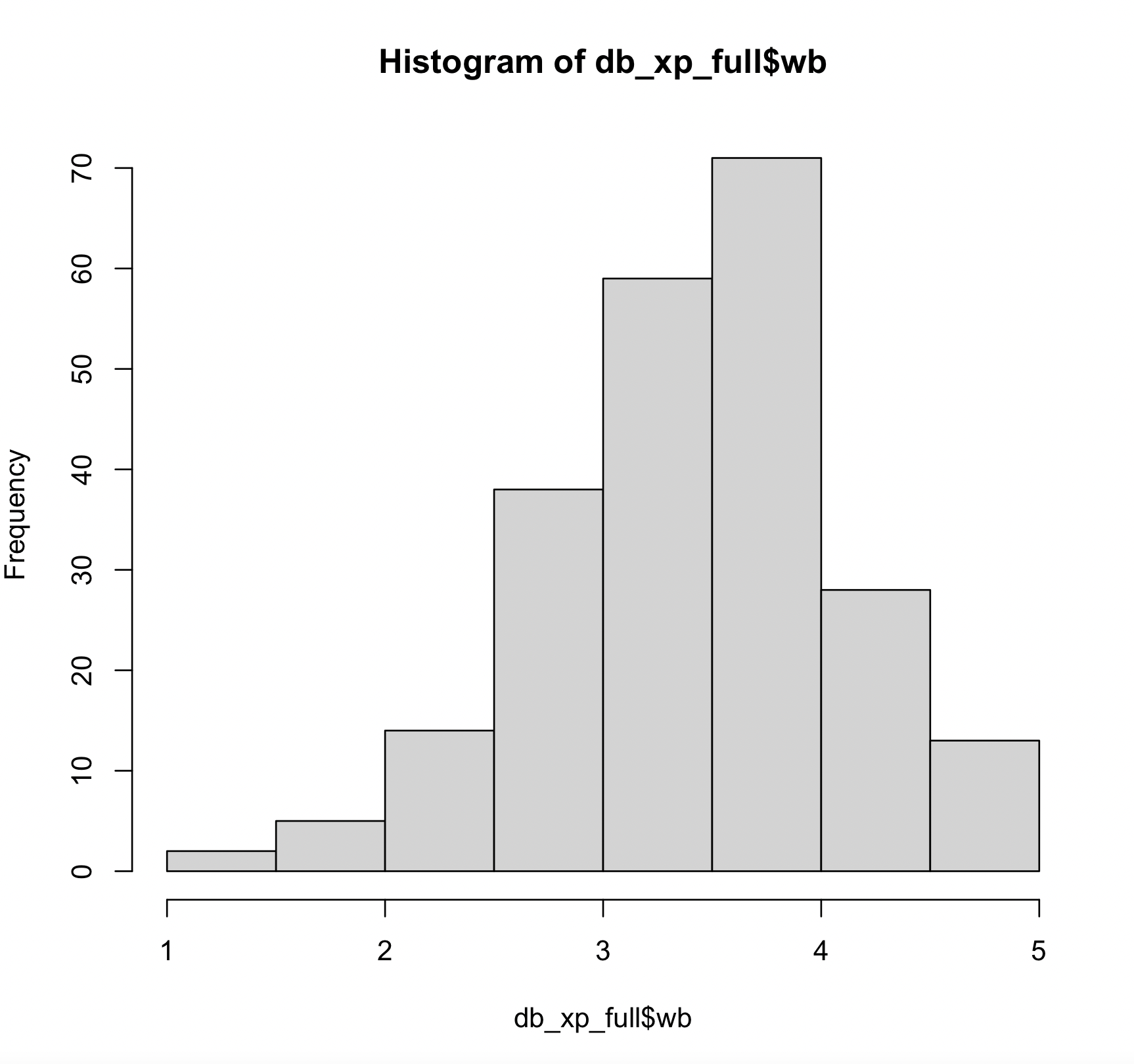

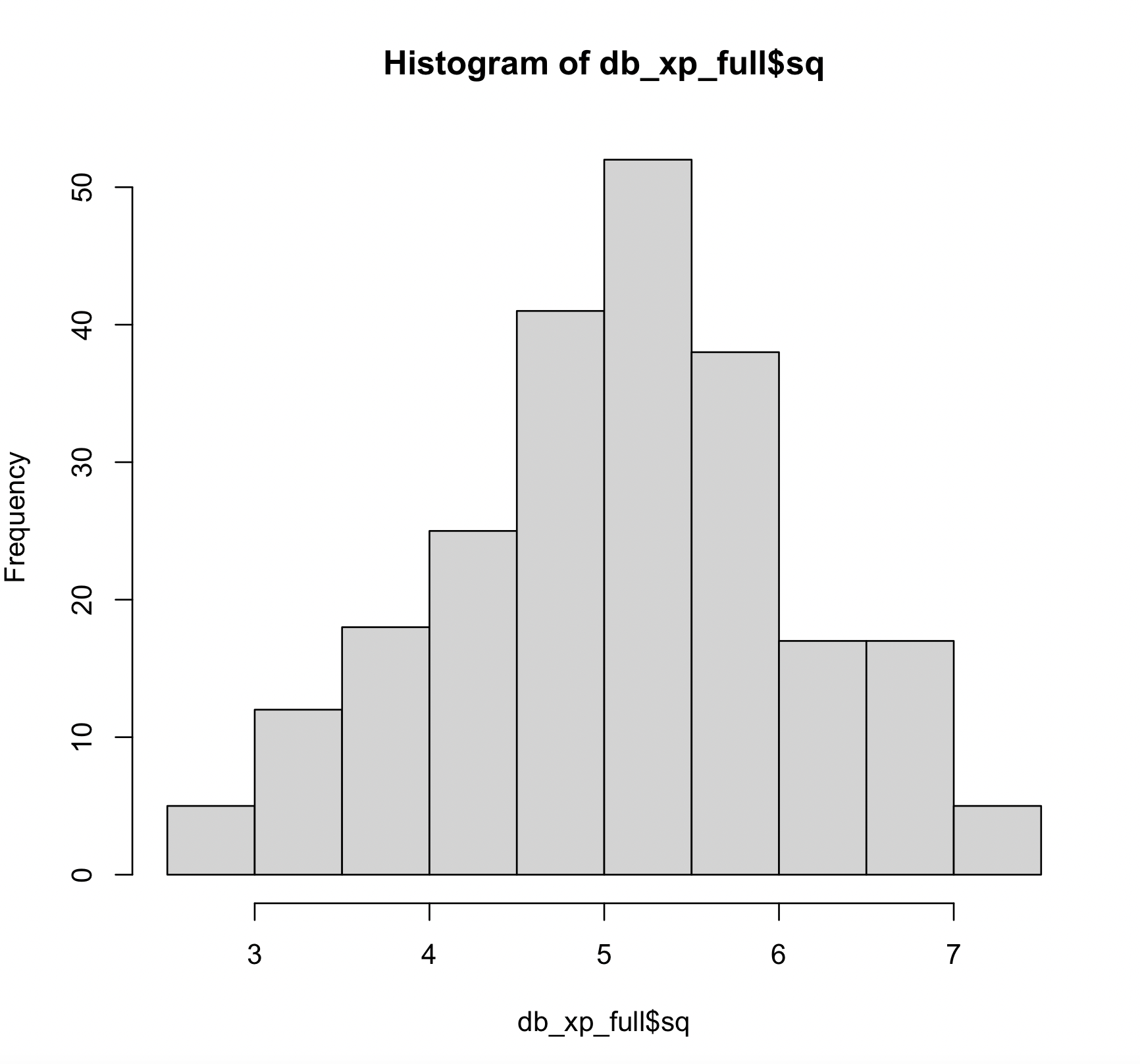

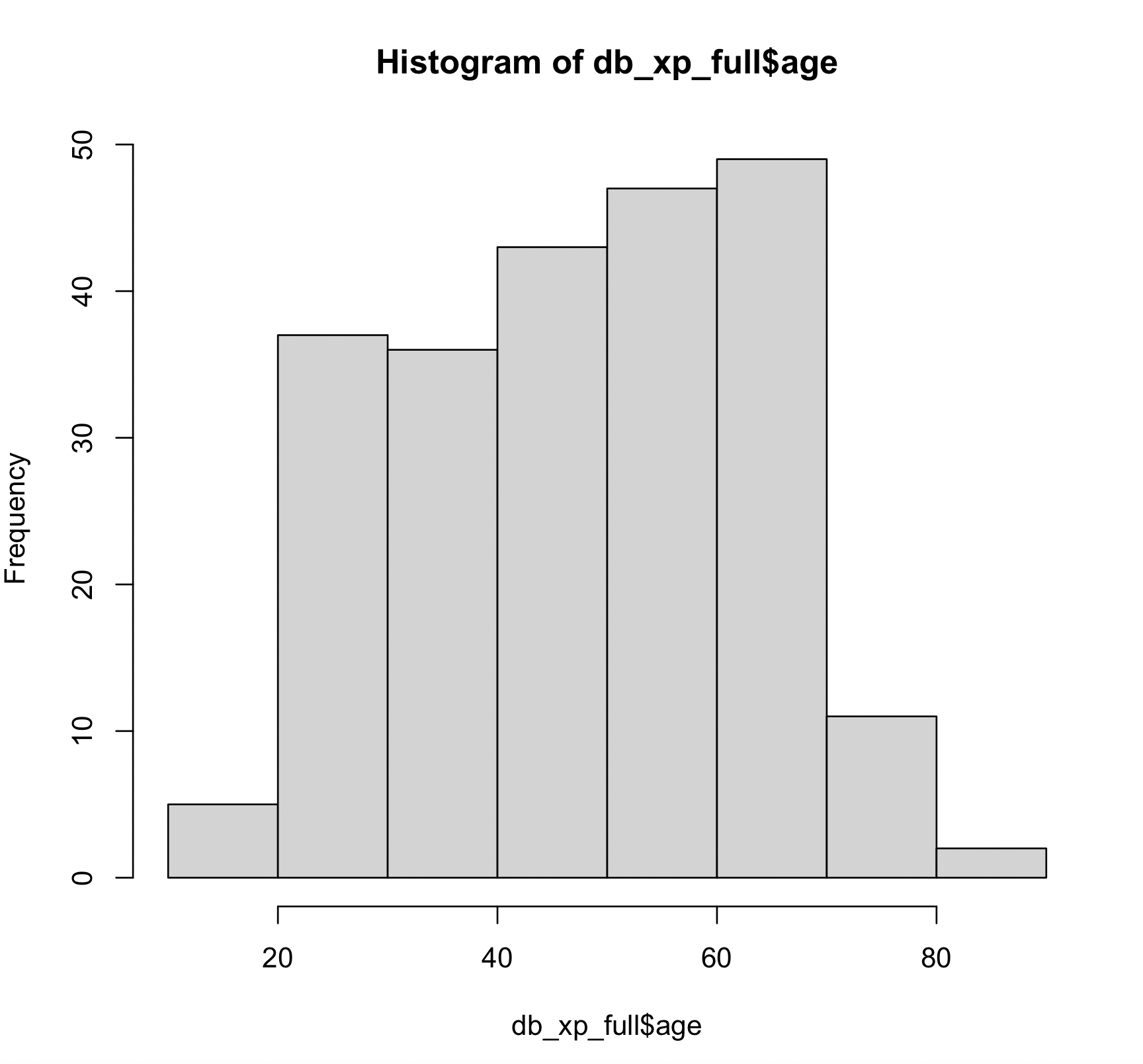

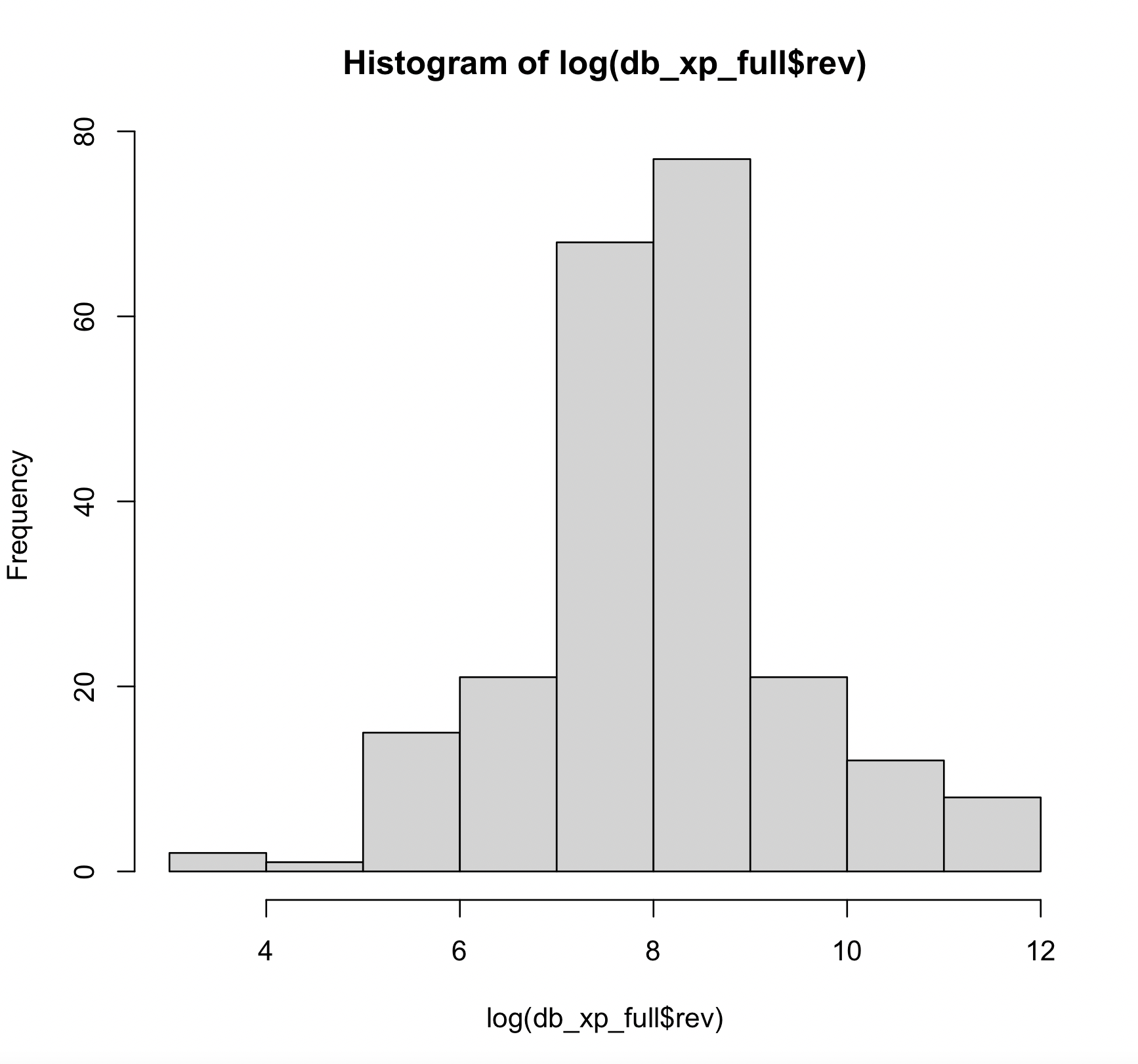

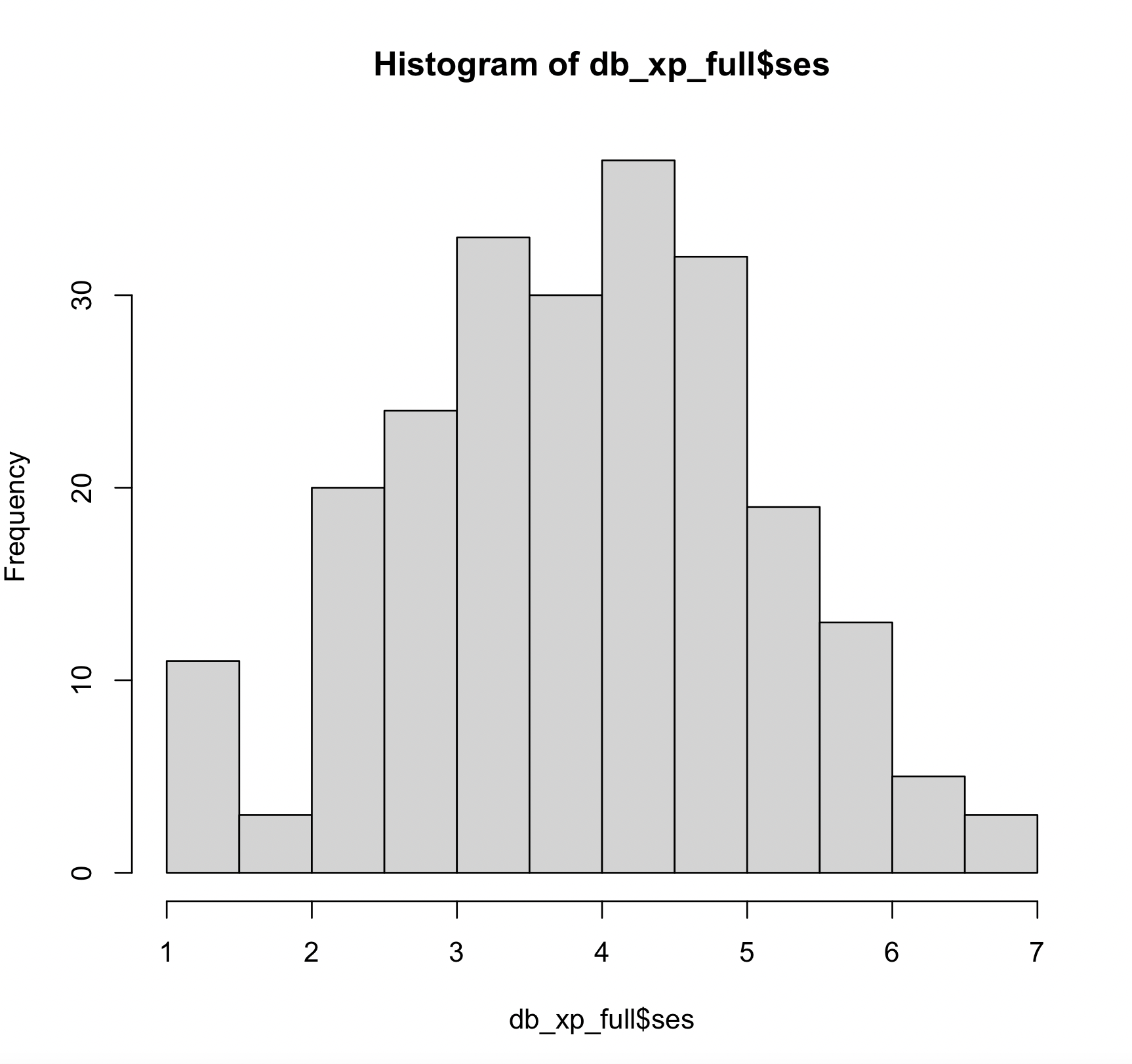

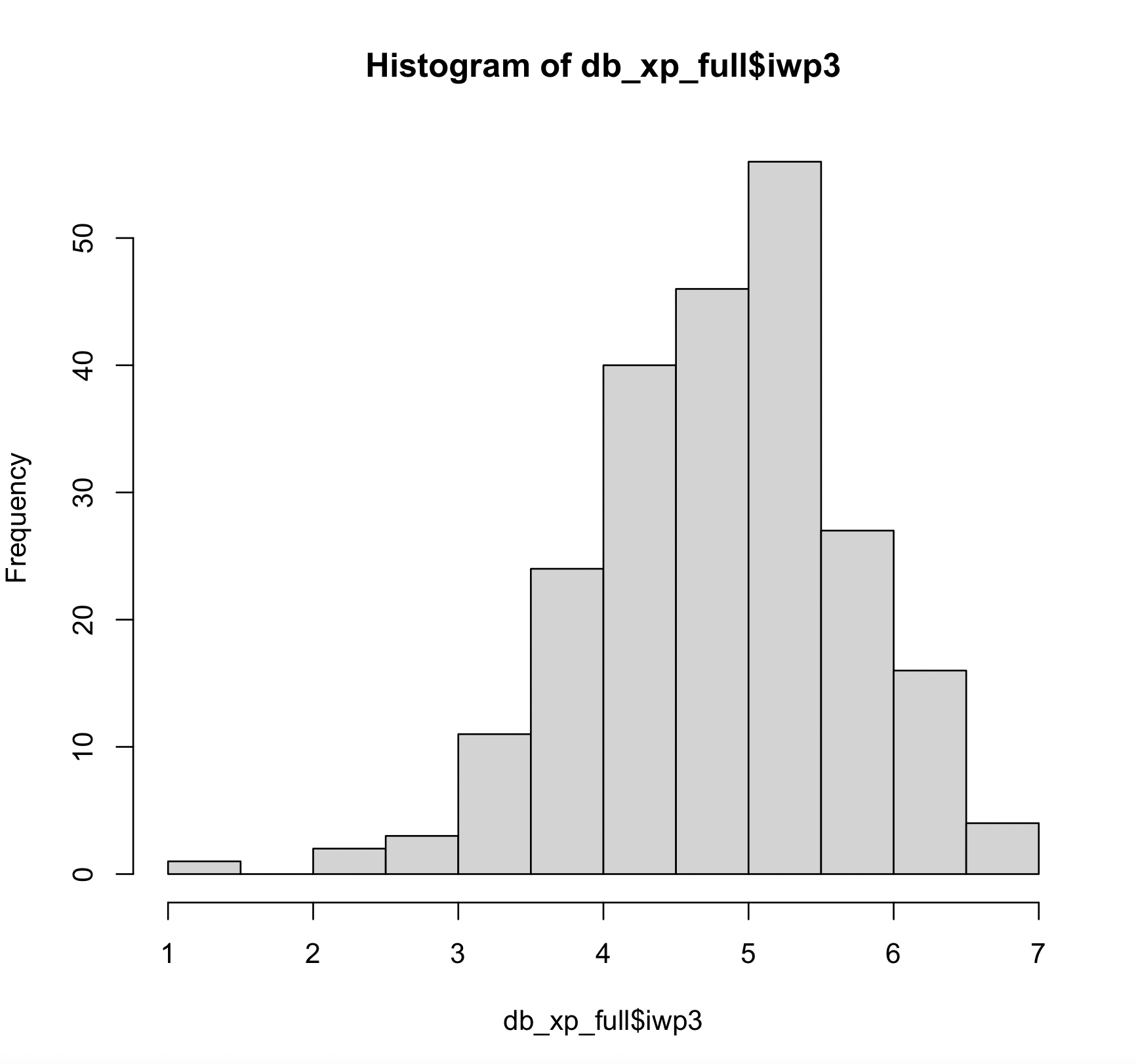
**

## Model assumptions

**For P1:**


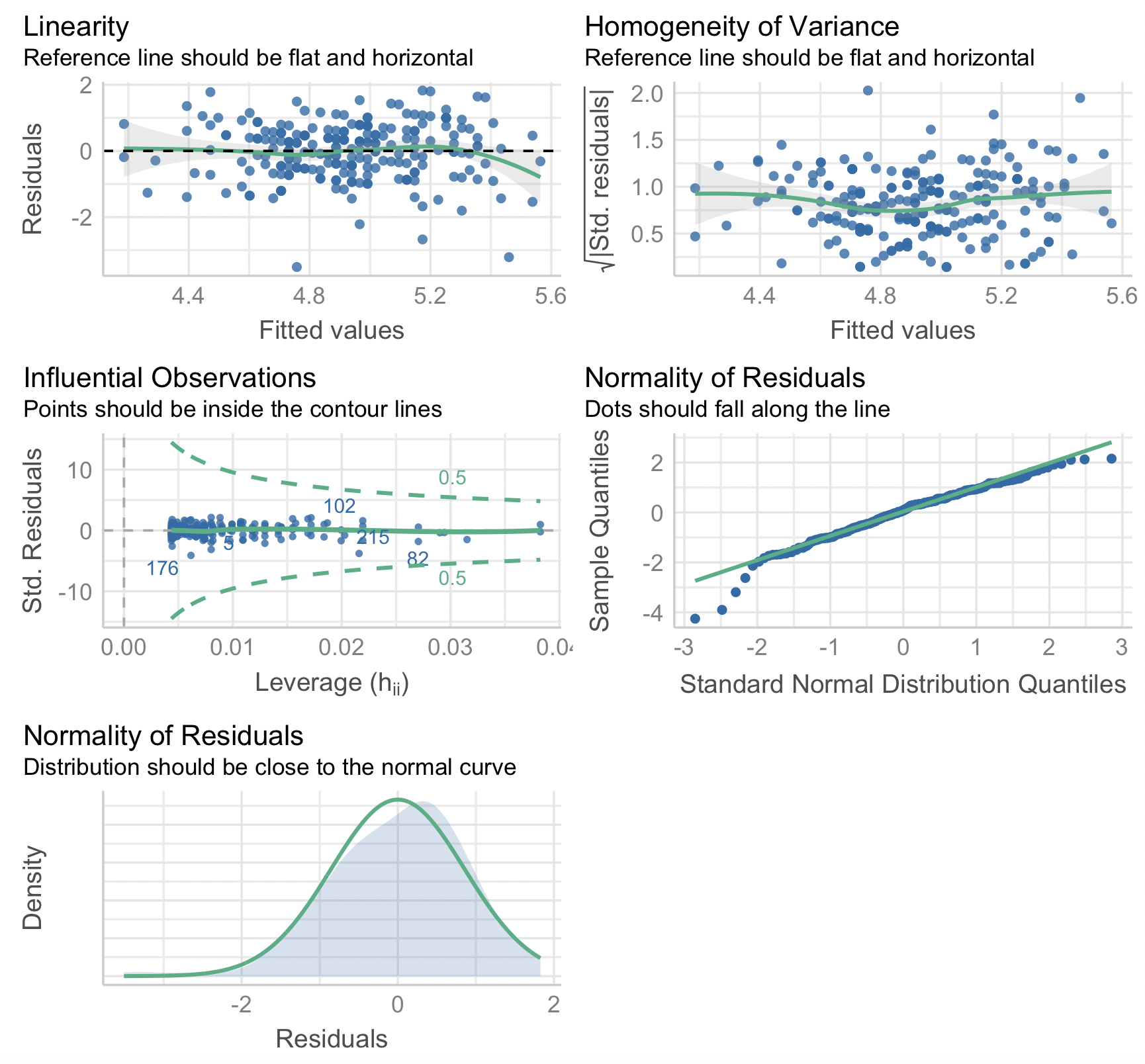


**For R1:**


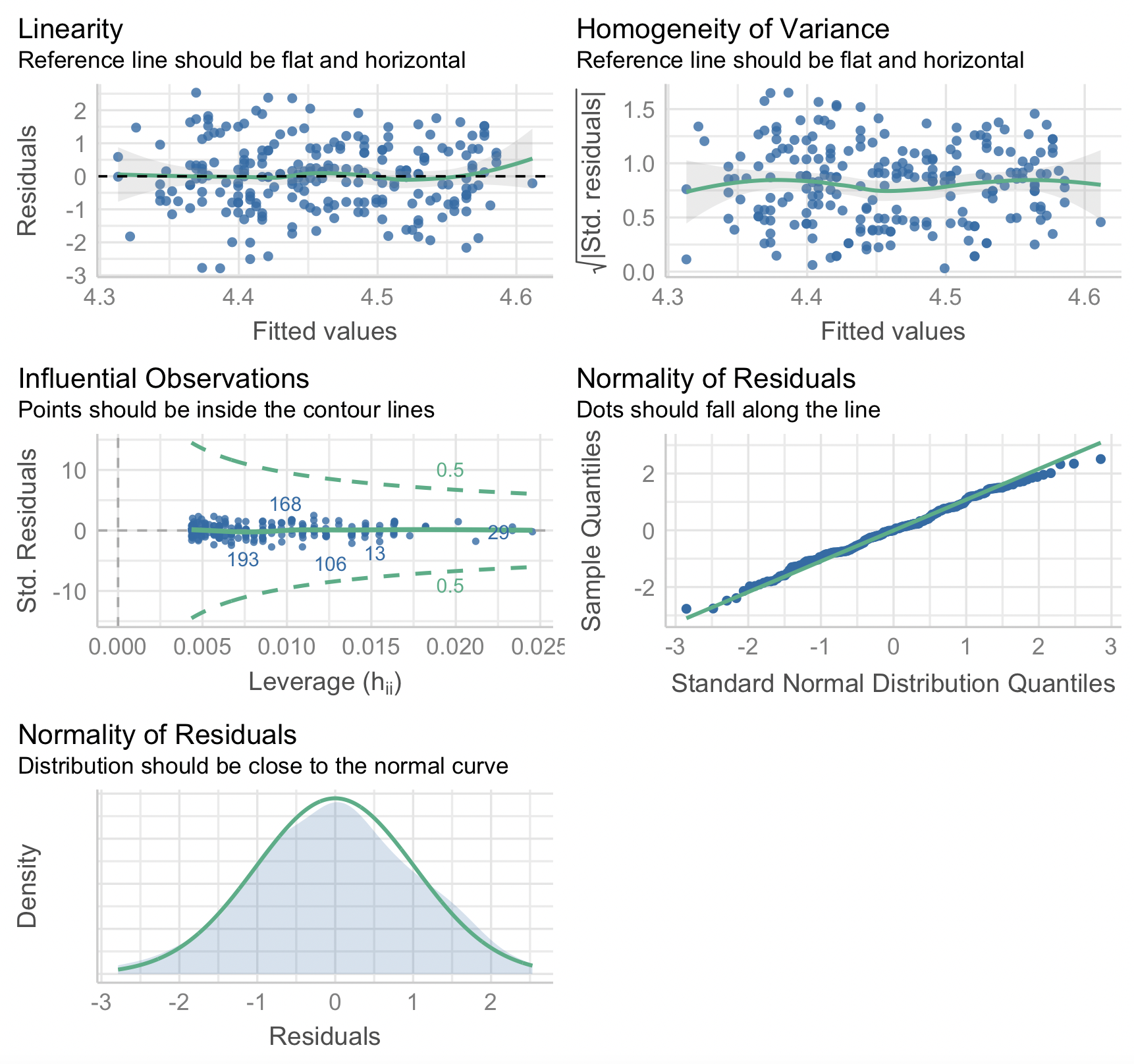


**For P2:**

**
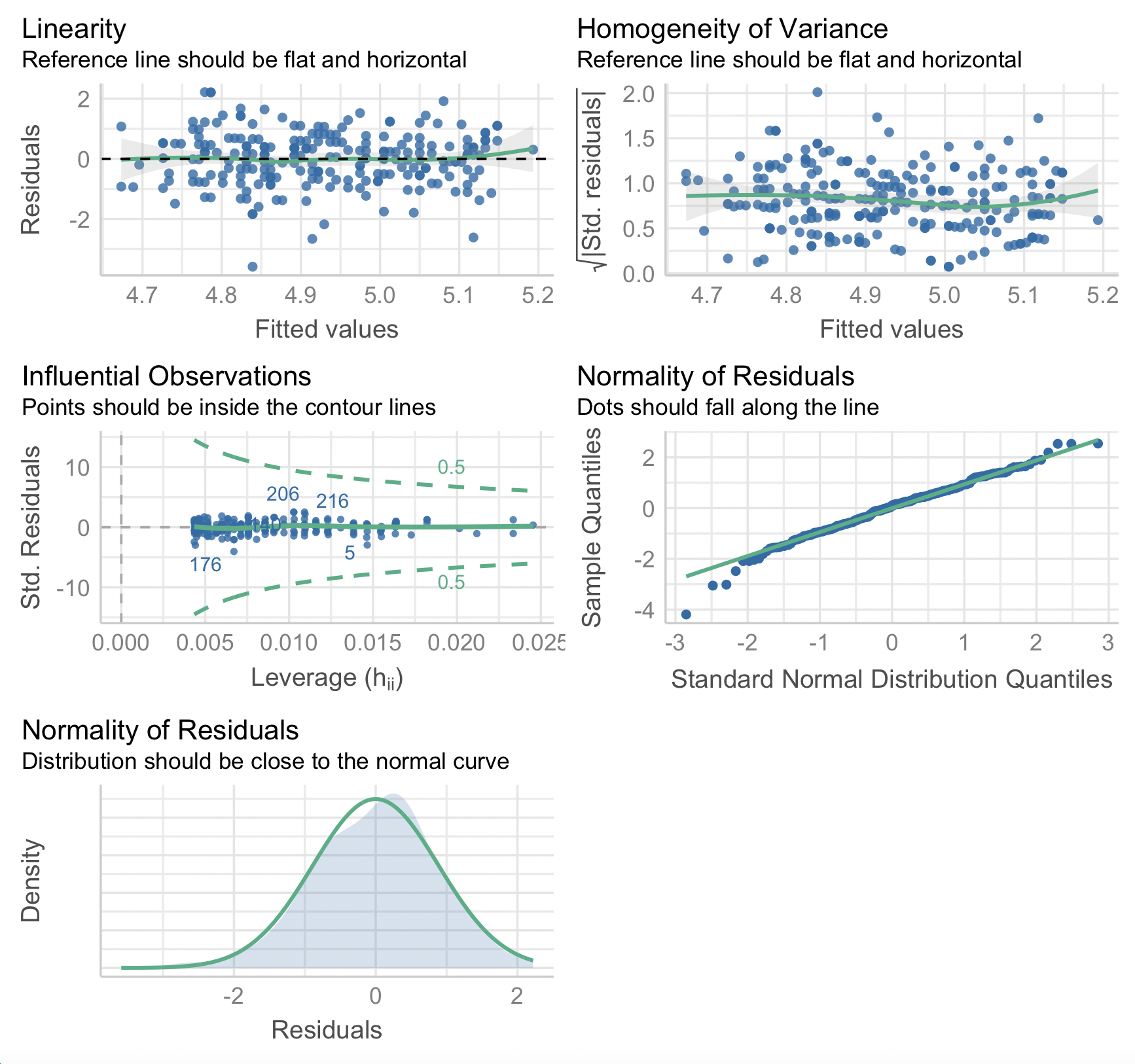
**

**For R2:**

**
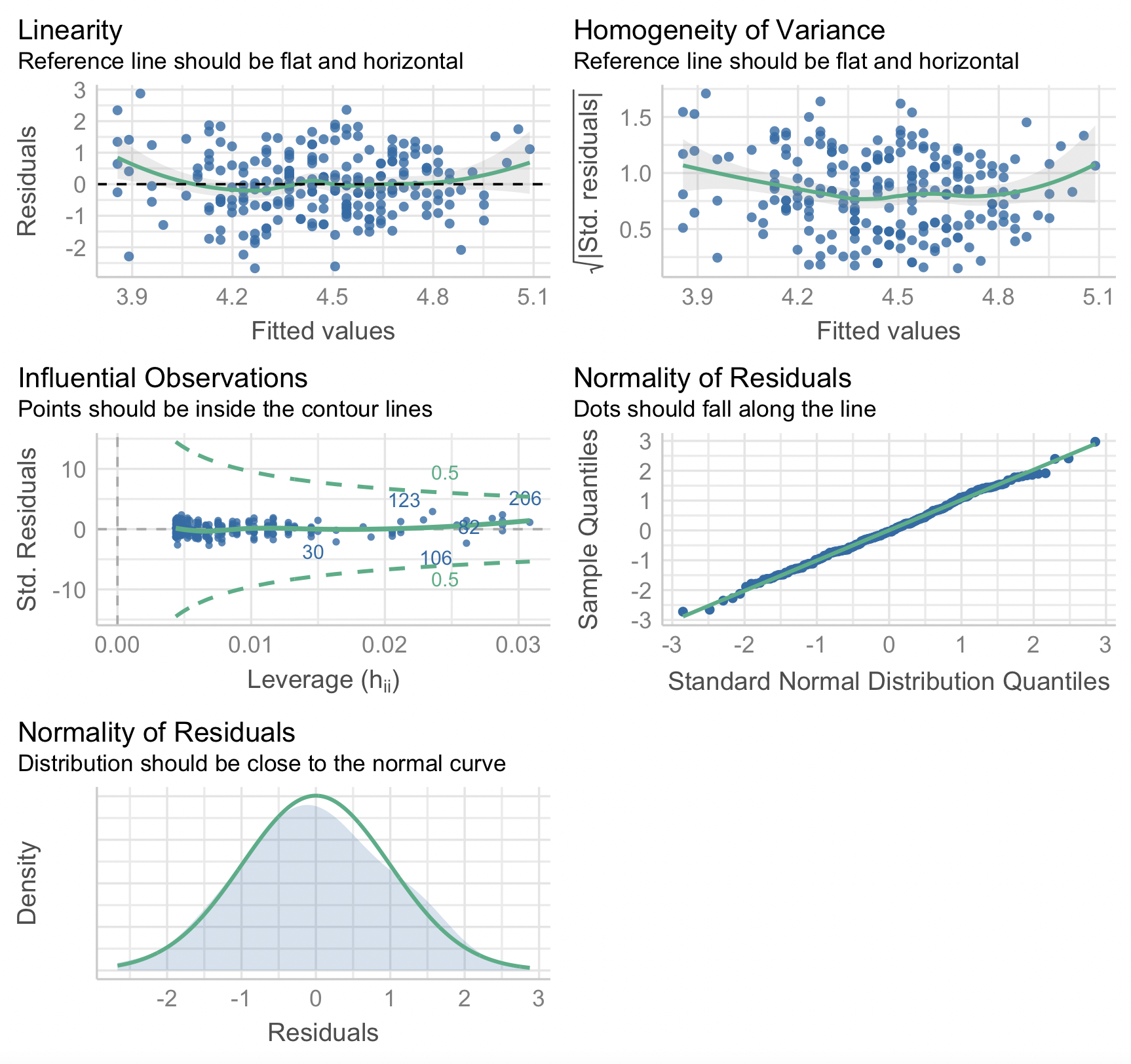
**

**For RQ2:**

**
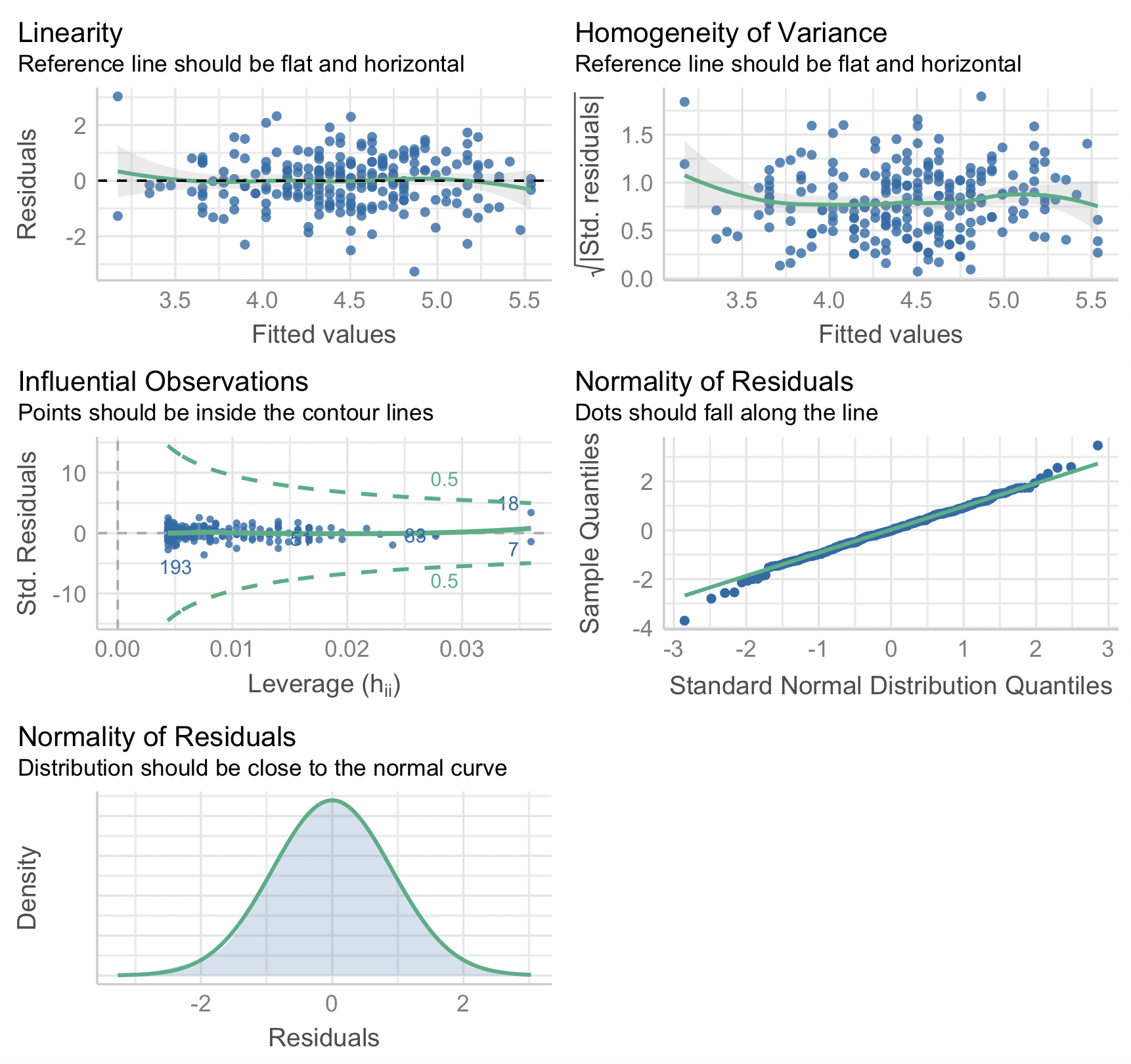
**

**For P6:**

**
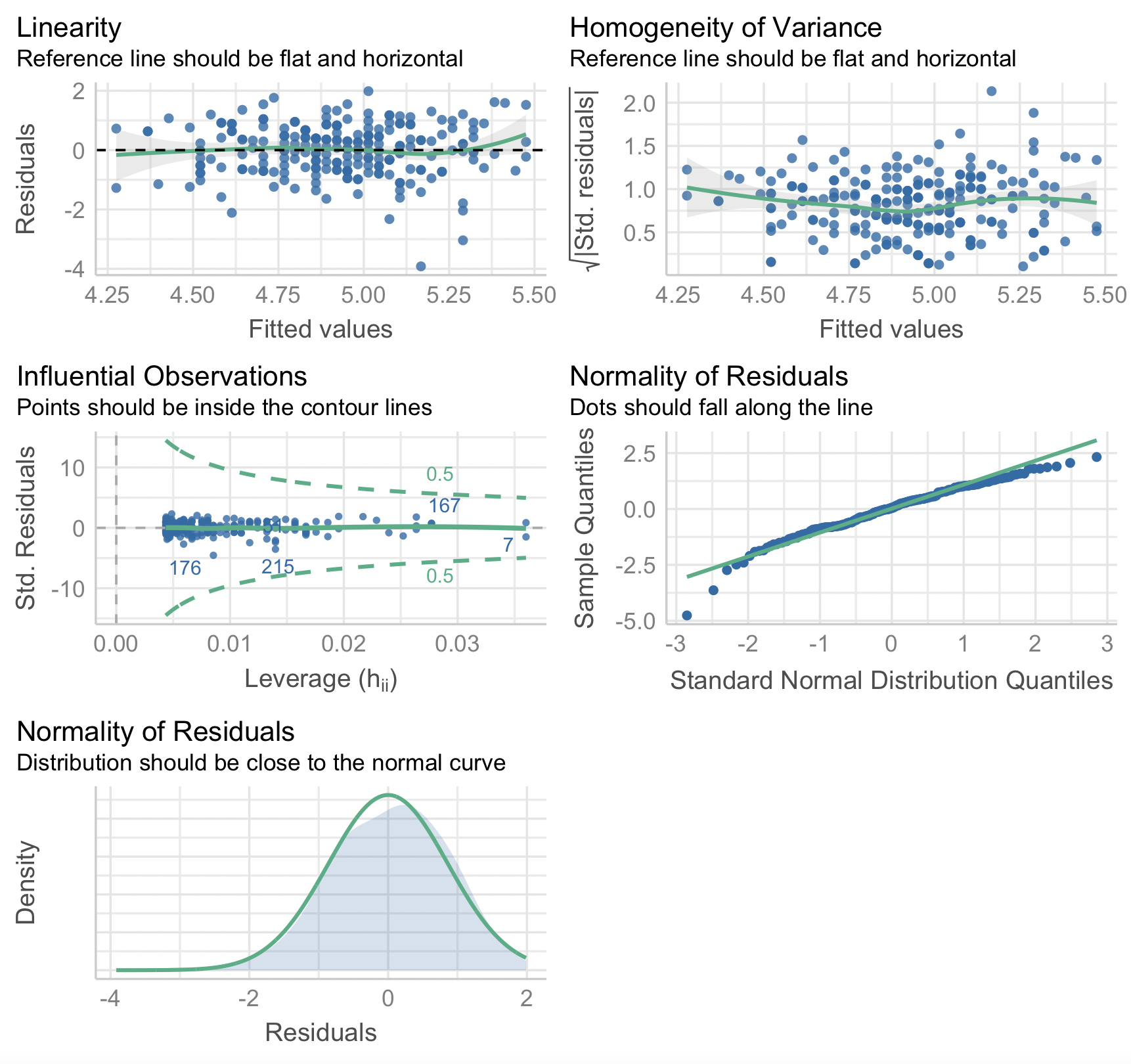
**

**For P7:**

**
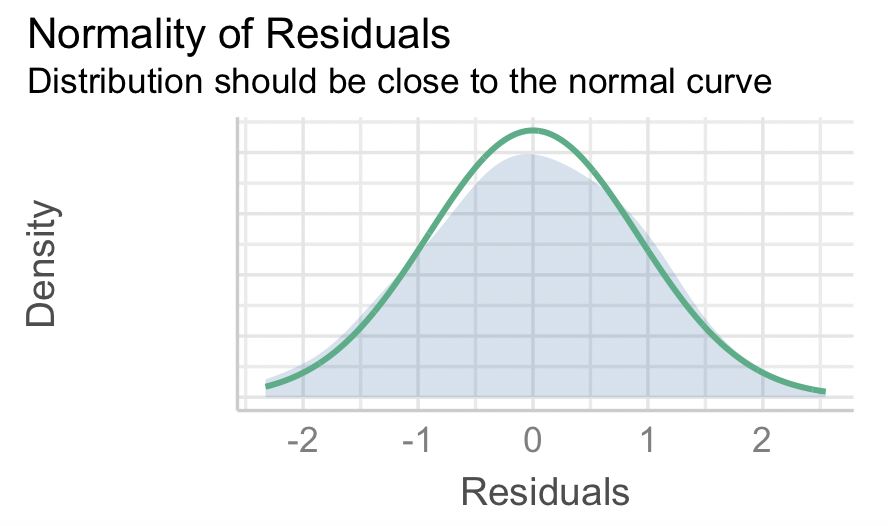
**

**For P9**:

**
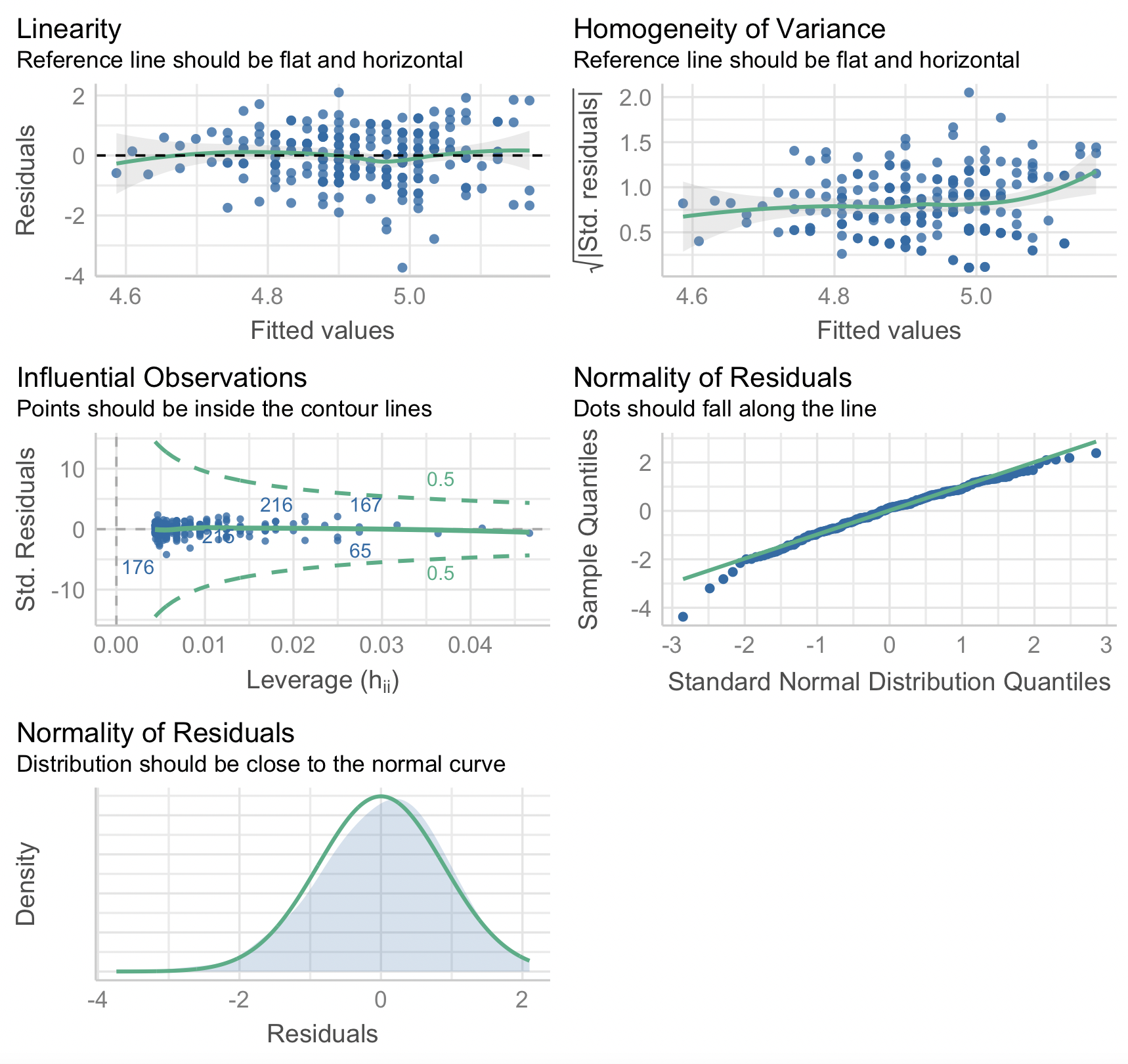
**

**For R4:**

**
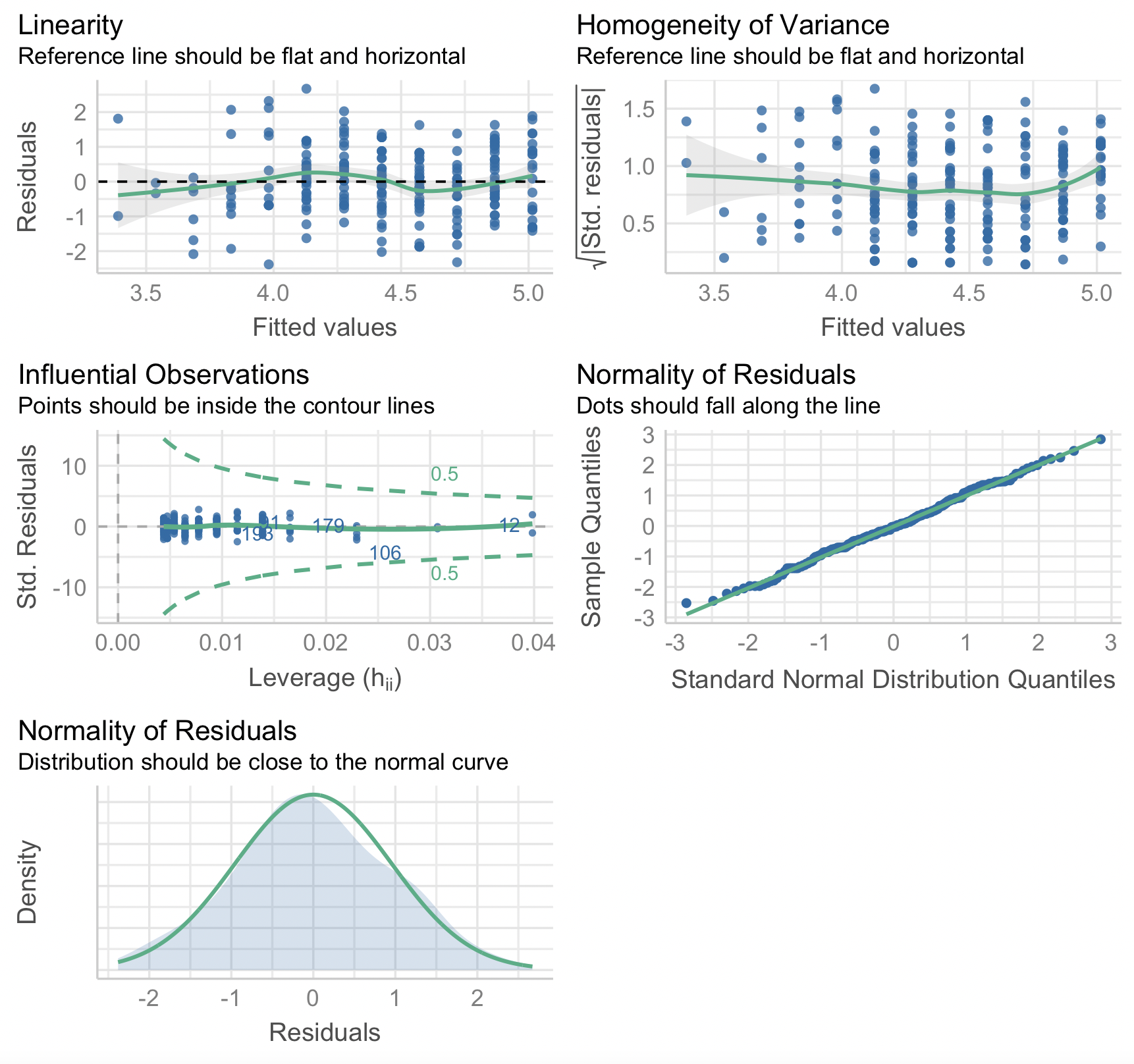
**

**For P10:**

**
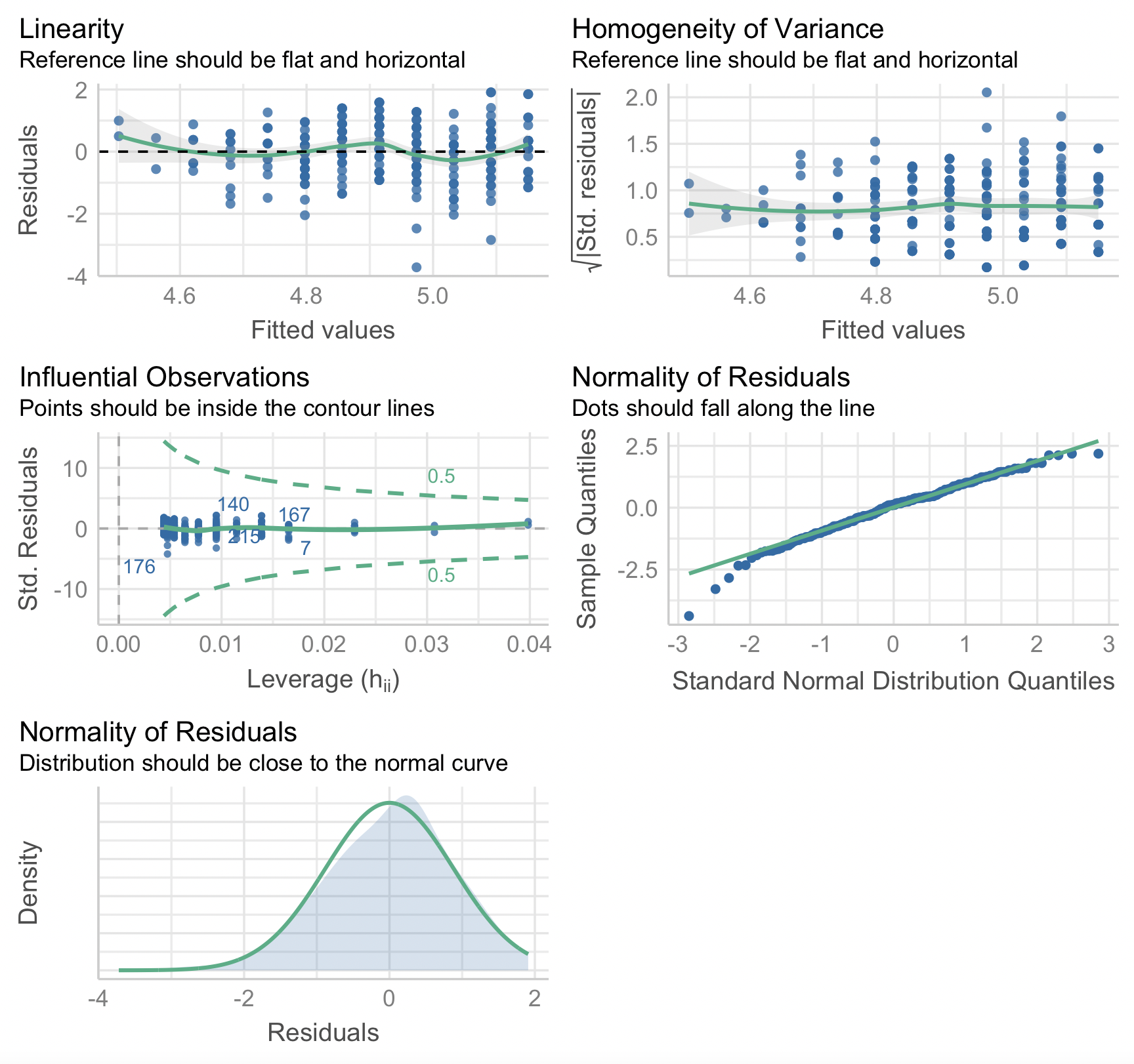
**
